# New Cembranoids and a Biscembranoid Peroxide from the Soft Coral *Sarcophyton cherbonnieri*

**DOI:** 10.3390/md16080276

**Published:** 2018-08-06

**Authors:** Chia-Chi Peng, Chiung-Yao Huang, Atallah F. Ahmed, Tsong-Long Hwang, Chang-Feng Dai, Jyh-Horng Sheu

**Affiliations:** 1 Department of Marine Biotechnology and Resources, National Sun Yat-sen University, Kaohsiung 804, Taiwan; m045020008@student.nsysu.edu.tw (C.-C.P.); huangcy@mail.nsysu.edu.tw (C.-Y.H.); 2 Department of Pharmacognosy, College of Pharmacy, King Saud University, Riyadh 11451, Saudi Arabia; afahmed@ksu.edu.sa; 3 Department of Pharmacognosy, Faculty of Pharmacy, Mansoura University, Mansoura 35516, Egypt; 4 Graduate Institute of Natural Products, College of Medicine, Chang Gung University, Taoyuan 333, Taiwan; htl@mail.cgu.edu.tw; 5 Research Center for Industry of Human Ecology and Graduate Institute of Health Industry Technology, Chang Gung University of Science and Technology, Taoyuan 333, Taiwan; 6 Department of Anesthesiology, Chang Gung Memorial Hospital, Taoyuan 333, Taiwan; 7 Institute of Oceanography, National Taiwan University, Taipei 112, Taiwan; corallab@ntu.edu.tw; 8 Graduate Institute of Natural Products, Kaohsiung Medical University, Kaohsiung 807, Taiwan; 9 Frontier Center for Ocean Science and Technology, National Sun Yat-sen University, Kaohsiung 804, Taiwan

**Keywords:** *Sarcophyton cherbonnieri*, cembranoid, biscembranoid peroxide, anti-inflammatory activity, elastase release inhibition

## Abstract

Six new cembranoids, cherbonolides A−E (**1**–**5**) and bischerbolide peroxide (**6**), along with one known cembranoid, isosarcophine (**7**), were isolated from the Formosan soft coral *Sarcophyton cherbonnieri*. The structures of these compounds were elucidated by detailed spectroscopic analysis and chemical methods. Compound **6** was discovered to be the first example of a molecular skeleton formed from two cembranoids connected by a peroxide group. Compounds **1**–**7** were shown to have the ability of inhibiting the production of superoxide anions and elastase release in *N*-formyl-methionyl-leucyl-phenylalanine/cytochalasin B (fMLF/CB)-induced human neutrophils.

## 1. Introduction

Many cembrane-based natural products have been shown to exhibit significant activities such as cytotoxicity [1,2,3,4,5,6,7,8,9,10,11,12,13,14] and anti-inflammatory activity [9,11,13,14,15,16,17,18]. From the experience of searching bioactive metabolites from soft corals, series of cembranoids have been unveiled from octocorals (Alcyonaceae) belonging to the genera *Sarcophyton*, [1,2,3,4,5,6,7,8,16], *Sinularia* [9,10,11,12,17,18] and *Lobophyton* [13,14,15]. Also, previous studies showed that two cembranoid units could form biscembranoid-type compounds by Diels-Alder reaction [19,20,21], radical dimerization [22,23], or connection with a sulfur atom [18], making the chemistry of cembranes more complex and interesting than monomeric form.

**Figure 1:**
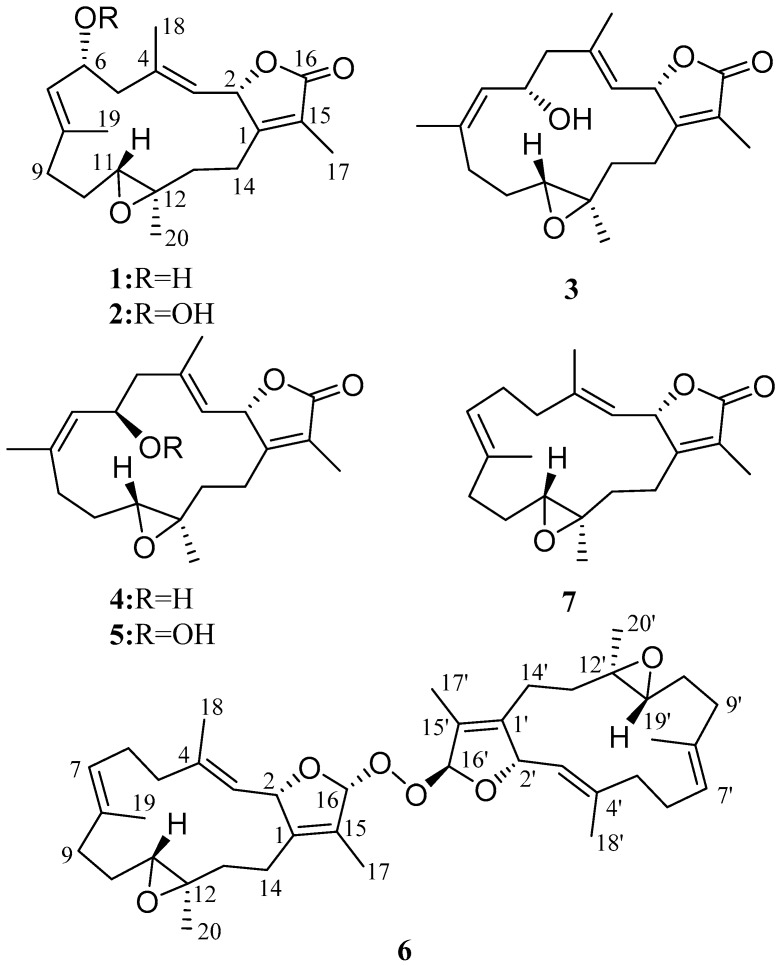
Cembranoid isolated from *Sarcophyton cherbonnieri.*

Our current chemical investigation on *Sarcophyton cherbonnieri* led to the discovery of six new cembranoids, cherbonolides A−E (**1**–**5**) and bischerbolide peroxide (**6**), and one known compound, isosarcophine (**7**) [24]. The structures of **1**–**7** (Figure 1) were elucidated by extensive spectroscopic analysis, including detailed 2D nuclear magnetic resonance (NMR) experiments and chemical methods. Compounds **2**, **5**, and **6** were characterized as cembranoids bearing an allylic peroxyl group as those previously discovered marine cembranoidal peroxides [25,26,27,28,29]. Furthermore, compound **6** is the first example of a biscembranoid with two cembranoidal units interconnected by a peroxyl group. The absolute configurations of **1** and **3** were further established using a modified Mosher’s reaction. Also, evaluation of the in vitro anti-inflammatory activities through the inhibition of superoxide anion (O_2_^−•^) generation and elastase release in *N*-formyl-methionyl-leucyl-phenylalanine/cytochalasin B (fMLF/CB)-induced human neutrophils was carried out.

## 2. Results and Discussion

The soft coral *S. cherbonnieri* (1.2 kg, wet weight) was collected using SCUBA diving from Jihui Port of Taitung, Taiwan, in March 2013, and stored in a freezer before extraction. The freeze-dried organisms (207 g) were sliced into small pieces, followed by exhaustive extraction with ethyl acetate (EtOAc). The EtOAc extract was dried with anhydrous sodium sulfate (Na_2_SO_4_). After removal of EtOAc under reduced pressure, the residue yielded was separated by silica gel column chromatography and the resolved fractions were further purified by reverse-phase C_18_ high-performance liquid chromatography (HPLC) to afford compounds **1**–**7** (Figure 1), the structures of which were elucidated on the basis of spectroscopic analyses (Supplementary Materials, Figures S1–S46).

**Figure 2:**
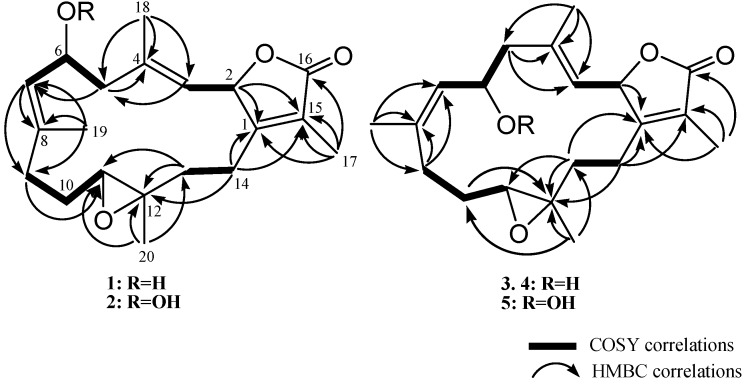
Selected ^1^H-^1^H COSY and HMBC correlations of **1**, **2** and **3**−**5**.

Cherbonolide A (**1**) was isolated as a colorless oil. The molecular formula C_20_H_28_O_4_ of **1** was determined by the high-resolution electrospray ionization mass spectrometry (HRESIMS) (*m*/*z* calcd 355.1880; found 355.1879, [M + Na]^+^), which required seven degrees of unsaturation. The IR spectrum of **1** showed the presence of a hydroxyl group (*ν*_max_ 3457 cm^−1^) and a lactonic carbonyl group (*ν*_max_ 1746 cm^−1^). The presence of 20 carbons in the structure of **1**, including four methyls, five *sp^3^* methylenes, three *sp^3^* oxygenated methines, two *sp^2^* methines, one *sp^3^* and five *sp^2^* nonprotoned carbon atoms were assigned with the aid of distortionless enhancement by polarization transfer (DEPT) spectra. The NMR peaks resonating at δ_C_ 174.4 (C), 160.7 (C), 123.8 (C), 77.8 (CH) and 8.8 (CH_3_), and δ_H_ 5.44 (1H, dd, *J* = 10.0, 1.6 Hz) and δ_H_ 1.86 (3H, s), are characteristic signals of an *α*-methyl-*α,β*-unsaturated-*γ*-lactone ring by comparison of the NMR data of the *γ*-lactone ring of known compound isosarcophine (**7**). Signals at δ_C_ 60.8 (C), 61.4 (CH) and δ_H_ 2.42 (1H, dd, *J* = 10.8, 2.8 Hz) showed the presence of a trisubstituted epoxide. Two trisubstituted double bonds could also be identified by NMR signals resonating at δ_C_ 122.2 (CH), δ_C_ 141.6 (C) and δ_H_ 4.90 (1H, d, *J* = 10.0 Hz), and at δ_C_ 128.1 (CH), δ_C_ 139.8 (C) and δ_H_ 5.20 (1H, d, *J* = 10.4 Hz), respectively. The correlations identified from the ^1^H-^1^H correlation spectroscopy (^1^H-^1^H COSY) spectrum revealed four separate proton sequences, as shown in Figure 2, which were assembled by heteronuclear multiple bond correlation (HMBC) correlations (Figure 2). Key HMBC correlations of H-2 (δ_H_ 5.44, 1H, dd, *J* = 10.0, 1.6 Hz) to C-1; H_2_-14 (δ_H_ 2.01, m)/C-1; H_3_-17 (δ_H_ 1.86, s) to C-1, C-15 and C-16; H_3_-18 (δ_H_ 1.70, s) to C-3, C-4 and C-5; H_3_-19 (δ_H_ 1.86, s) to C-7, C-8 and C-9; and H_3_-20 (δ_H_ 1.33, s) to C-11, C-12 and C-13, established the connection of the four proton sequences, and thus constructed the 14-membered ring carbon skeleton, which also indicated the presence of a hydroxyl at C-6. Thus, the planar structure of **1** was established.

**Figure 3:**
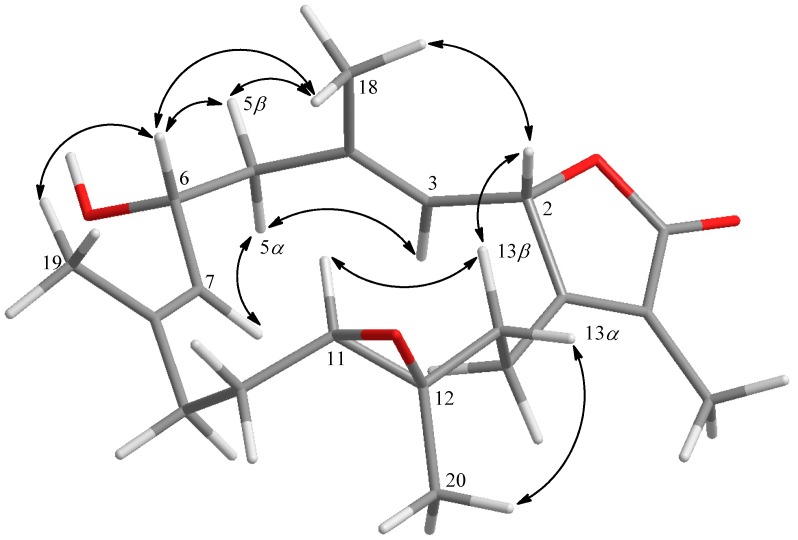
Key NOESY correlations of **1**.

**Figure 4:**
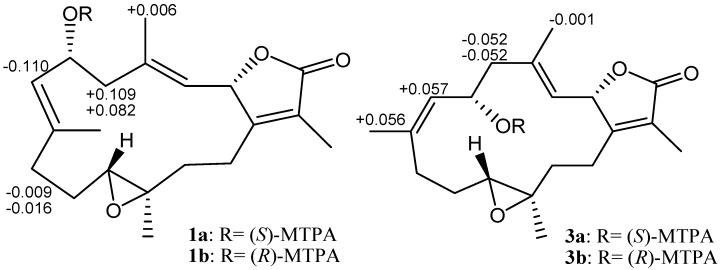
^1^H NMR chemical shift differences Δδ (δ_S_ − δ_R_) in ppm for the MTPA esters of **1** and **3**.

Further, careful analysis of nuclear Overhauser enhancement (NOE) correlations was applied to establish the relative stereochemistry of **1**, as shown in Figure 3. The NOE spectrum revealed that H-2 (δ_H_ 5.44, dd, *J* = 10.0, 1.6 Hz) showed NOE correction with H_3_-18 (δ_H_ 1.70, s); therefore, assuming a *β*-orientation of H-2, H_3_-18 should be *β*-oriented, too. Moreover, H_3_-18 exhibited NOE correlation with H-6 (δ_H_ 4.70, ddd, *J* = 10.4, 10.4, 5.2 Hz), revealing the *β*-orientation of H-6 and the R* configuration of C-6. One methylene proton at C-13 exhibited NOE correlation with H-2 and was characterized as H-13*β* (δ_H_ 1.06, m), while the other proton was assigned as H-13α (δ_H_ 2.03, m). NOE correlations of H-13*β* with H-11 (δ_H_ 2.42, dd, *J* = 10.8, 2.8 Hz) and H-13α with H_3_-20 (δ_H_ 1.33, s) reflected the *β*-orientation of H-11 and the α-orientation of H_3_-20, and hence the R* configurations of both C-11 and C-12. The *E* geometries of the trisubstituted C-3/C-4 and C-7/C-8 double bonds were also assigned from the NOE correlations of H_3_-18 (δ_H_ 1.70, s) with H-2, but not with H-3 (δ_H_ 4.90, d, *J* = 10.0 Hz), as well as H_3_-19 (δ_H_ 1.86, s) with H-6, but not with H-7 (δ_H_ 5.20, d, *J* = 10.4 Hz), and were also confirmed by the upfield chemical shifts (δ_C_ < 20 ppm) observed for both C-18 (δ_C_ 15.9) and C-19 (δ_C_ 14.9) [30]. Based on the above observations and the detailed analysis of other NOE correlations, the relative configuration of this compound was established. Furthermore, the absolute configuration of **1** at C-6 was determined by the modified Mosher’s esterification method [31,32]. The (*S*)- and (*R*)-MTPA esters of **1** (**1a** and **1b**, respectively, as shown in Figure 4) were afforded by the reaction of **1** with (*R*)-(-) and (*S*)-(+)-MTPA chloride, respectively. Determination of the Δδ values (δ_S_–δ_R_) for protons nearing C-6 resulted in the establishment of the *R* configuration at C-6 in **1** (Figure 4). The absolute configuration of **1** was thus assigned as 2*S*,6*R*,11*R*,12*R*, mostly the same as that of isosarcophine (**7**) [24], except that of C-6. Therefore, cherbonolide A (**1**) was identified as 6-*α*-hydroxyisosarcophine.

The molecular formula of cherbonolide B (**2**) was determined to be C_20_H_28_O_5_ by the HRESIMS (*m*/*z* calcd 371.1830; found 371.1829, [M + Na]^+^), having one more oxygen than 1. Moreover, both **1** and **2** had almost identical ^1^H and ^13^C NMR data (Table 1), except for those of C-6. The allylic hydroxy group of **1** at C-6 was substituted by a hydroperoxyl in **2**, with the characteristic signal of a broad singlet in the downfield region, δ_H_ 7.99 [26,33,34]. Obvious downfield shifts of C-6 (δ_C_ 65.2 in **1**, 78.3 in **2**) and H-6 (δ_H_ 4.70 in **1**, 4.97 in **2**) were also observed, indicating that **2** possesses the hydroperoxy group at C-6. Furthermore, reduction of **2** by reaction with triphenylphosphine afforded **1**. On the basis of the above analyses, the planar structure and the (2*S*,6*R*,11*R*,12*R*)-configuration of **2** were determined.

**Figure 5:**
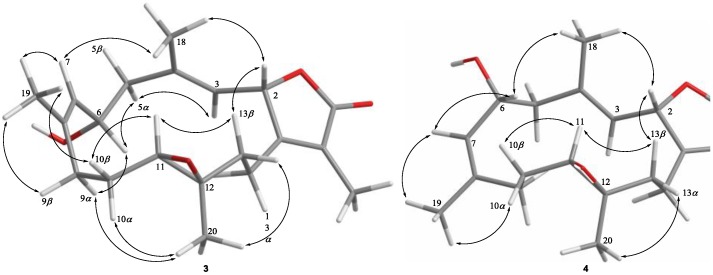
Key NOESY correlations for **3** and **4**.

Cherbonolide C (**3**) should have the same molecular formula as **1**, according to HRESIMS data. Also, the ^1^H-^1^H COSY and HMBC correlations (Figure 2) of **3** are similar to those of **1**, suggesting that these compounds possess almost the same molecular skeleton. Analysis of NOE correlations (Figure 5) showed that the relative configurations at C-2, C-11 and C-12 in **1** and **3** are the same. Assuming the *β*-orientation of H-2, as H_3_-18 showed NOE interaction with H-2 but not with H-3, the *E* geometry was assigned to the trisubstituted C-3/C-4 double bond. One of the methylene protons at C-5 (δ_H_ 2.42, dd, *J* = 12.0, 3.2 Hz) displayed NOE interaction with H-3, but not with H_3_-18, and was hence determined as H-5α. Further, H-6 (δ_H_ 3.84, dd, *J* = 9.2, 9.2 Hz) showed NOE correlations with H-5*α* and H-9*α*, but not with H-9*β* and H_3_-19. These observations, together with NOE correlations of H-9*β*/H_3_-19, H_3_-19/H-7 and H-7/H_3_-18, enabled deduction of the *α*-orientation of H-6 and led to the assignment of a 6S* configuration and a *Z* geometry of the trisubstituted C-7/C-8 double bond in **3**. The olefinic methyl group attaching at C-8 showed carbon signal at δ_C_ 22.2 ppm further confirmed the *Z* geometry of C-7/C-8 double bond [30]. The absolute configuration of **3** at C-6 was also verified by using the modified Mosher’s method. Determination of the Δδ values (δ_S_ − δ_R_, shown in Figure 4) for protons neighboring C-6 further confirmed the *S* configuration at C-6 in **3** (Figure 4). The absolute configuration of **3** was thus assigned as 2*S*,6*S*,11*R* and 12*R*. Thus, cherbonolide C (**3**) is the 7*Z* isomer of cherbonolide A (**1**).

Cherbonolide D (**4**) was found to be an isomer of **3** according to HRESIMS. Both compounds have almost the same ^1^H-^1^H COSY and HMBC correlations, indicating they have the same molecular skeleton. NMR data of **3** and **4** are nearly the same (Table 1), except for those of CH-6, suggesting that **4** could be the C-6 epimer of **3**. The (2*S*,6*R*,11*R*,12*R*)-configuration and the *E* and *Z* geometries of C-3/C-4 and C-7/C-8 double bonds of **4**, respectively, were also established by analysis of NOE correlations to be as those of **3** (Figure 5).

Cherbonolide E (**5**) was determined to have a molecular formula C_20_H_28_O_5_ from its HRESIMS data (*m*/*z* calcd 371.1830; found 371.1829, [M + Na]^+^), with one more oxygen than in **4**. Compounds **4** and **5** displayed almost identical ^1^H and ^13^C NMR data (Table 2), except for those of CH-6. It was found that the hydroxy substituent of **4** at C-6 was replaced by a hydroperoxy group in **5**, with the characteristic signal of a broad singlet at δ_H_ 7.25 [26,33,34]. The obvious downfield shifts of C-6 (δ_C_ 64.8 in **4**, 78.9 in **5**) and H-6 (δ_H_ 4.21 in **4**, 4.58 in **5**) also confirmed the substitution of a hydroperoxy group at C-6 of **5**. Furthermore, reduction of **5** with triphenylphosphine could afford **4**. Therefore, the structure of **5**, with the (2*S*,6*R*,11*R*,12*R*)-configuration, was determined.

Bischerbolide peroxide (**6**) was afforded as a white powder with the molecular formula C_40_H_58_O_6_ from HRESIMS (*m/z* calcd 657.4124; found 657.4125, [M + Na]^+^), appropriate for twelve degrees of unsaturation. The ^13^C NMR spectroscopic data of **6** revealed the presence of 40 carbons (Table 2). The DEPT spectra of **6** showed the presence of eight methyls, twelve *sp^3^* methylenes, six *sp^3^* oxygenated methines, four *sp^2^* methines, two *sp^3^* and eight *sp^2^* nonprotoned carbons (including two ester carbonyls). NMR signals resonating at δ_C_ 114.3 (CH), 141.4 (C), 124.9 (C), 82.7 (CH) and 10.2 (CH_3_), and δ_H_ 5.28 (1H, d, *J* = 10.0 Hz) and δ_H_ 1.72 (3H, s), and another group of signals observed at δ_C_ 114.4 (CH), 141.5 (C), 124.9 (C), 81.9 (CH) and 10.2 (CH_3_), and δ_H_ 5.50 (1H, d, *J* = 10.0 Hz) and δ_H_ 1.73 (3H, s), revealed the presence of two slightly different 2,5-dihydrofuran rings with a peroxyl group by comparison of the similar NMR data of five-membered rings in the literature [35]. Also, two groups of signals resonating at δ_C_ 61.2 (C), 62.1 (CH) and δ_H_ 2.51 (1H, m), and δ_C_ 61.3 (C), 62.2 (CH) and δ_H_ 2.51 (1H, m) showed the presence of two trisubstituted epoxides. Four trisubstituted olefinic bonds were revealed from NMR signals appearing at δ_C_ 126.4 (CH), δ_C_ 140.2 (C) and δ_H_ 5.06 (1H, d, *J* = 10.0 Hz); at δ_C_ 125.6 (CH), δ_C_ 133.1 (C) and δ_H_ 4.98 (1H, dd, *J* = 9.2, 9.2 Hz); at δ_C_ 125.1 (CH), δ_C_ 141.0 (C) and δ_H_ 4.92 (1H, dd, *J* = 10.0 Hz); and at δ_C_ 125.5 (CH), δ_C_ 133.3 (C) and δ_H_ 4.95 (1H, dd, *J* = 9.2, 9.2 Hz), respectively.

**Figure 6:**
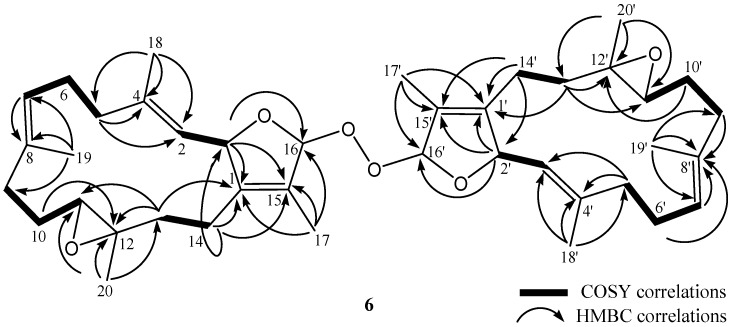
Selected ^1^H-^1^H COSY and HMBC correlations of **6**.

**Figure 7:**
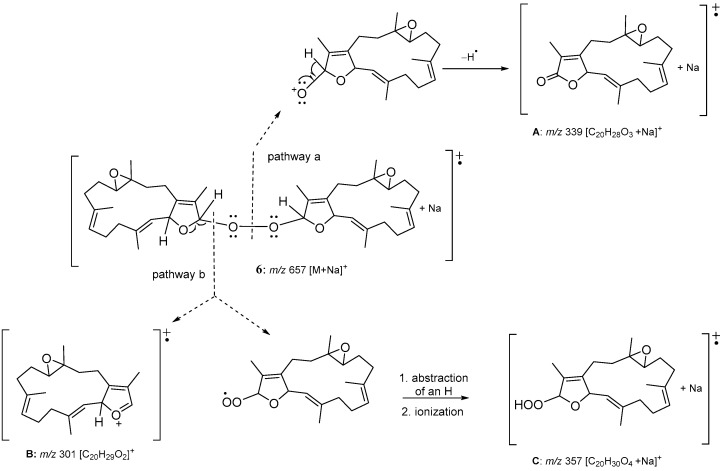
ESIMS fragmentation of **6**.

As the ^13^C NMR spectrum of **6** was constituted by twenty sets of signals with each set containing two peaks of very similar chemical shifts, **6** was thus identified as a compound formed from the connection of two quite similar diterpenoid subunits. The entire planar structure was established by examination of ^1^H and ^13^C NMR data and ^1^H-^1^H COSY and HMBC correlations (Figure 6). Two methines resonating at δ_C_ 114.3 and δ_C_ 114.4 were considered to be the positions at which the two cembranoidal units were connected by insertion of a peroxyl group. Based on the above analyses, the molecular skeleton of **6** was elucidated as the biscembranoid formed by the connection of two molecules of isosarcophytoxide [36] via a peroxyl group at C-16 and C-16′. The fragmentation pattern of ESIMS (Figure 7) could further prove the dimeric nature of **6** and the peroxyl linkage at C-16/C-16′. One ion peak displayed at *m/z* 339 can be explained by the cleavage of O–O bond and the following elimination of H-16 from a monocembranoidal unit in **6** to form a sodiated cembranoid lactone molecular ion A (pathway a). The other ion peaks can be interpreted by the cleavage of the single bond between C-16 and peroxyl oxygen to afford ion B (*m/z* 301), and a peroxycembranoidal radical which could further abstract an hydrogen atom and form the sodium adduct C (*m/z* 357) (pathway b). Moreover, compound **6** was found to be the first example of a biscembranoid with a molecular skeleton formed by two cembranoid units connected by a peroxyl group.

**Figure 8:**
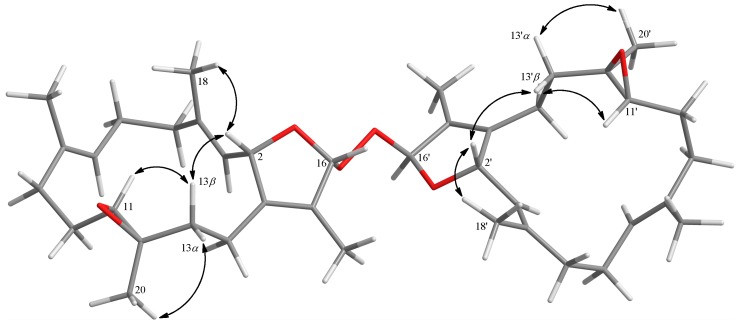
Selected NOESY correlations for **6**.

**Figure 9:**
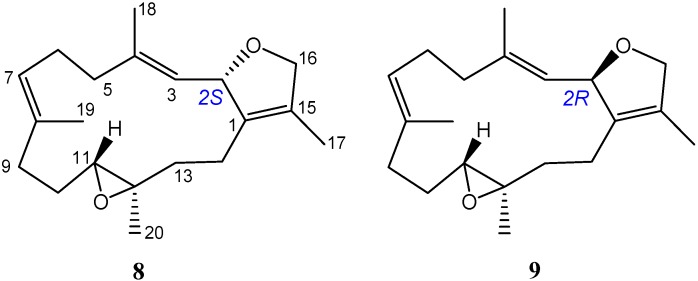
Structures of (2*S*,11*R*,12*R*)-isosarcophytoxide (**8**) and (2*R*,11*R*,12*R*)-isosarcophytoxide (**9**) [36].

The relative configuration of **6** was determined from a literature survey [36,37] and NOE correlations (Figure 8). The ^13^C NMR spectrum of **6** displayed 40 signals of two sets signals with nearly identical chemical shifts, representing the very similar stereochemical environments of the two structural units. In addition, compound **6** was found to have nearly identical chemical shifts for H-11 (11′), H_3_-18 (18′), H_3_-20 (20′) and C-20 (20′) to those of (2*S*,11*R*,12*R*)-isosarcophytoxide (**8**), and were in turn found to exhibit distinguishable differences to the corresponding chemical shifts of (2*R*,11*R*,12*R*)-isosarcophytoxide (**9**) (Table 3 and Figure 9). Thus, **6** possessed the cembranoidal structural unit derived from **8**, as also proven by observed NOE correlations (Figure 8). Different proton values were observed for H-2 (δ_H_ 5.28) and H-2′ (δ_H_ 5.50), indicating that H-2′ was on the same planar face as the peroxide group and was deshielded, and H-2 was on the same planar face as H-16 and was shielded. As compounds **1**−**7** were isolated from the same organism in this study, they are likely to possess the same absolute *S*,*R*,*R*-configurations at the chiral centers C-2, C-11 and C-12, respectively, as those of **1** and **3**. A previous report also showed that different absolute configurations at C-2 of the related diasteromeric dihydrofuran ring-containing cembranoids could significantly influence the sign of the specific optical rotation [36,38]. For cembranoids with 2*S* configuration a significant positive and for those with 2*R* configuration a negative optical rotation were found. The ${\left[\alpha \right]}_{D}^{25}$ of **6** was +41; thus, the absolute configuration of **6** was deduced to be 2*S*,11*R*,12*R*,16*R*, 2′*S*,11′*R*,12′*R*,16′*S*.

**Scheme 1:**
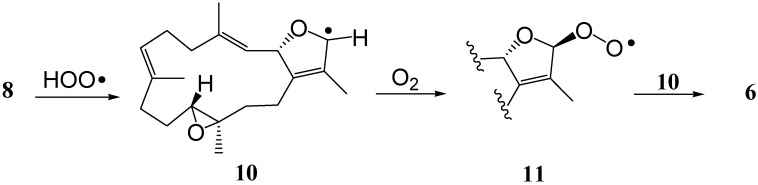
Proposed biosynthetic pathway for **6**.

The plausible biosynthesis of **6** might arise from the proton abstraction at C-16 of **8** by hydrogen peroxide radical HOO• to form a radical intermediate **10**, which could react with O_2_ from one plane side of radical center C-16 to afford cembranoidal peroxide radical **11**. Further reaction of **11** with **10** from another side could lead to the formation of **6** (Scheme 1). However, the possibility that **6** might be generated by autooxidation of **8** could not be neglected.

It is known that the proteolytic enzymes and toxic reactive oxygen species produced by stimulated neutrophils might play a critical role in the pathogenesis of many inflammatory diseases [39,40]. By measuring the capability to inhibit *N*-formyl-methionyl-leucyl-phenylalanine/cytochalasin B (fMLF/CB)-induced superoxide anion generation and elastase release in human neutrophils, the in vitro anti-inflammatory effects for metabolites **1**–**7** were evaluated [41,42]. According to the results (shown in Table 4), compound **6** had a significant inhibitory effect (64.6 ± 0.8%), with an IC_50_ value of 26.2 ± 1.0 μM, on the generation of superoxide anions, and compounds **1** and **3** had moderate inhibitory effects (32.1 ± 4.3% and 44.5 ± 4.6%, respectively) at 30 μM. Compounds **1**, **3** and **6** revealed moderate inhibitory effects (37.6 ± 5.0%, 35.6 ± 6.2% and 42.4 ± 5.1%, respectively) on elastase release at the same concentration. These results, obtained after stimualting the neutrophils with fMLF/CB, may suggest that **1**, **3** and **6** have potential merits against inflammatory disorders.

In summary, examination of the chemical constituents of the soft coral *Sarcophyton cherbonnieri* led to the discovery of six new compounds **1**–**6**, along with one known compound **7**. Although a number of natural compounds possessing a peroxyl group, such as artemisinin [43], neovibsanin C [44], cardamom peroxide [45], plakortin [46] and chondrillin [47], have been discovered, compound **6** was discovered to be the first compound with a molecular skeleton consisting of two cembranoidal units connected by a peroxide group. Similar to the results of previous studies indicating that natural peroxides could possess promising biological activity [48], compound **6** was found to possess anti-inflammatory activity by exhibiting stronger ability on inhibition on the generation of superoxide anions and release of elastase in fMLF/CB-induced human neutrophils.

## 3. Materials and Methods

### 3.1. General Procedures

The values of optical rotation of the metabolites were determined with a JASCO P-1020 polarimeter (JASCO Corporation, Tokyo, Japan). Infrared absorptions were recorded using a JASCO FT/IR-4100 infrared spectrophotometer (JASCO Corporation, Tokyo, Japan). ^1^H and ^13^C NMR spectra were obtained on a Varian 400MR FT-NMR (or Varian Unity INOVA500 FT-NMR) instrument (Varian Inc., Palo Alto, CA, USA) at 400 MHz (or 500 MHz) and 100 MHz (or 125 MHz), respectively, in CDCl_3_ or C_6_D_6_. The data of LRESIMS and HRESIMS were measured using a Bruker APEX II (Bruker, Bremen, Germany) mass spectrometer. Silica gel (230–400 mesh) was used as adsorbent for column chromatography. TLC analyses were performed using precoated silica gel plates (Kieselgel 60 F-254, 0.2 mm) (Merck, Darmstadt, Germany). Further purification of impure fractions or compounds were performed by high-performance liquid chromatography on a Hitachi L-7100 HPLC instrument (Hitachi Ltd., Tokyo, Japan) with a Merck Hibar Si-60 column (250 mm × 21 mm, 7 μm; Merck, Darmstadt, Germany) and on a Hitachi L-2455 HPLC apparatus (Hitachi, Tokyo, Japan) with a Supelco C18 column (250 mm × 21.2 mm, 5 μm; Supelco, Bellefonte, PA, USA).

### 3.2. Animal Material

The soft coral *S. cherbonnieri* was collected by hand using scuba diving from Jihui Fish Port, Taiwan, in March 2013, at a depth of 10–15 m. Organisms of the marine animal were stored in a freezer until extraction.

### 3.3. Extraction and Isolation

The frozen marine organisms, *S. cherbonnieri* (1.2 kg, wet wt), were freeze-dried (yield: 207 g), minced to small pieces and then extracted thoroughly with EtOAc (1 L × 5). The combined EtOAc extract (10.2 g) was concentrated under reduced pressure to yield a residue, which was chromatographed over a silica gel column by eluting with acetone in *n*-hexane (0–100%, stepwise), and then with MeOH in acetone (0–100%, stepwise) to yield 19 fractions. Fraction 9, eluting with *n*-hexane–acetone (6:1), was repeatedly purified by column chromatography over silica gel to yield a solid which was immersed in cold MeOH (0 °C) to afford a white powder **6** (24.3 mg). Fraction 10, eluting with *n*-hexane–acetone (4:1), was further purified over silica gel using *n*-hexane–acetone (6:1) to afford seven subfractions (A1–A7) and afford **7** (320.4 mg). Subfraction A2 was further separated by reverse-phase HPLC using acetonitrile–H_2_O (1:1.3) to afford **1** (11.0 mg). Subfraction A3 was purified by reverse-phase HPLC using acetonitrile–H_2_O (1:1.1) to afford **2** (13.3 mg) and **5** (10.1 mg). Fraction 13, eluting with *n*-hexane–acetone (1:1), eluting with acetone by sephadex LH-20 to afford five subfractions (B1–B5). Subfraction B3 was purified by reverse-phase HPLC using acetonitrile–H_2_O (1:1.4) to afford **3** (10.6 mg) and **4** (9.4 mg).

#### 3.3.1. Cherbonnolide A (**1**)

Colorless oil; ${\left[\alpha \right]}_{D}^{25}$ +43 (*c* 1.00, CHCl_3_); IR (neat) *ν*_max_ 3444, 1746, and 1003 cm^−1^; ^13^C and ^1^H NMR data see Table 1; HRMS (ESI-TOF) *m/z*: [M + Na]^+^ Calcd for C_20_H_28_O_4_Na 355.1880; Found 355.1879.

#### 3.3.2. Cherbonnolide B (**2**)

Colorless oil; ${\left[\alpha \right]}_{D}^{25}$ +59 (*c* 1.00, CHCl_3_); IR (neat) *ν*_max_ 3363, 1741, 1678, and 1093 cm^−1^; ^13^C and ^1^H NMR data see Table 1; HRMS (ESI-TOF) *m/z*: [M + Na]^+^ Calcd for C_20_H_28_O_5_Na 371.1829; Found 371.1830.

#### 3.3.3. Cherbonnolide C (**3**)

Colorless oil; ${\left[\alpha \right]}_{D}^{25}$ +26 (*c* 1.00, CHCl_3_); IR (neat) *ν*_max_ 3445, 1749, 1678, and 1094 cm^−1^; ^13^C and ^1^H NMR data see Table 1; HRMS (ESI-TOF) *m/z*: [M + Na]^+^ Calcd for C_20_H_28_O_4_Na 355.1880; Found 355.1882.

#### 3.3.4. Cherbonnolide D (**4**)

White powder; ${\left[\alpha \right]}_{D}^{25}$ +3 (*c* 1.00, CHCl_3_); IR (neat) *ν*_max_ 3445, 1748, and 1096 cm^−1^; ^13^C and ^1^H NMR data see Table 1; HRMS (ESI-TOF) *m/z*: [M + Na]^+^ Calcd for C_20_H_28_O_4_Na 355.1880; Found 355.1883.

#### 3.3.5. Cherbonnolide E (**5**)

Colorless oil; ${\left[\alpha \right]}_{D}^{25}$ +8 (*c* 1.00, CHCl_3_); IR (neat) *ν*_max_ 3389, 1748, 1678 and 1096 cm^−1^; ^13^C and ^1^H NMR data see Table 2; HRMS (ESI-TOF) *m/z*: [M + Na]^+^ Calcd for C_20_H_28_O_5_Na 371.1829; Found 371.1830.

#### 3.3.6. Bischerbolide Peroxide (**6**)

White powder; ${\left[\alpha \right]}_{D}^{25}$ +41 (*c* 1.00, CHCl_3_); IR (neat) *ν*_max_ 3420, 1733, 1232, 1166 and 1040 cm^−1^; ^13^C and ^1^H NMR data see Table 2; HRMS (ESI-TOF) *m/z*: [M + Na]^+^ Calcd for C_40_H_58_O_6_Na 657.4125; Found 657.4124.

#### 3.3.7. Reduction of Cherbonolides B and E (**2** and **5**)

In diethyl ether (5.0 mL), compound **2** (3.2 mg) was added followed by addition of excess amount of triphenylphosphine (2.9 mg) and the mixture was stirred at room temperature for 4 h. The solvent of the solution was evaporated under reduced pressure to afford a residue, which was purified by silica gel column chromatography using *n*-hexane–acetone (3:1) as an eluent to yield **1** (2.9 mg, 95%). Similarly, **5** (2.1 mg) was converted to **4** (1.7 mg) in 85% yield. 

#### 3.3.8. Preparation of (*S*)- and (*R*)- MTPA Esters of **1** and **3**

Compound **1** (1.3 mg) was dissolved in pyridine 100 μL and added (*R*)-(−)-*α*-methoxy-*α*-(trifluoromethyl) phenylacetyl chloride (MTPA chloride) 10 μL. The mixture was permitted to stand at room temperature overnight and the reaction was found to complete by monitoring with normal-phase TLC plate. The solution was dried completely under the vacuum of an oil pump and the residue was purified by a short silica gel column using acetone to *n*-hexane (1:3) to yield the (*S*)-MTPA ester **1a** (0.9 mg, 62.9%). The same procedure was applied to obtain the (*R*)-MTPA ester **1b** (1.0 mg, 69.9%) from the reaction of (*S*)-(+)-*α*-methoxy-*α*-(trifluoromethyl) phenylacetyl chloride with **1** in pyridine. Selective ^1^H NMR (CDCl_3_, 400 MHz) data of **1a**: δ_H_ 4.925 (1H, d, *J* = 10.0 Hz, H-3), 1.769 (3H, s, H_3_-18), 2.821 (1H, dd, *J* = 12.8, 4.8 Hz, H-5a), 2.376 (1H, m, H-5b), 5.107 (1H, d, *J* = 9.6 Hz, H-7), 1.880 (3H, s, H_3_-19), 2.460 (1H, m, H-9a), 1.989 (1H, m, H-9b); selective ^1^H NMR (CDCl_3_, 400 MHz) data of **1b**: δ_H_ 4.905 (1H, d, *J* = 10.0 Hz, H-3), 1.763 (3H, s, H_3_-18), 2.739 (1H, dd, *J* = 12.8, 5.6 Hz, H-5a), 2.267 (1H, m, H-5b), 5.217 (1H, d, *J* = 9.6 Hz, H-7), 1.905 (3H, s, H_3_-19), 2.476 (1H, m, H-9a), 1.998 (1H, m, H-9b).

Preparation of (*S*)- and (*R*)- MTPA esters of **3** used the same reaction and purification procedures as the reduction of **1**, the solution of **3** (1.1 mg) was converted to the (*S*)-MTPA ester **3a** (0.8 mg) in 74% yield and (*R*)-MTPA ester **3b** (0.9 mg) in 80% yield, respectively. Selective ^1^HNMR (C_6_D_6_, 400 MHz) data of **3a**: δ_H_ 1.243 (3H, s, H_3_-18), 2.311 (1H, dd, *J* = 11.2, 2.4 Hz, H-5a), 1.930 (1H, dd, *J* = 11.2, 11.2 Hz, H-5b), 5.065 (1H, d, *J* = 9.2 Hz, H-7), 1.416 (3H, s, H_3_-19); selective ^1^H NMR (C_6_D_6_, 400 MHz) data of **3b**: δ_H_ 1.244 (3H, s, H_3_-18), 2.363 (1H, dd, *J* = 12.0, 3.2 Hz, H-5a), 1.982 (1H, dd, *J* = 12.0, 11.6 Hz, H-5b), 4.9515 (1H, d, *J* = 10.0 Hz, H-7), 1.360 (3H, s, H_3_-19).

### 3.4. In Vitro Anti-Inflammatory Testing

#### 3.4.1. Human Neutrophils

Blood was obtained from elbow vein of healthy adult volunteers (years 20–30). Neutrophils were enriched by dextran sedimentation, Ficoll-Hypaque centrifugation, and hypotonic lysis. Neutrophils were incubated in an ice-cold Ca^2+^-free HBSS buffer (pH 7.4) [42].

#### 3.4.2. Superoxide Anion Generation

Neutrophils (6 × 10^5^ cells mL^−1^) incubated in HBSS with ferricytochrome *c* (0.5 mg mL^−1^) and Ca^2+^ (1 mM) at 37 °C were treated with DMSO (as control) or compound for 5 min. Neutrophils were primed by cytochalasin B (CB, 1 μg mL^−1^) for 3 min before activating fMLF (100 nM) for 10 min (fMLF/CB) [40,49].

#### 3.4.3. Elastase Release

Neutrophils (6 × 10^5^ cells mL^−1^) incubated in HBSS with MeO-Suc-Ala-Ala-Pro-Val-*p*-nitroanilide (100 μM) and Ca^2+^ (1 mM) at 37 °C were treated with DMSO or compound for 5 min. Neutrophils were activated with fMLF (100 nM)/CB (0.5 μg mL^−1^) for 10 min [40].

#### 3.4.4. Statistical Analysis

Data are displayed as the mean ± SEM and comparisons were performed by Student’s *t*-test. A probability value of 0.05 or less was considered to be significant. The software Sigma Plot (version 8.0, Systat Software, San Jose, CA, USA) was used for the statistical analysis.

## 4. Conclusions

Six new cembranoids, cherbonolides A−E (**1**–**5**) and a cembrane dimer (bischerbolide peroxide, **6**), along with isosarcophine (**7**) were isolated from the Formosan soft coral *Sarcophyton cherbonnieri*. Bischerbolide peroxide (**6**) was discovered as the first example of cembranoid dimers possessing a peroxide group as a linking group. Compounds **1**, **3** and **6** showed an anti-inflammatory activity through their inhibitory effects on the generation of superoxide anion in fMLF/CB-induced human neutrophils. Moreover, peroxide **6** was also shown to exhibit stronger activity in inhibiting the elastase release which supported its anti-inflammatory activity.

## Figures and Tables

**Table 1:** ^1^H and ^13^C NMR chemical shifts for compounds **1**–**4**.

Position	1		2		3		4	
	δ_H_, m (*J* in Hz) *^a^*	δ_C_ *^b^*, type	δ_H_, m (*J* in Hz) *^a^*	δ_C_ *^b^*, type	δ_H_, m (*J* in Hz) *^c^*	δ_C_ *^d^*, type	δ_H_, m (*J* in Hz) *^c^*	δ_C_ *^d^*, type
1		160.7, C		160.6, C		160.6, C		159.8, C
2	5.44, dd (10.0, 1.6)	77.8, CH	5.43, dd (10.0, 1.6)	77.8, CH	4.91, dd (10.0, 1.6)	78.4, CH	4.98, d (10.4)	77.8, CH
3	4.90, d (10.0)	122.2, CH	4.91, d (10.0)	122.7, CH	4.55, d (10.0)	123.9, CH	4.45, d (10.4)	123.5, CH
4		141.6, C		140.8, C		140.2, C		139.4, C
5*α*	2.20, m	47.9, CH_2_	2.18, dd (12.4, 10.8)	42.6, CH_2_	2.42, dd (12.0, 3.2)	49.1, CH_2_	1.98, m	49.3, CH_2_
5*β*	2.76, dd (12.8, 5.2)		2.87, dd (12.4, 4.4)		189, m		2.25, dd (12.8, 3.6)	
6	4.70, ddd (10.4, 10.4, 5.2)	65.2, CH	4.97, ddd (10.8, 9.2, 4.4)	78.3, CH	3.84, dd (9.2, 9.2)	69.6, CH	4.21, ddd (11.2, 9.2, 3.6)	64.8, CH
7	5.20, d (10.4)	128.1, CH	5.05, d (9.2)	123.1, CH	5.09, d (9.2)	131.6, CH	4.84, d (9.2)	131.2, CH
8		139.8, C		144.2, C		138.4, C		139.4, C
9*α*	2.03, m	36.8, CH_2_	2.07, m	36.9, CH_2_	2.21, ddd (13.6, 13.6, 2.4)	28.2, CH_2_	1.58, m	28.5, CH_2_
9*β*	2.38, m		2.42, m		1.63, m		2.30, dd (13.2, 4.8)	
10*α*	1.29, m	23.5, CH_2_	1.35, m	23.6, CH_2_	1.16, m	23.9, CH_2_	1.60, m	22.7, CH_2_
10*β*	2.51, m		2.17, m		1.84, m		1.28, m	
11	2.42, dd (10.8, 2.8)	61.4, CH	2.43, m	61.4, CH	2.24, dd (10.4, 2.4)	58.9, CH	1.98, m	62.6, CH
12		60.8, C		60.8, C		59.7, CH		60.9, C
13*α*	2.03, m	36.9, CH_2_	2.02, m	37.0, CH_2_	1.49, m	35.5, CH_2_	1.61, m	37.1, CH_2_
13*β*	1.06, m		1.07, m		0.98, m		0.65, m	
14*α*	2.49, m	23.7, CH_2_	2.52, m	23.7, CH_2_	1.58, m	22.2, CH_2_	2.08, m	23.1, CH_2_
14*β*	2.01, m		2.03, m		1.58, m		1.65, m	
15		123.8, C		123.8, C		123.8, C		123.7, C
16		174.4, C		174.4, C		173.9, C		174.3, C
17	1.86, s	8.8, CH_3_	1.86, s	8.7, CH_3_	1.64, s	8.8, CH_3_	1.63, s	8.8, CH_3_
18	1.70, s	15.9, CH_3_	1.72, s	15.9, CH_3_	1.31, s	18.1, CH_3_	1.28, s	16.9, CH_3_
19	1.86, s	14.9, CH_3_	1.89, s	15.3, CH_3_	1.45, s	22.2, CH_3_	1.32, s	21.8, CH_3_
20	1.33, s	15.8, CH_3_	1.33, s	15.8, CH_3_	1.00, s	17.1, CH_3_	0.99, s	16.4, CH_3_
6-OOH			7.99, br s					

*^a^* Spectrum recorded at 400 MHz in CDCl_3_. *^b^* Spectrum recorded at 100 MHz in CDCl_3_. *^c^* Spectrum recorded at 400 MHz in C_6_D_6_. *^d^* Spectrum recorded at 100 MHz in C_6_D_6_.

**Table 2:** ^1^H and ^13^C NMR chemical shifts for compounds **5** and **6**.

Position	5		6				
	δ_H_, m (*J* in Hz) *^a^*	δ_C_ *^b^*, type	δ_H_, m (*J* in Hz) *^c^*	δ_C_ *^d^*, type		δ_H_, m (*J* in Hz) *^c^*	δ_C_ *^d^*, type
1		160.4, C		141.4, C	1′		141.5, C
2	4.95, d (10.0)	78.4, CH	5.28, d (10.0)	82.7, CH	2′	5.50, d (10.0)	81.9, CH
3	4.42, d (10.0)	124.6, CH	5.06, d (10.0)	126.4, CH	3′	4.92, d (10.0)	125.1, CH
4		139.2, C		140.2, C	4′		141.0, C
5*α*	1.95, m	44.6, CH_2_	2.21, m	38.5, CH_2_	5′*α*	2.21, m	38.8, CH_2_
5*β*	2.47, br d (11.0)		2.32, m		5′*β*	2.31, m	
6	4.58, ddd (11.0, 9.5, 2.5)	78.9, CH		24.2, CH_2_	6′		24.2, CH_2_
6*α*			2.07, m		6′*α*	2.07, m	
6*β*			2.42, m		6′*β*	2.42, m	
7	4.78, d (9.5)	126.6, CH	4.98, dd (9.2, 9.2)	125.6, CH	7′	4.95, dd (9.2, 9.2)	125.5, CH
8		143.8, C		133.1, C	8′		133.3, C
9*α*	1.65, m	29.8, CH_2_	1.96, m	36.6, CH_2_	9′*α*	1.96, m	36.6, CH_2_
9*β*	2.52, dd (14.0, 4.5)		2.27, m		9′*β*	2.27, m	
10*α*	1.28, m	23.4, CH_2_	1.22, m	23.6, CH_2_	10′*α*	1.22, m	23.7, CH_2_
10*β*	1.62, m		2.04, m		10′*β*	2.04, m	
11	1.97, m	63.3, CH	2.51, m	62.1, CH	11′	2.51, m	62.2, CH
12		61.5, C		61.2, CH	12′		61.3, C
13*α*	1.59, m	37.6, CH_2_	1.83, m	37.3, CH_2_	13′*α*	1.83, m	37.4, CH_2_
13*β*	0.64, m		0.95, m		13′*β*	0.95, m	
14*α*	2.07, m	23.7, CH_2_	2.33, m	22.6, CH_2_	14′*α*	2.33, m	22.7, CH_2_
14*β*	1.61, m		1.81, m		14′*β*	1.81, m	
15		124.3, C		124.9, C	15′		124.9, C
16		174.3, C	6.13, br s	114.3, C	16′	6.17, d (3.6)	114.4, CH
17	1.63, s	9.4, CH_3_	1.72, s	10.2, CH_3_	17′	1.73, s	10.2, CH_3_
18	1.29, s	17.3, CH_3_	1.58, s	14.6, CH_3_	18′	1.59, s	14.6, CH_3_
19	1.34, s	22.5, CH_3_	1.65, s	14.7, CH_3_	19′	1.65, s	14.7, CH_3_
20	0.98, s	16.9, CH_3_	1.27, s	15.7, CH_3_	20′	1.27, s	15.7, CH_3_
6-OOH	7.25, br s						

*^a^* Spectrum recorded at 500 MHz in C_6_D_6_. *^b^* Spectrum recorded at 125 MHz in C_6_D_6_. *^c^* Spectrum recorded at 400 MHz in CDCl_3_. *^d^* Spectrum recorded at 100 MHz in CDCl_3_.

**Table 3:** Selected ^1^H and ^13^C NMR data comparison with **6**, (2*S*,11*R*,12*R*)-isosarcophytoxide (**8**) and (2*R*,11*R*,12*R*)-isosarcophytoxide (**9**).

Position	6	8 *^a^*	9 *^a^*
H-11	δ_H_ 2.51 (H-11, H-11′)	δ_H_ 2.50	δ_H_ 2.75
C-11	δ_C_ 62.1 (C-11)	δ_C_ 62.3	δ_C_ 61.2
	δ_C_ 62.2 (C-11′)		
C-12	δ_C_ 61.2 (C-12)	δ_C_ 61.4	δ_C_ 60.7
	δ_C_ 61.3 (C-12′)		
C-13	δ_C_ 37.3 (C-13)	δ_C_ 37.4	δ_C_ 35.4
	δ_C_ 37.4 (C-13′)		
C-14	δ_C_ 22.6 (C-14)	δ_C_ 22.5	δ_C_ 20.4
	δ_C_ 22.7 (C-14′)		
H_3_-18	δ_H_ 1.58 (H_3_-18)	δ_H_ 1.58	δ_H_ 1.70
	δ_H_ 1.59 (H_3_-18′)		
H_3_-20	δ_H_ 1.27 (H_3_-20, H_3_-20′)	δ_H_ 1.28	δ_H_ 1.18
C-20	δ_C_ 15.7 (C-20, C-20′)	δ_C_ 15.7	δ_C_ 17.7

*^a^* The selected ^1^H and ^13^C data were cited from ref. [36,37].

**Table 4:** Inhibitory effects of compounds **1**–**7** on superoxide anion generation and elastase release in fMLF/CB-induced human neutrophils.

Compounds	Superoxide Anion		Elastase Release
	IC_50_ (μM) ^a^	Inh ^b^ %	Inh ^b^ %
**1**	>30	32.1 ± 4.3 **	37.6 ± 5.0 **
**2**	>30	4.0 ± 6.7	23.5 ± 6.6 *
**3**	>30	44.5 ± 4.6 ***	35.6 ± 6.2 **
**4**	>30	6.4 ± 4.2	27.6 ± 6.4 **
**5**	>30	2.6 ± 6.2	30.5 ± 4.6 **
**6**	26.2 ± 1.0	64.6 ± 0.8 ***	42.4 ± 5.1 **
**7**	>30	3.5 ± 5.3	20.7 ± 4.1 **
Idelalisib	0.07 ± 0.01	102.8 ± 2.2 ***	99.6 ± 4.2

^a^ Concentration necessary for 50% inhibition (IC_50_). ^b^ Percentage of inhibition (Inh %) at 30 μM. Data are presented as mean ± S.E.M. (*n* = 3–4); * *p* < 0.05, ** *p* < 0.01, *** *p* < 0.001 compared with the control value.

## Supplementary Materials

HRESIMS, ^1^H, ^13^C, DEPT, HMQC, COSY, HMBC and NOESY spectra of new compounds **1**–**6**, and ^1^H NMR spectra of (+)-sarcophytoxide and sarcophytonin after different treatments are available online at http://www.mdpi.com/1660-3397/16/8/276/s1. Figure S1: HRESIMS spectrum of **1**, Figure S2: ^1^H NMR spectrum of **1** in CDCl_3_, Figure S3: ^13^C NMR spectrum of **1** in CDCl_3_, Figure S4: HSQC spectrum of **1** in CDCl_3_, Figure S5: ^1^H-^1^HCOSY spectrum of **1** in CDCl_3_, Figure S6: HMBC spectrum of **1** in CDCl_3_, Figure S7: NOESY spectrum of **1** in CDCl_3_, Figure S8: HRESIMS spectrum of **2**, Figure S9: ^1^H NMR spectrum of **2** in CDCl_3_, Figure S10: ^13^C NMR spectrum of **2** in CDCl_3_, Figure S11: HSQC spectrum of **2** in CDCl_3_, Figure S12: ^1^H-^1^HCOSY spectrum of **2** in CDCl_3_, Figure S13: HMBC spectrum of **2** in CDCl_3_, Figure S14: NOESY spectrum of **2** in CDCl_3_, Figure S15: HRESIMS spectrum of **3**, Figure S16: ^1^H NMR spectrum of **3** in C_6_D_6_, Figure S17: ^13^C NMR spectrum of **1** in C_6_D_6_, Figure S18: HSQC spectrum of **1** in C_6_D_6_, Figure S19: ^1^H-^1^HCOSY spectrum of **3** in C_6_D_6_, Figure S20: HMBC spectrum of **3** in C_6_D_6_, Figure S21: NOESY spectrum of **3** in C_6_D_6_, Figure S22: HRESIMS spectrum of **4**, Figure S23: ^1^H NMR spectrum of **4** in C_6_D_6_, Figure S24: ^13^C NMR spectrum of **4** in C_6_D_6_, Figure S25: HSQC spectrum of **4** in C_6_D_6_, Figure S26: ^1^H-^1^HCOSY spectrum of **4** in C_6_D_6_, Figure S27: HMBC spectrum of **4** in C_6_D_6_, Figure S28: NOESY spectrum of **4** in C_6_D_6_, Figure S29: HRESIMS spectrum of **5**, Figure S30: ^1^H NMR spectrum of **5** in C_6_D_6_, Figure S31: ^13^C NMR spectrum of **5** in C_6_D_6_, Figure S32: HSQC spectrum of **5** in C_6_D_6_, Figure S33: ^1^H-^1^HCOSY spectrum of **5** in C_6_D_6_, Figure S34: HMBC spectrum of **5** in C_6_D_6_, Figure S35: NOESY spectrum of **5** in C_6_D_6_, Figure S36: HRESIMS spectrum of **6**, Figure S37: ESIMS spectrum of **6**, S38: ^1^H NMR spectrum of **6** in CDCl_3_, Figure S39: ^13^C NMR spectrum of **6** in CDCl_3_, Figure S40: HSQC spectrum of **6** in CDCl_3_, Figure S41: ^1^H-^1^HCOSY spectrum of **6** in CDCl_3_, Figure S42: HMBC spectrum of **6** in CDCl_3_, Figure S43: NOESY spectrum of **6** in CDCl_3_, Figure S44: ^1^H NMR spectrum of (+)-sarcophytoxide in CDCl_3_ before treatment with acetone and silica gel under air, Figure S45. ^1^H NMR spectrum of (+)-sarcophytoxide in CDCl_3_ after treatment with acetone and silica gel under air, Figure S46. ^1^H NMR spectrum of sarcophytonin A in CDCl_3_ before treatment with acetone and silica gel under air, Figure S47. ^1^H NMR spectrum of sarcophytonin A in CDCl_3_ after treatment with acetone and silica gel under air.
