# Supplementary material for: New Cembranoids and a Biscembranoid Peroxide from the Soft Coral Sarcophyton cherbonnieri
Source: Mar Drugs. 2018 Aug 6;16(8):276. doi: 10.3390/md16080276 (PMC6117711; doi:10.3390/md16080276)
Supplement: Supplementary file 1 [file marinedrugs-16-00276-s001.pdf]

Supplementary information

New Cembranoids and a Biscembranoid Peroxide from the Soft Coral *Sarcophyton cherbonnieri*

Chia-Chi Peng [1], Chiung-Yao Huang [1], Atallah F. Ahmed [2,3], Tsong-Long Hwang [4,5,6], Chang-Feng Dai [7], Jyh-Horng Sheu[1,8,9,10,*]

[1] Department of Marine Biotechnology and Resources, National Sun Yat-sen University, Kaohsiung 804, Taiwan; m045020008@mail.nsysu.edu.tw (C.-C. P); huangcy@mail.nsysu.edu.tw (C.-Y.H.)
[2] Department of Pharmacognosy, College of Pharmacy, King Saud University, Riyadh 11451, Saudi Arabia; afahmed@ksu.edu.sa
[3] Department of Pharmacognosy, Faculty of Pharmacy, Mansoura University, Mansoura 35516, Egypt
[4] Graduate Institute of Natural Products, College of Medicine, Chang Gung University, Taoyuan 333, Taiwan
[5] Research Center for Industry of Human Ecology and Graduate Institute of Health Industry Technology, Chang Gung University of Science and Technology, Taoyuan 333, Taiwan
[6] Department of Anesthesiology, Chang Gung Memorial Hospital, Taoyuan 333, Taiwan; htl@mail.cgu.edu.tw
[7] Institute of Oceanography, National Taiwan University, Taipei 112, Taiwan; corallab@ntu.edu.tw
[8] Graduate Institute of Natural Products, Kaohsiung Medical University, Kaohsiung 807, Taiwan
[9] Frontier Center for Ocean Science and Technology, National Sun Yat-sen University, Kaohsiung 804, Taiwan
[10] Department of Medical Research, China Medical University Hospital, China Medical University, Taichung 404, Taiwan

* Correspondence: sheu@mail.nsysu.edu.tw

Table of Contents

Figure S1	HRESIMS spectrum of **1**	S4
Figure S2	^{1}H NMR spectrum of **1** in $CDCl_3$	S5
Figure S3	^{13}C NMR spectrum of **1** in $CDCl_3$	S6
Figure S4	HSQC spectrum of **1** in $CDCl_3$	S7
Figure S5	^{1}H-^{1}H COSY spectrum of **1** in $CDCl_3$	S8
Figure S6	HMBC spectrum of **1** in $CDCl_3$	S9
Figure S7	NOESY spectrum of **1** in $CDCl_3$	S10
Figure S8	HRESIMS spectrum of **2**	S11
Figure S9	^{1}H NMR spectrum of **2** in $CDCl_3$	S12
Figure S10	^{13}C NMR spectrum of **2** in $CDCl_3$	S13
Figure S11	HSQC spectrum of **2** in $CDCl_3$ S14	S14
Figure S12	^{1}H-^{1}H COSY spectrum of **2** in $CDCl_3$	S15
Figure S13	HMBC spectrum of **2** in $CDCl_3$	S16
Figure S14	NOESY spectrum of **2** in $CDCl_3$	S17
Figure S15	HRESIMS spectrum of **3**	S18
Figure S16	^{1}H NMR spectrum of **3** in C_6D_6	S19
Figure S17	^{13}C NMR spectrum of **3** in C_6D_6	S20
Figure S18	HSQC spectrum of **3** in C_6D_6 S21	S21
Figure S19	^{1}H-^{1}H COSY spectrum of **3** in C_6D_6	S22
Figure S20	HMBC spectrum of **3** in C_6D_6	S23
Figure S21	NOESY spectrum of **3** in C_6D_6	S24
Figure S22	HRESIMS spectrum of **4**	S25
Figure S23	^{1}H NMR spectrum of **4** in C_6D_6	S26
Figure S24	^{13}C NMR spectrum of **4** in C_6D_6	S27
Figure S25	HSQC spectrum of **4** in C_6D_6	S28
Figure S26	^{1}H-^{1}H COSY spectrum of **4** in C_6D_6	S29
Figure S27	HMBC spectrum of **4** in C_6D_6	S30
Figure S28	NOESY spectrum of **4** in C_6D_6	S31
Figure S29	HRESIMS spectrum of **5**	S32
Figure S30	^{1}H NMR spectrum of **5** in C_6D_6	S33
Figure S31	^{13}C NMR spectrum of **5** in C_6D_6	S34
Figure S32	HSQC spectrum of **5** in C_6D_6	S35
Figure S33	^{1}H-^{1}H COSY spectrum of **5** in C_6D_6	S36
Figure S34	HMBC spectrum of **5** in C_6D_6	S37
Figure S35	NOESY spectrum of **5** in C_6D_6	S38
Figure S36	HRESIMS spectrum of **6**	S39
Figure S37	ESIMS spectrum of **6**	S40
Figure S38	^{1}H NMR spectrum of **6** in $CDCl_3$	S41

Figure S39	^{13}C NMR spectrum of **6** in $CDCl_3$	S42
Figure S40	HSQC spectrum of **6** in $CDCl_3$	S43
Figure S41	^{1}H-^{1}H COSY spectrum of **6** in $CDCl_3$	S44
Figure S42	HMBC spectrum of **6** in $CDCl_3$	S45
Figure S43	NOESY spectrum of **6** in $CDCl_3$	S46
Figure S44	^{1}H NMR spectrum of (+)-sarcophytoxide in $CDCl_3$ before treatment with acetone and silica gel under air	S47
Figure S45	^{1}H NMR spectrum of (+)-sarcophytoxide in $CDCl_3$ after treatment with acetone and silica gel under air	S48
Figure S46	^{1}H NMR spectrum of sarcophytonin A in $CDCl_3$ before treatment with acetone and silica gel under air	S49
Figure S47	^{1}H NMR spectrum of sarcophytonin A in $CDCl_3$ after treatment with acetone and silica gel under air	S50

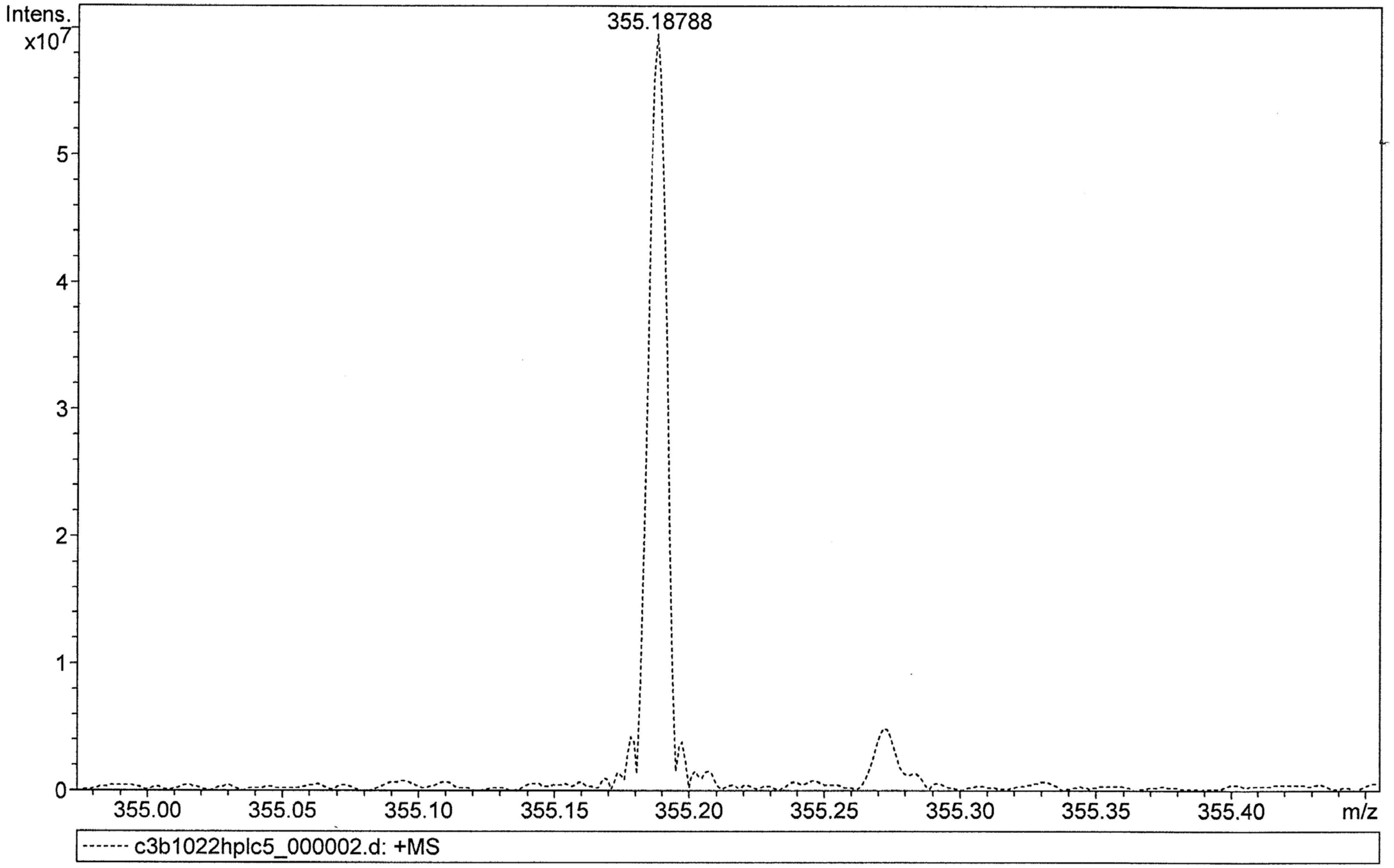

Meas. m/z	#	Formula	Score	m/z	err [mDa]	err [ppm]	mSigma	rdb	e⁻ Conf	N-Rule
355.18788	1	C 20 H 28 Na O 4	100.00	355.18798	0.10	0.29	5.9	6.5	even	ok

Figure S1. HRESIMS spectrum of **1**

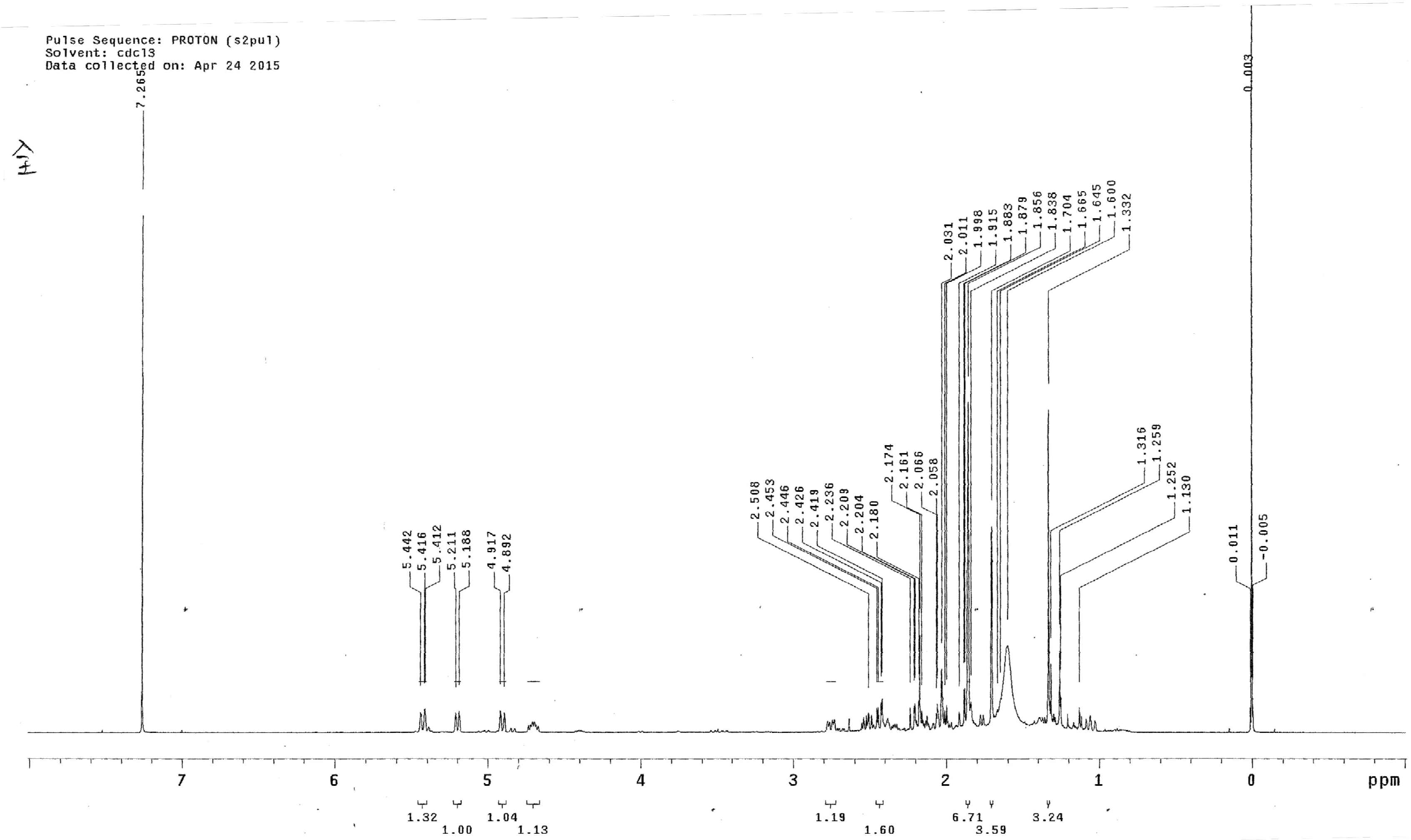

Figure S2. ^{1}H NMR spectrum of **1** in $CDCl_3$

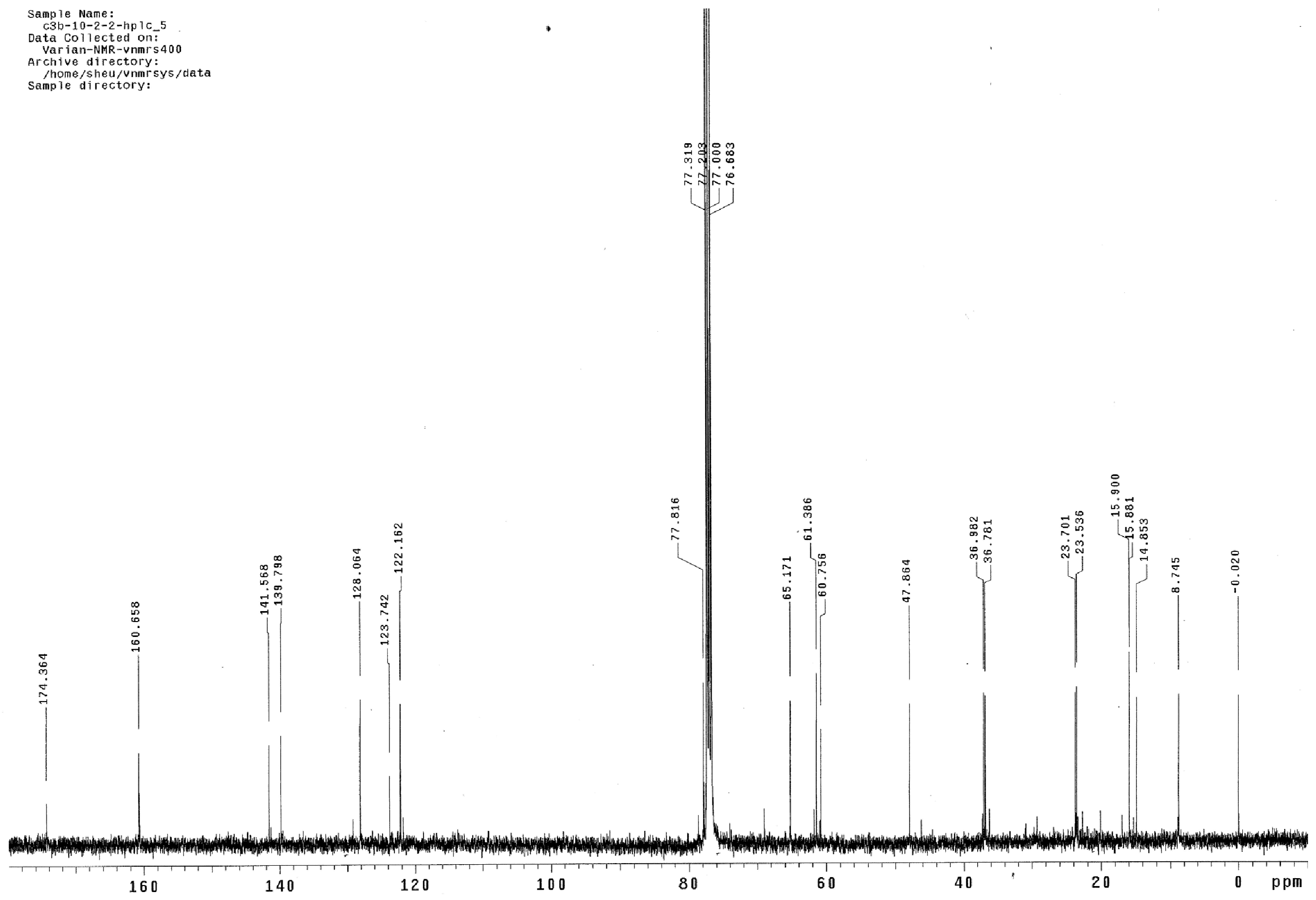

Figure S3. ^{13}C NMR spectrum of **1** in $CDCl_3$

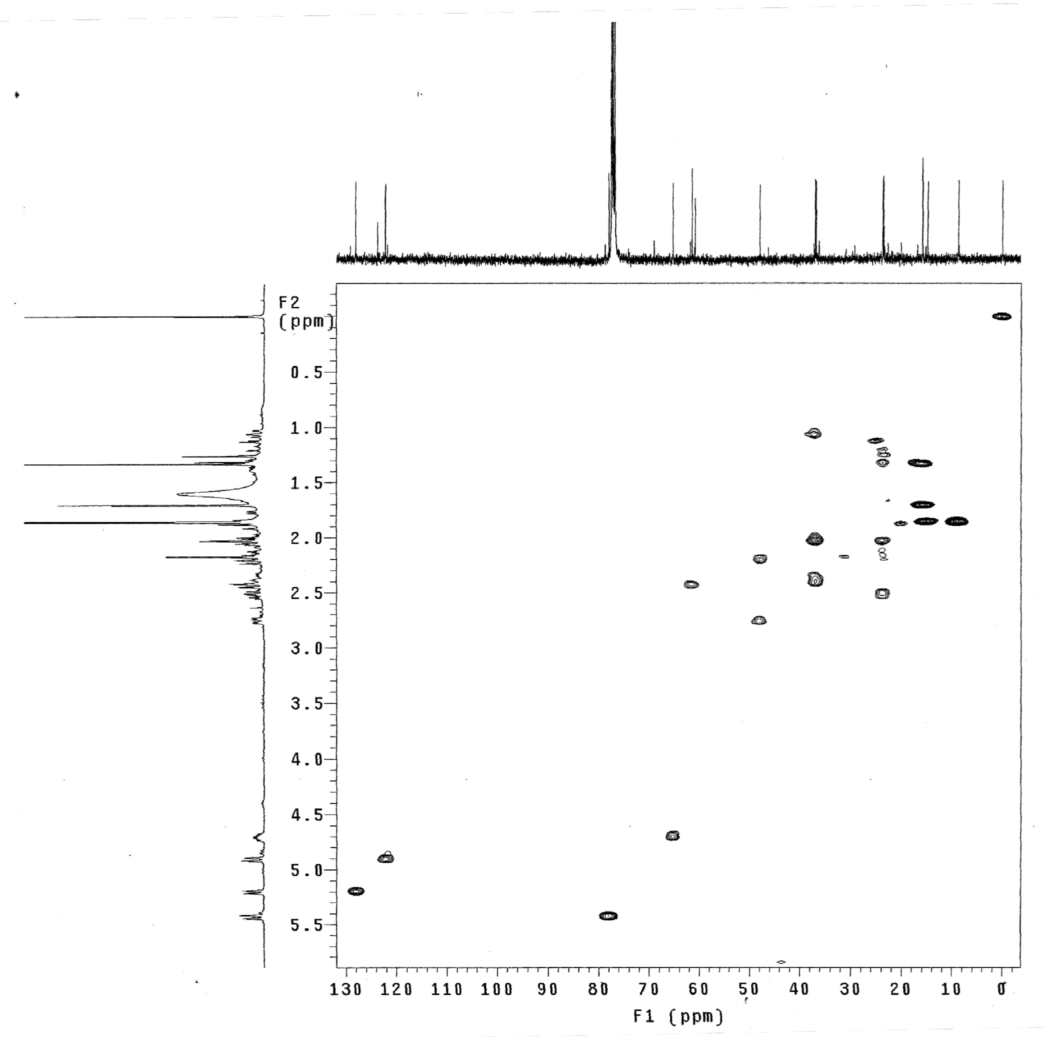

Figure S4. HSQC spectrum of **1** in $CDCl_3$

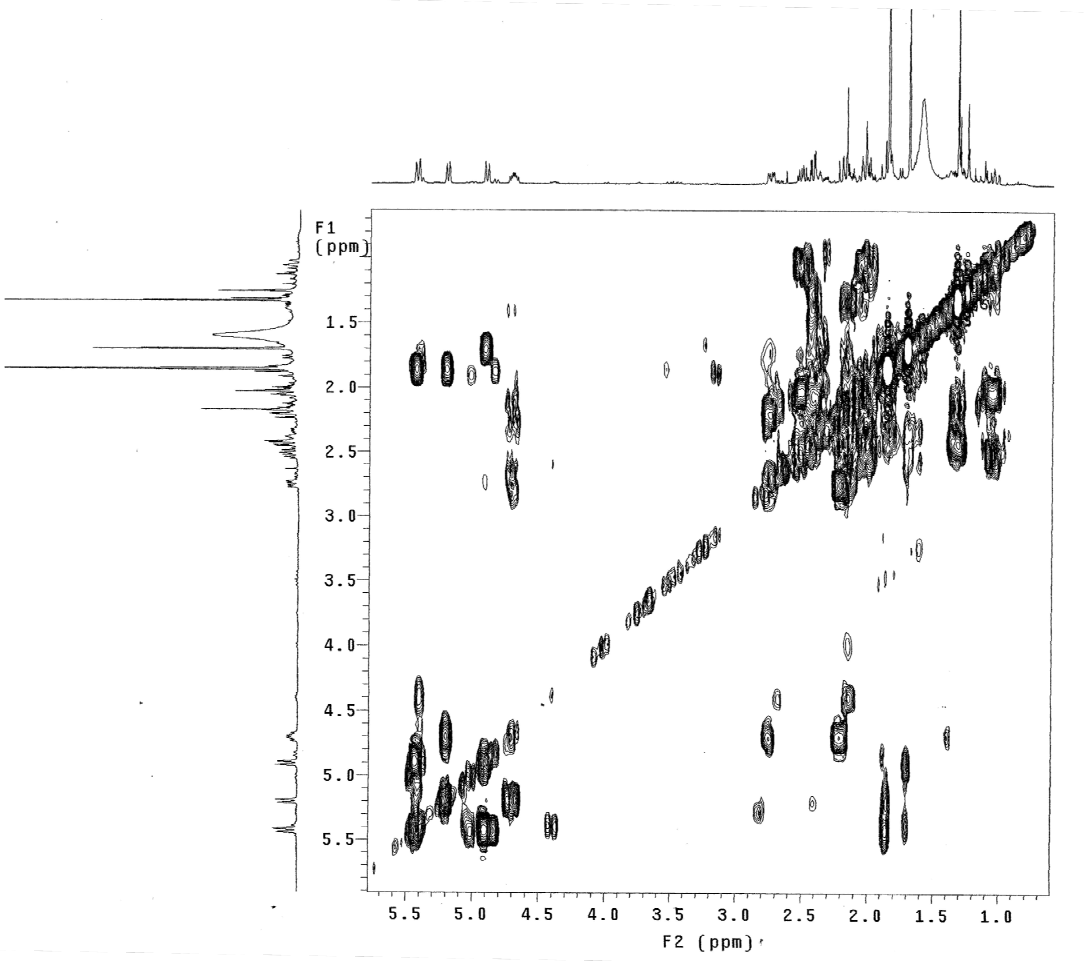

Figure S5. ^{1}H-^{1}H COSY spectrum of **1** in $CDCl_3$

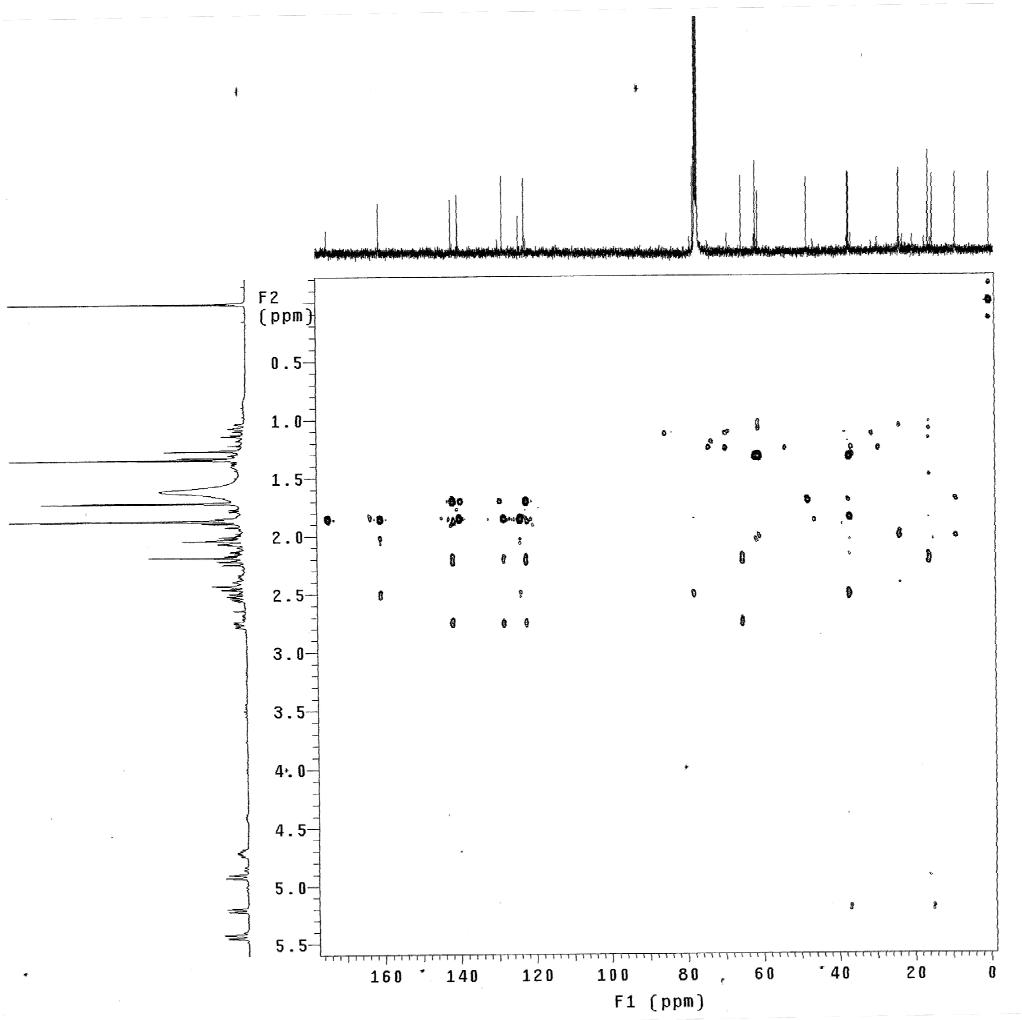

Figure S6. HMBC spectrum of **1** in $CDCl_3$

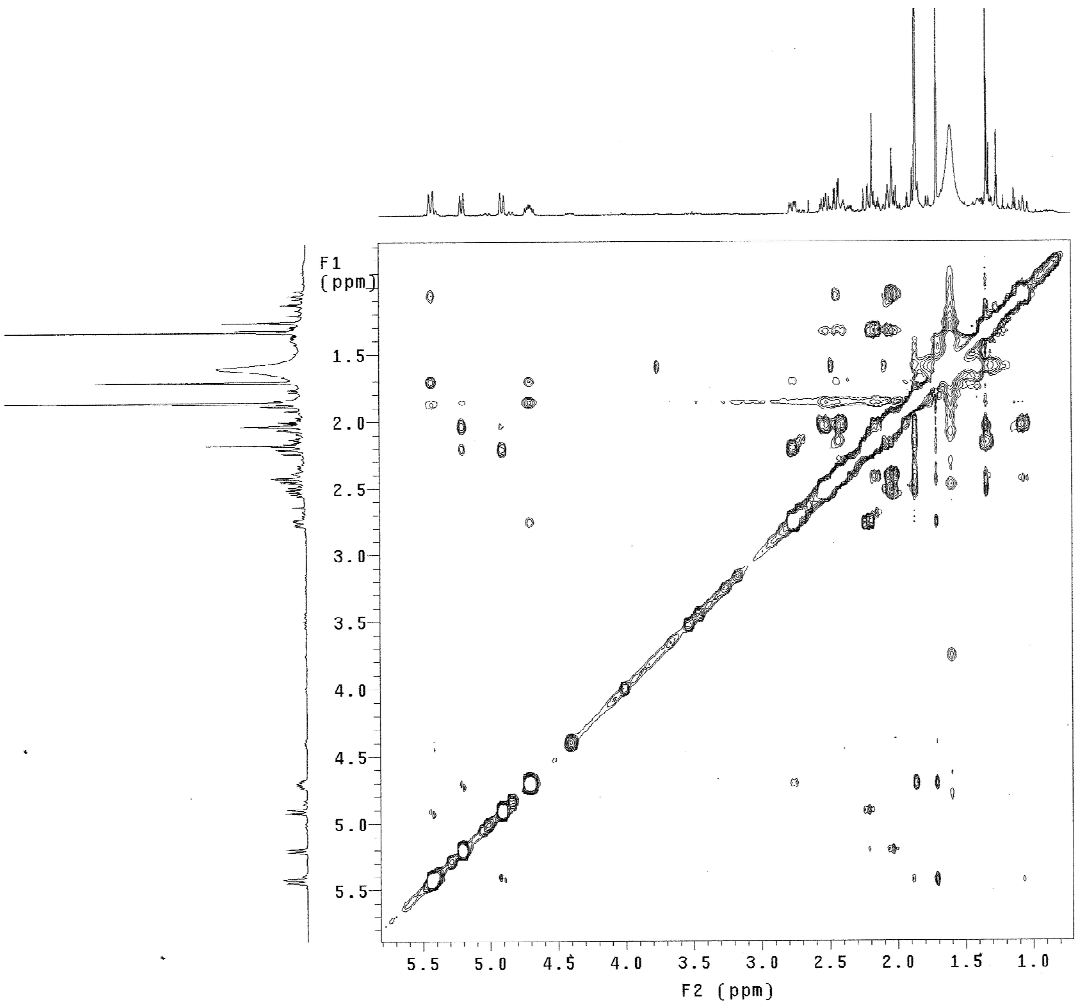

Figure S7. NOESY spectrum of **1** in $CDCl_3$

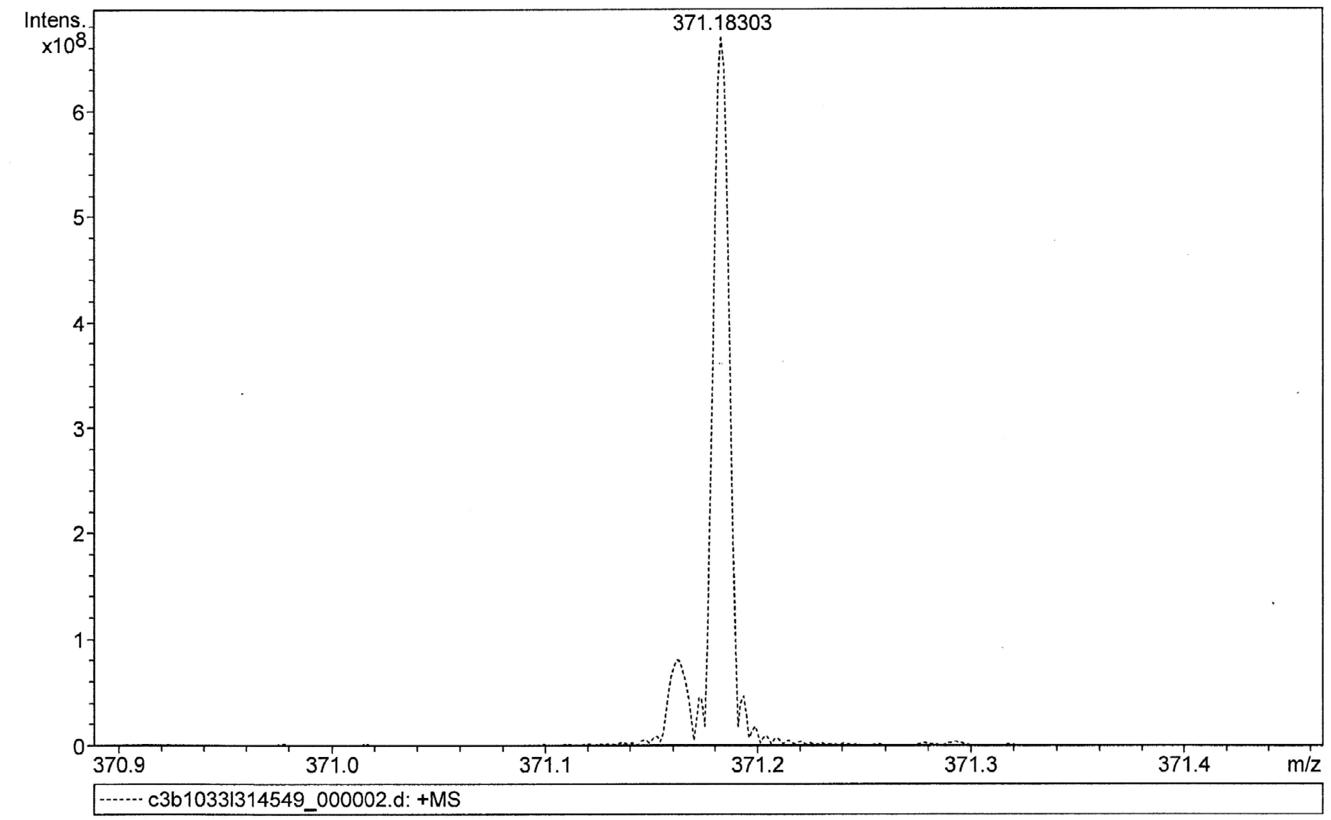

Meas. m/z	#	Formula	Score	m/z	err [mDa]	err [ppm]	mSigma	rdb	e⁻ Conf	N-Rule
371.18303	1	C 20 H 28 Na O 5	100.00	371.18290	-0.14	-0.37	6.2	6.5	even	ok

Figure S8. HRESIMS spectrum of **2**

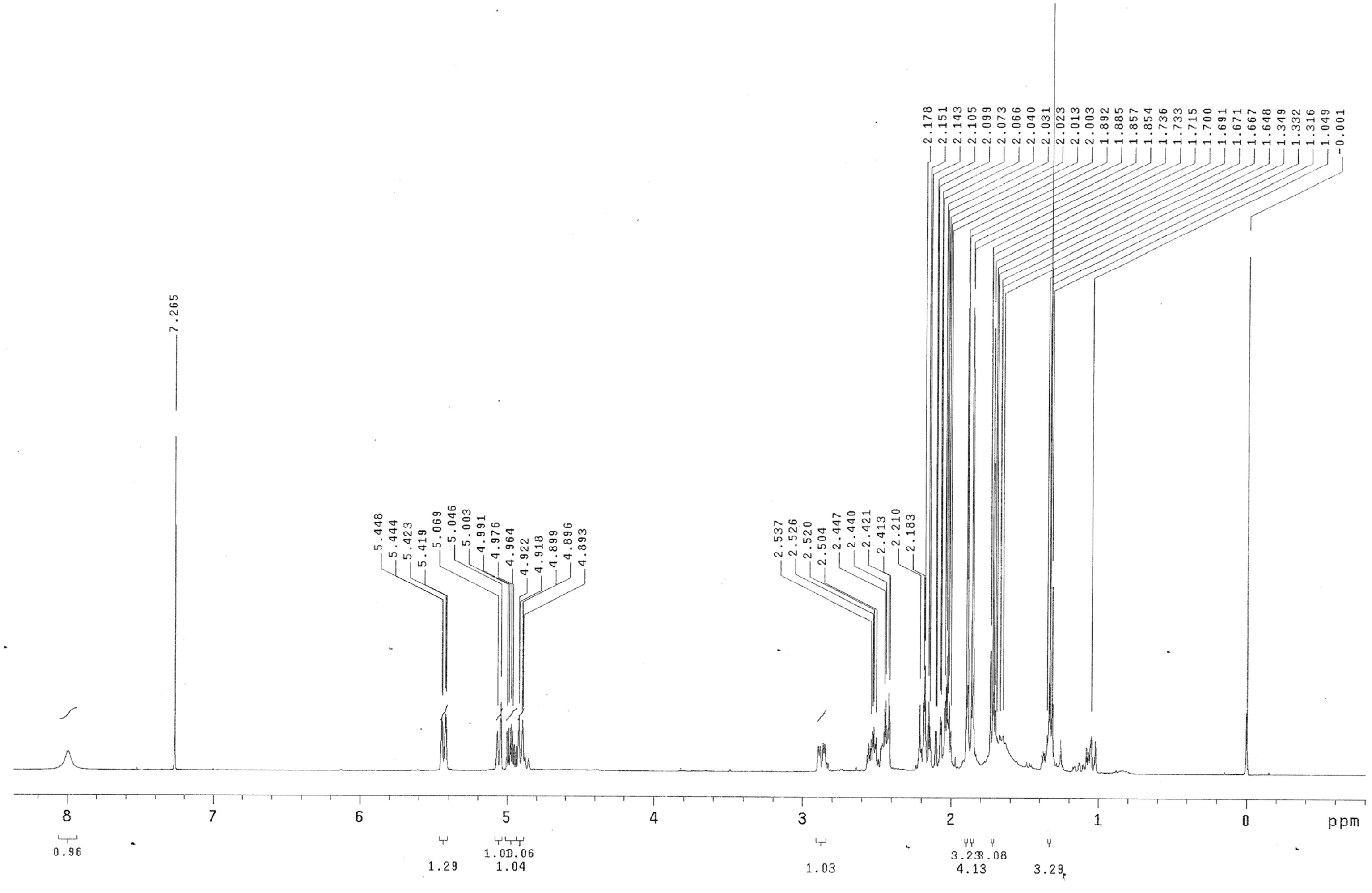

Figure S9. ^{1}H NMR spectrum of **2** in $CDCl_3$

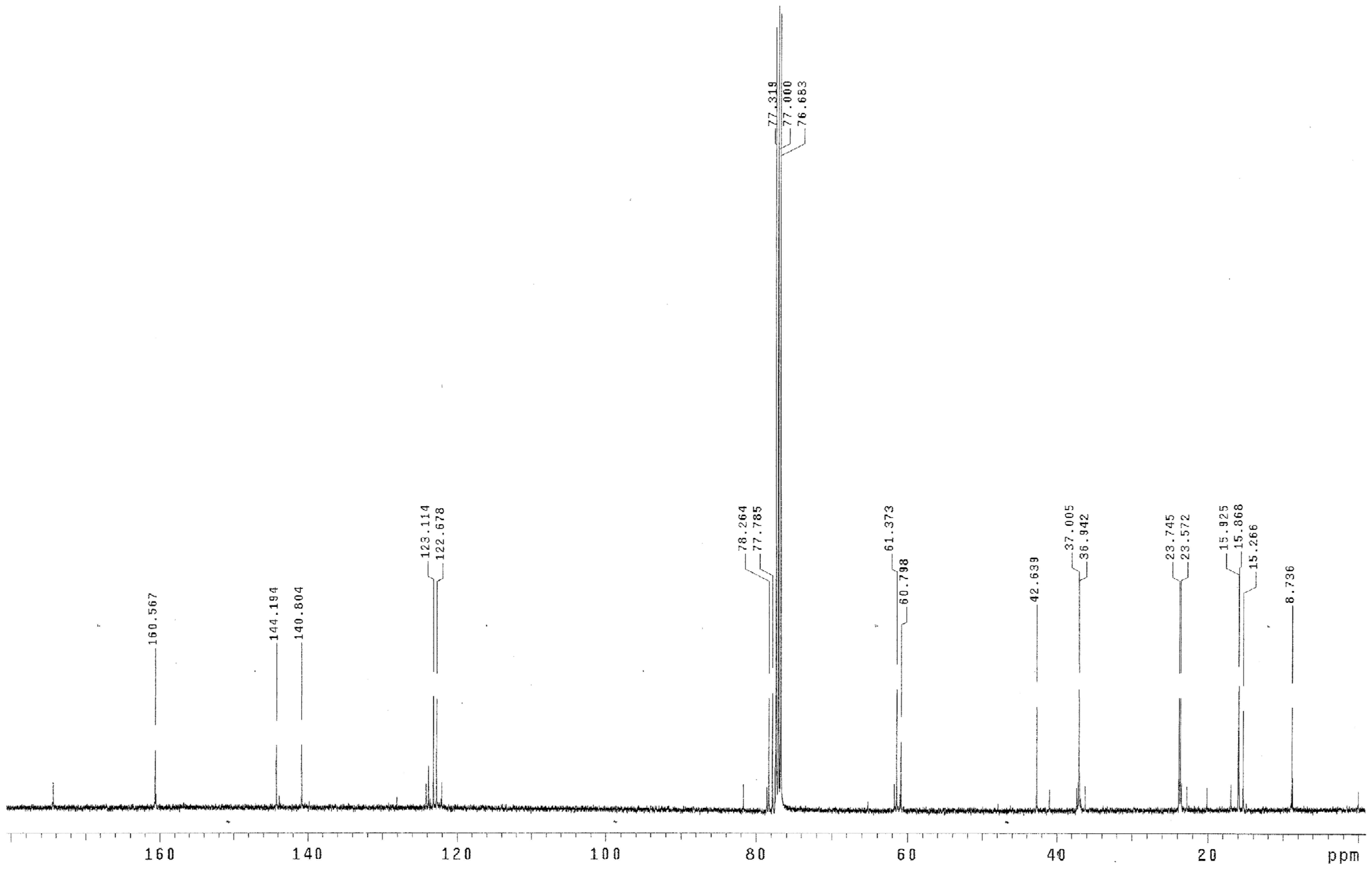

Figure S10. ^{13}C NMR spectrum of **2** in $CDCl_3$

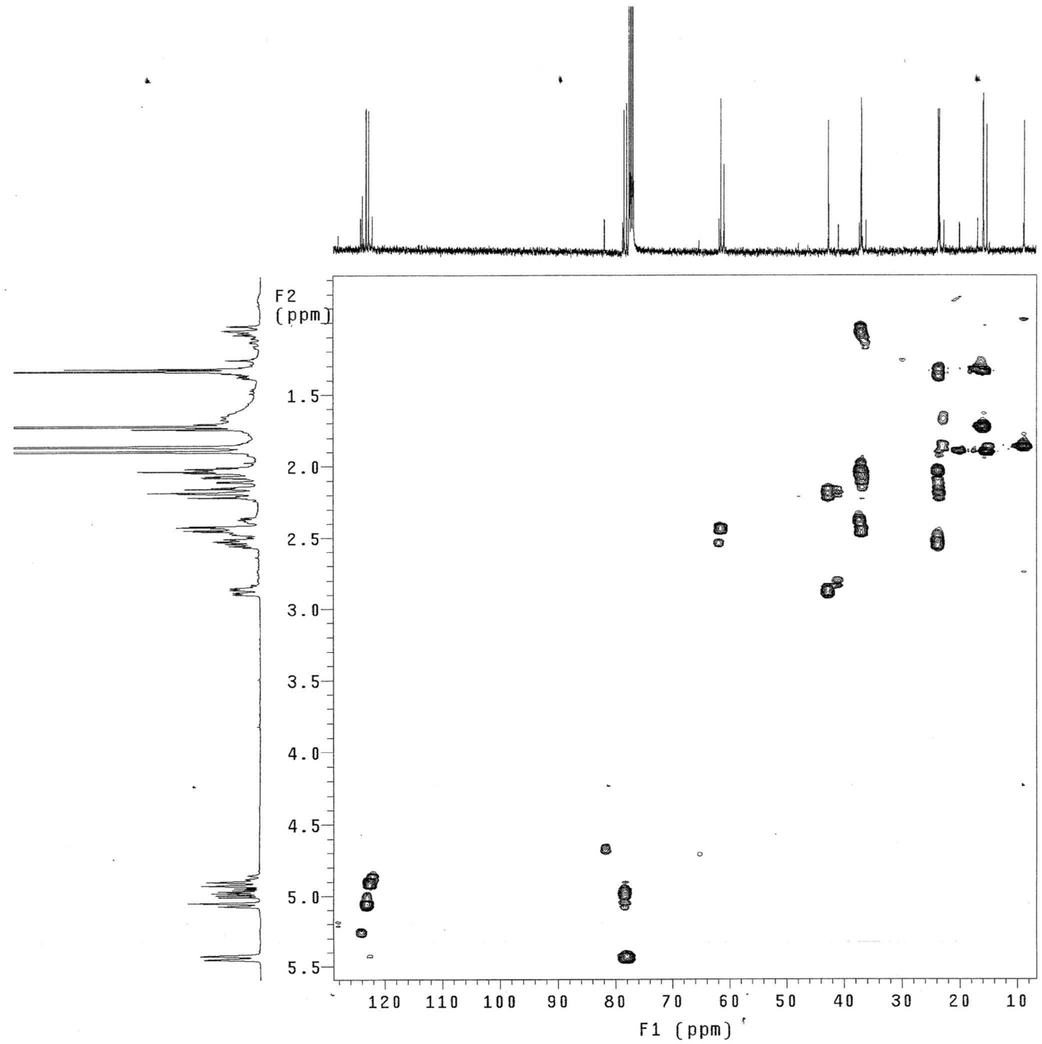

Figure S11. HSQC spectrum of **2** in $CDCl_3$

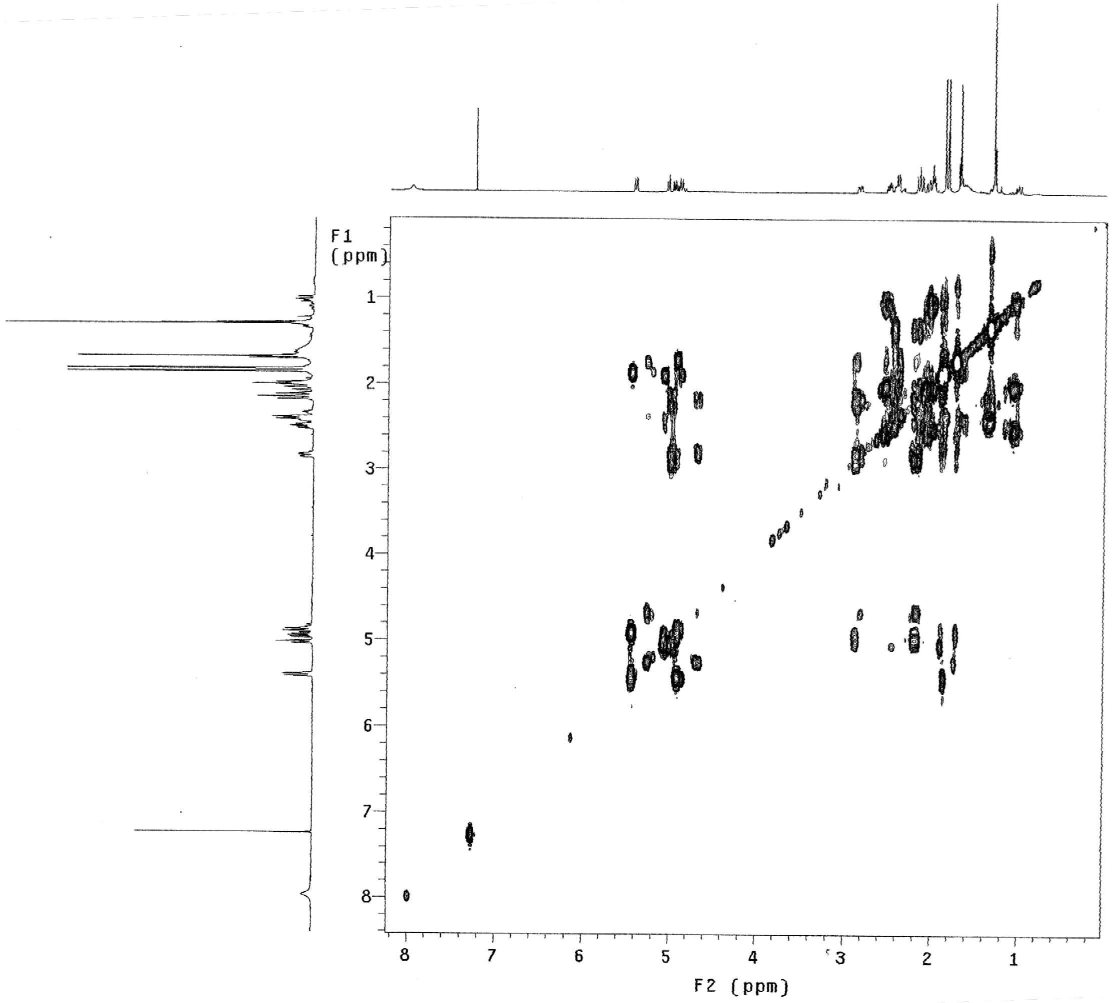

Figure S12. ^{1}H-^{1}H COSY spectrum of **2** in $CDCl_3$

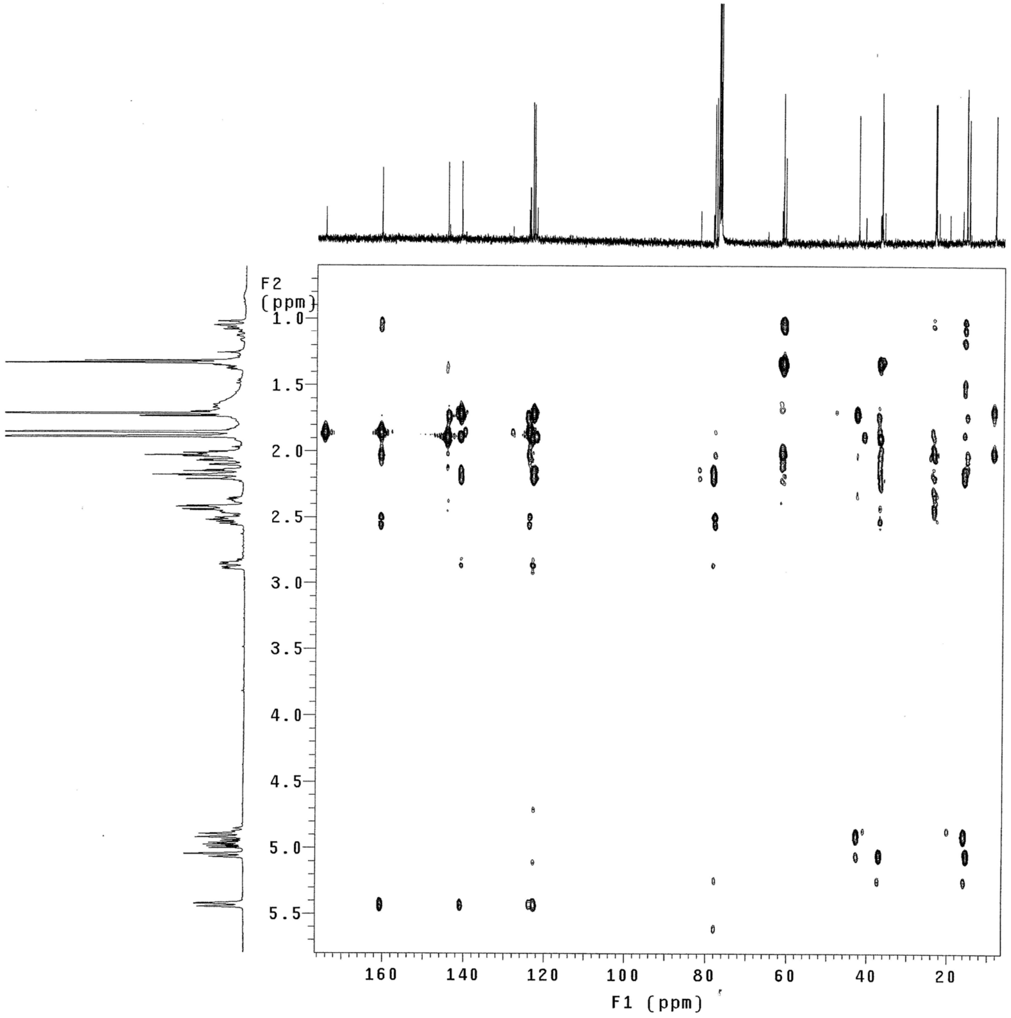

Figure S13. HMBC spectrum of **2** in C_6D_6

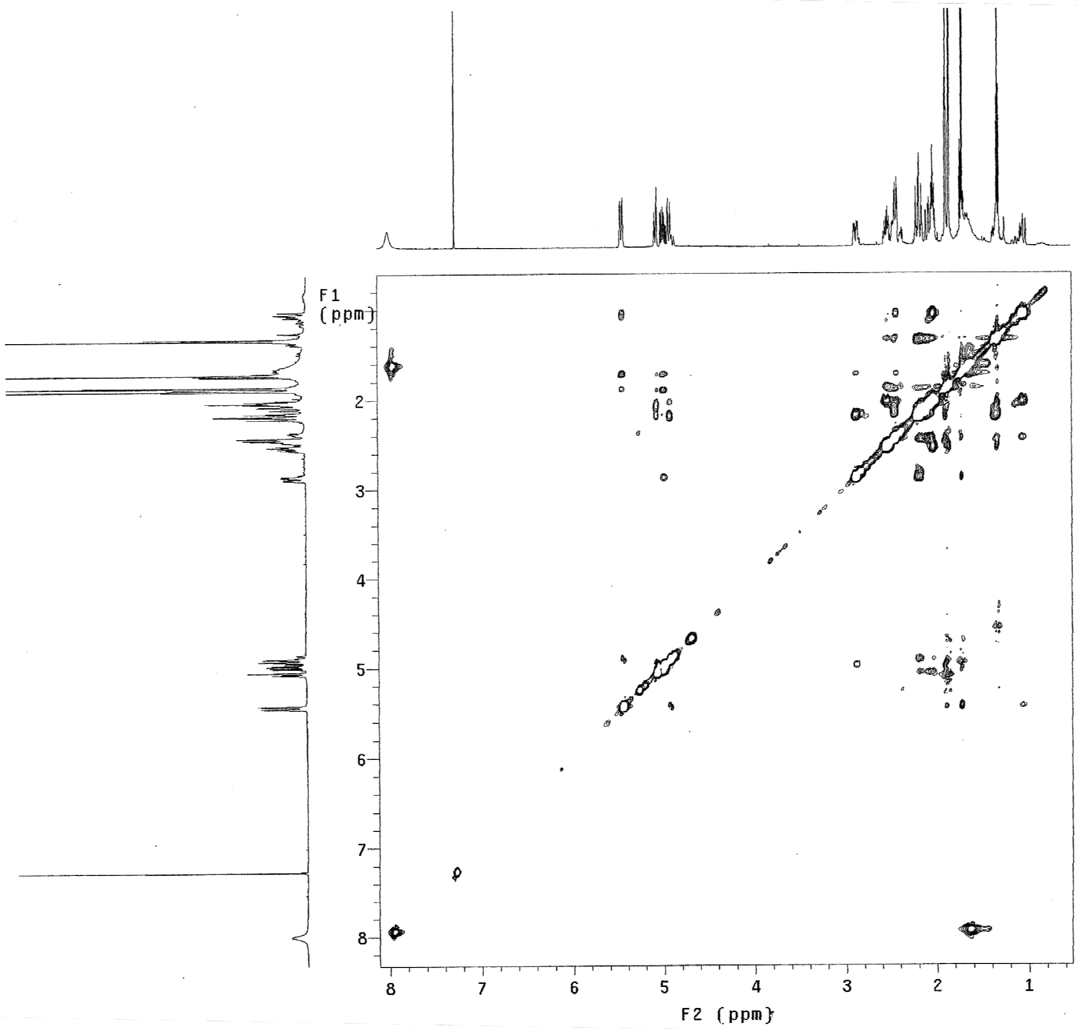

Figure S14. NOESY spectrum of **2** in C_6D_6

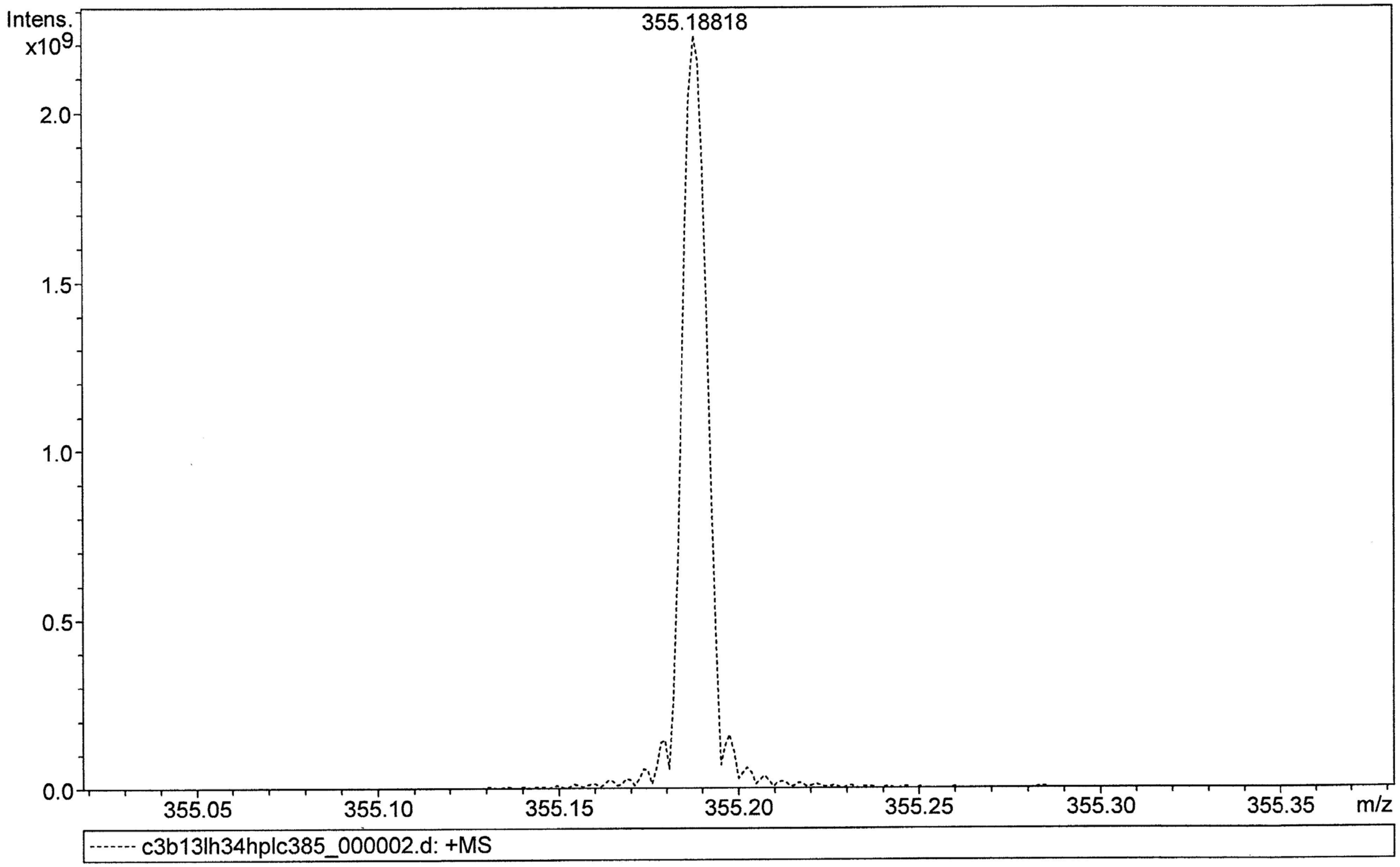

Meas. m/z	#	Formula	Score	m/z	err [mDa]	err [ppm]	mSigma	rdb	e⁻ Conf	N-Rule
355.18818	1	C 20 H 28 Na O 4	100.00	355.18798	-0.20	-0.57	6.3	6.5	even	ok

Figure S15. HRESIMS spectrum of **3**

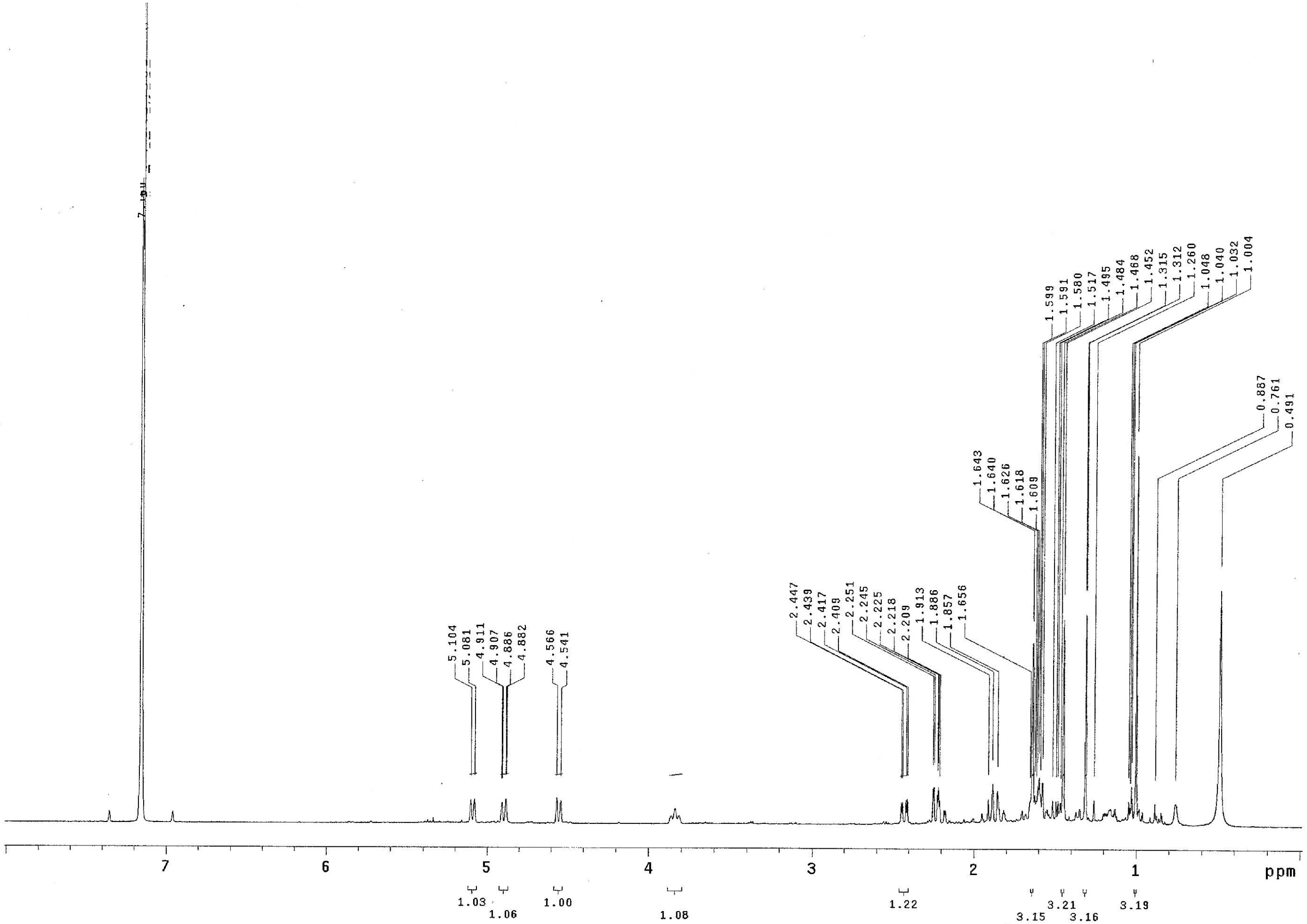

Figure S16. ^{1}H NMR spectrum of **3** in C_6D_6

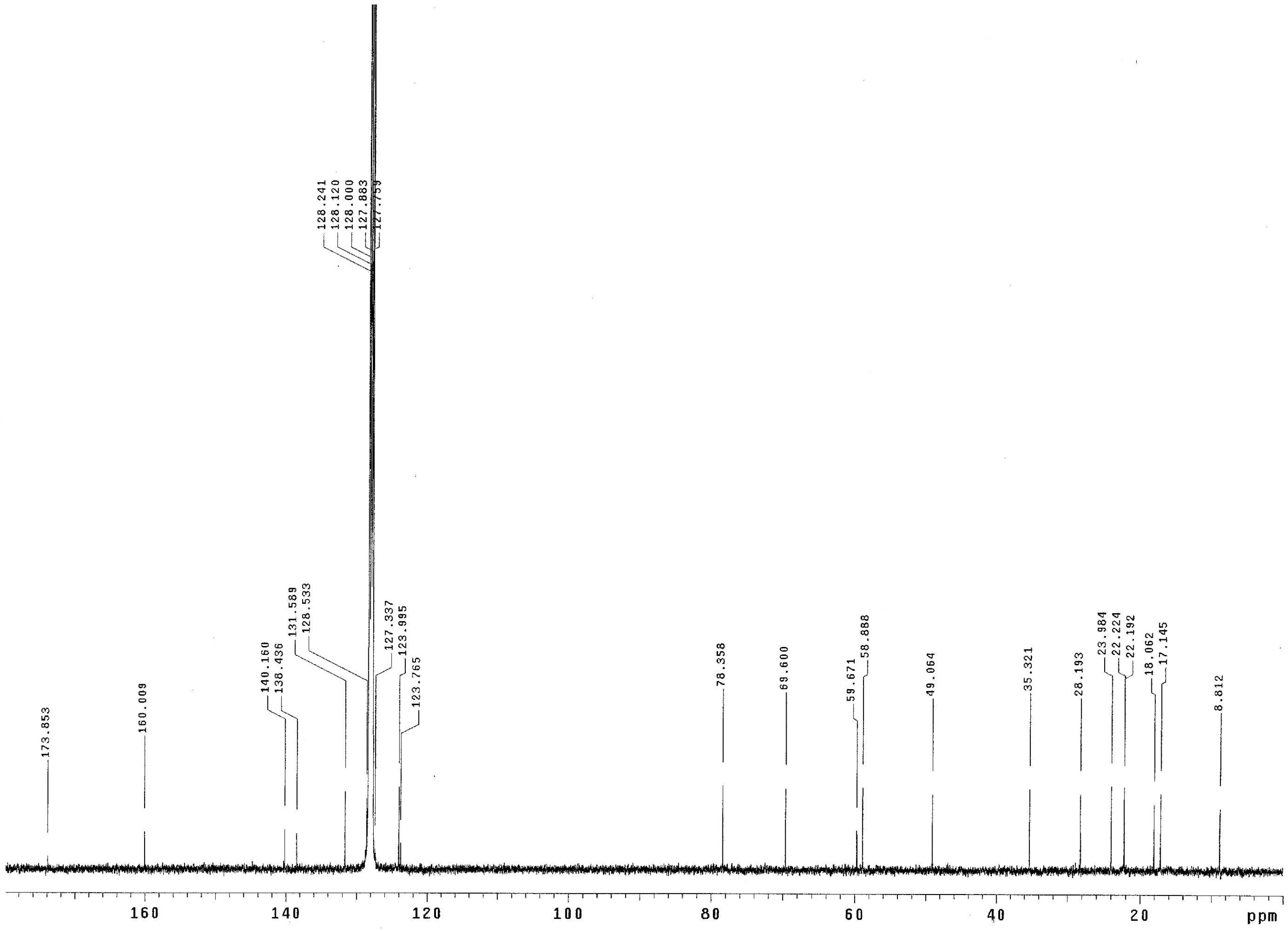

Figure S17. ^{13}C NMR spectrum of **3** in C_6D_6

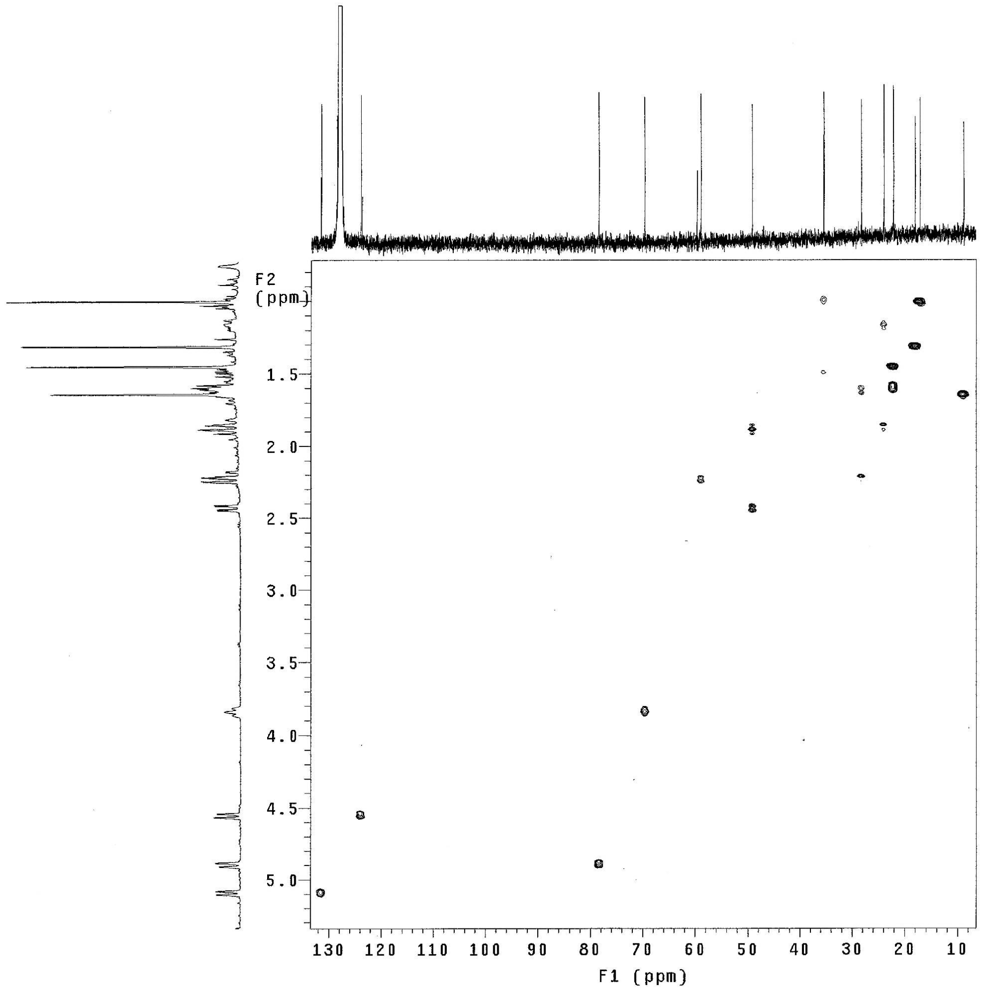

Figure S18. HSQC spectrum of **3** in C_6D_6

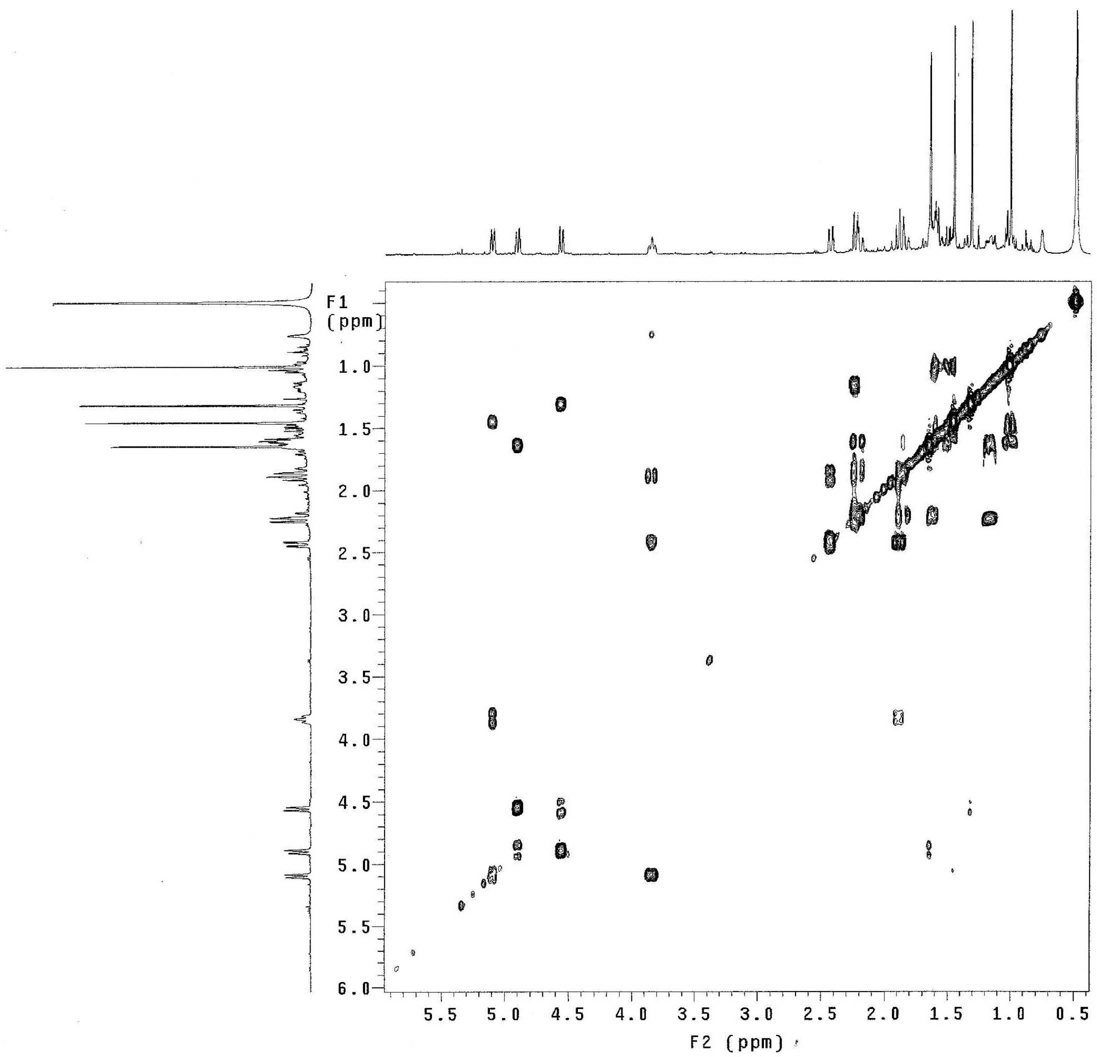

Figure S19. ^1H-^1H COSY spectrum of **3** in C_6D_6

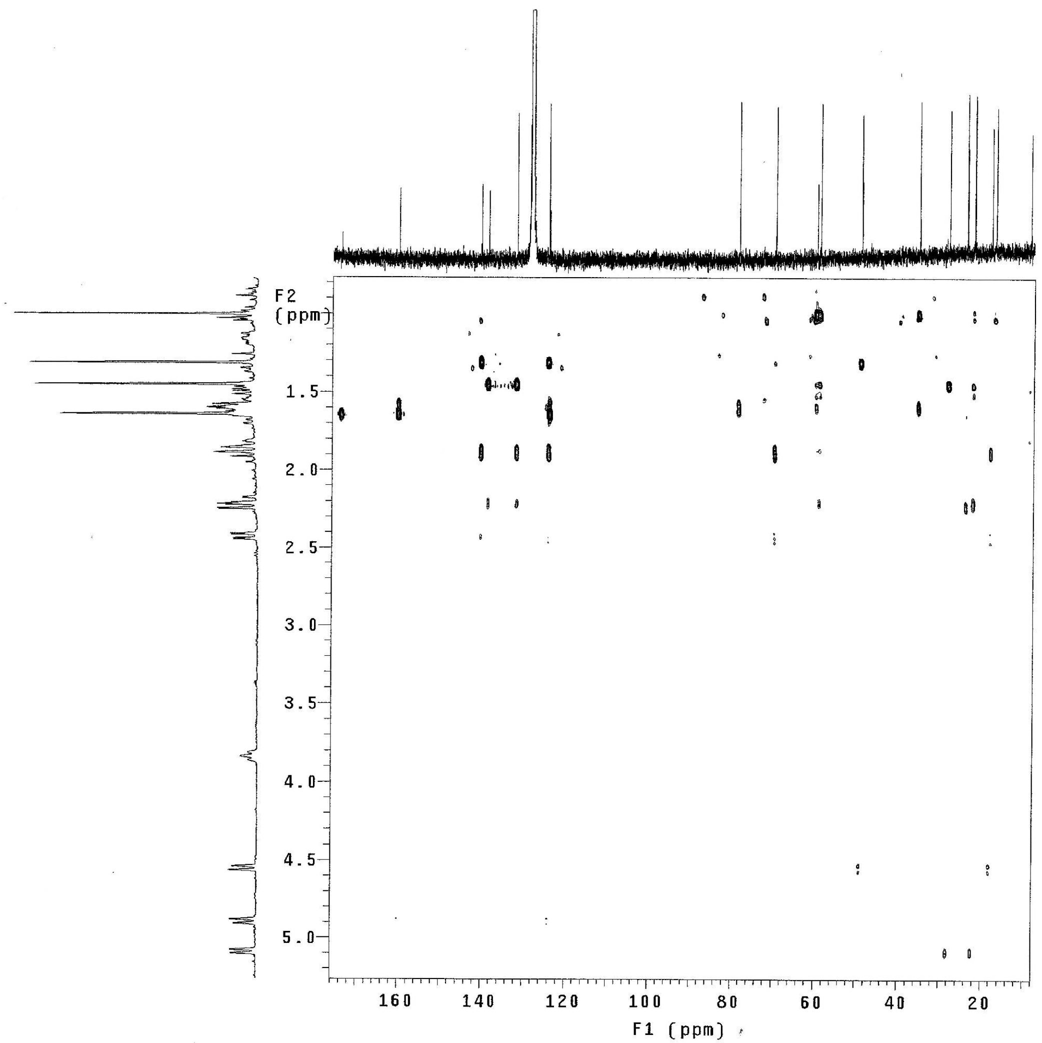

Figure S20. HMBC spectrum of **3** in C_6D_6

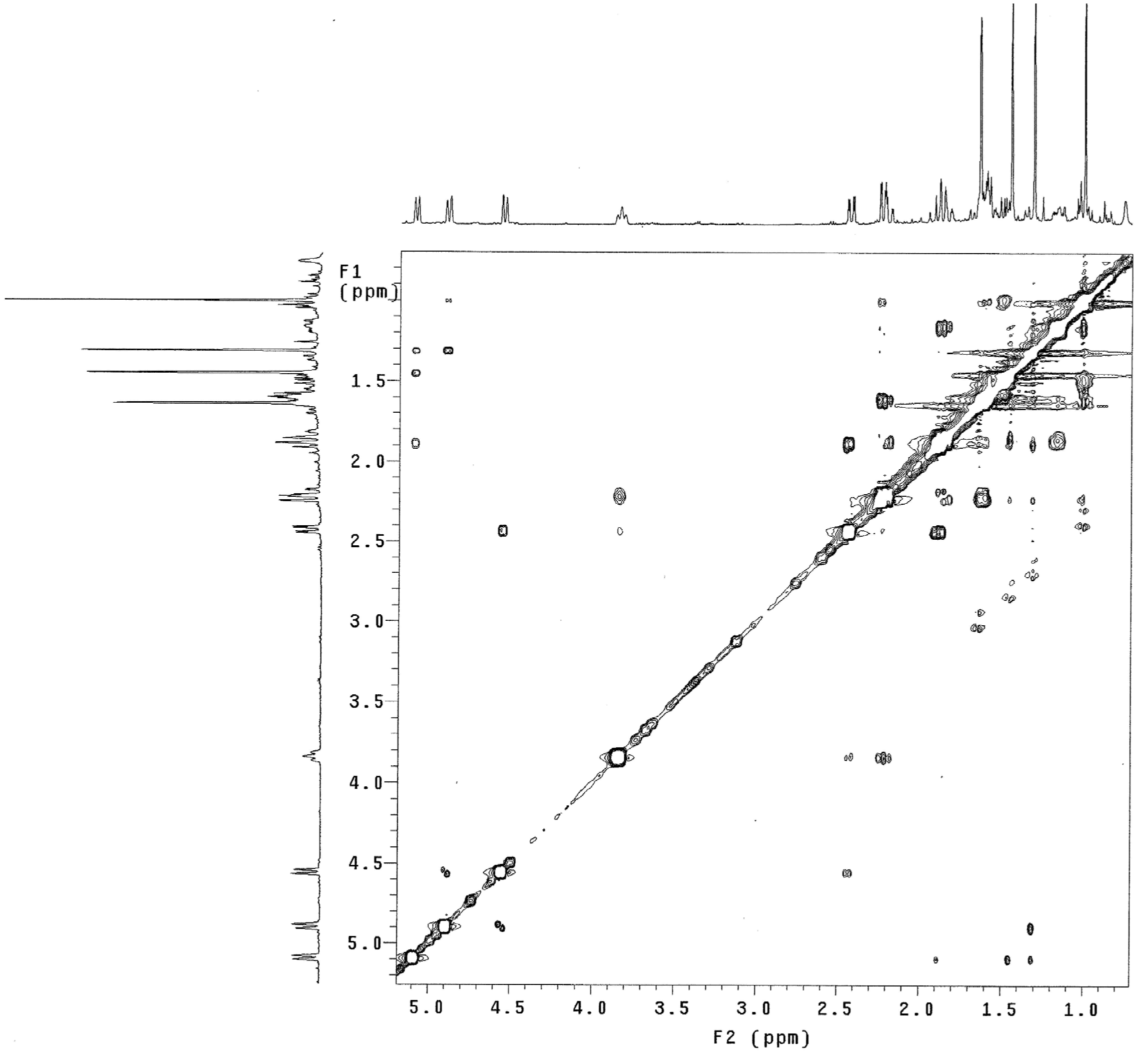

Figure S21. NOESY spectrum of **3** in C_6D_6

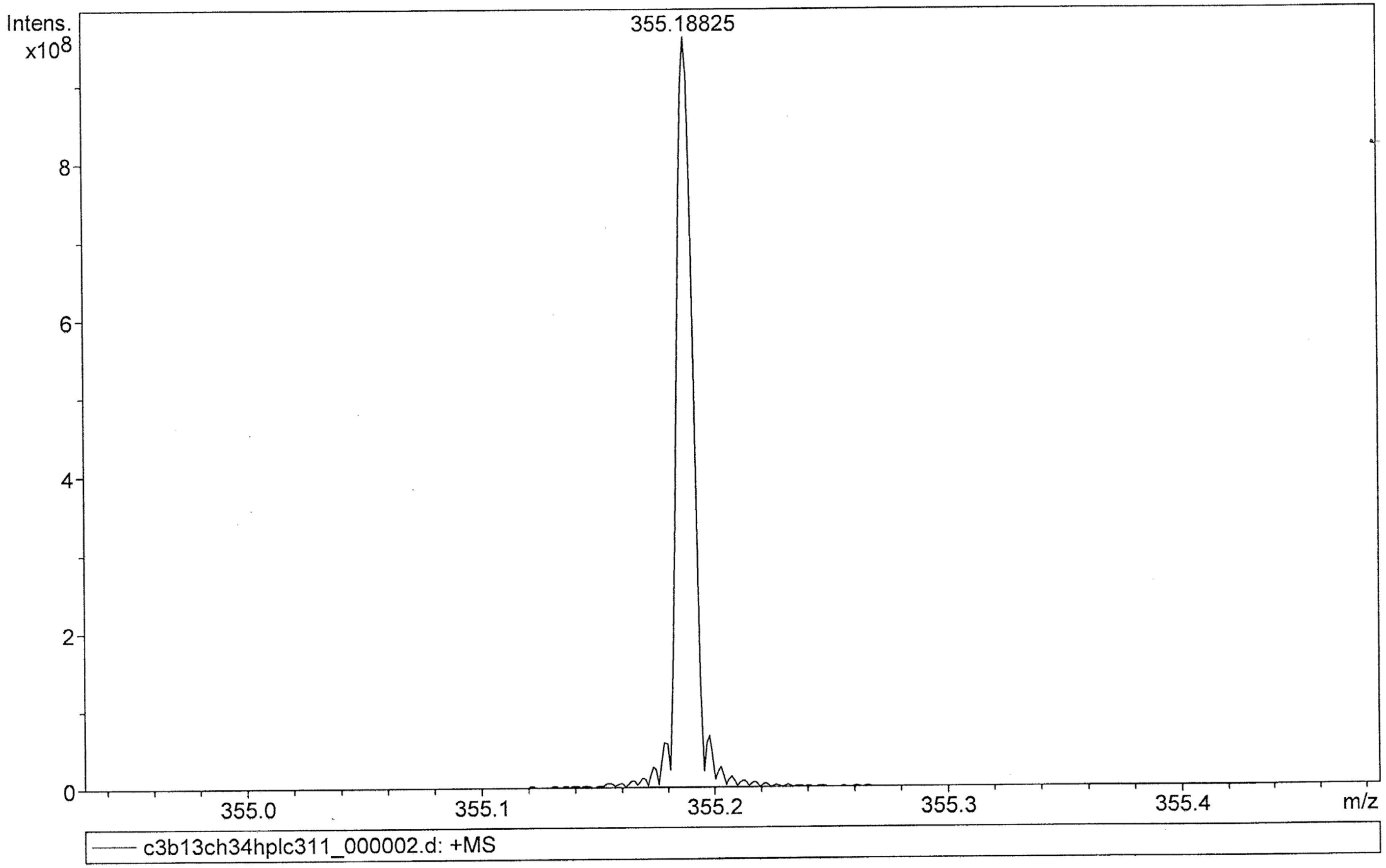

Meas. m/z	#	Formula	Score	m/z	err [mDa]	err [ppm]	mSigma	rdb	e⁻ Conf	N-Rule
355.18825	1	C 20 H 28 Na O 4	100.00	355.18798	-0.27	-0.75	13.1	6.5	even	ok

Figure S22. HREIMS spectrum of **4**

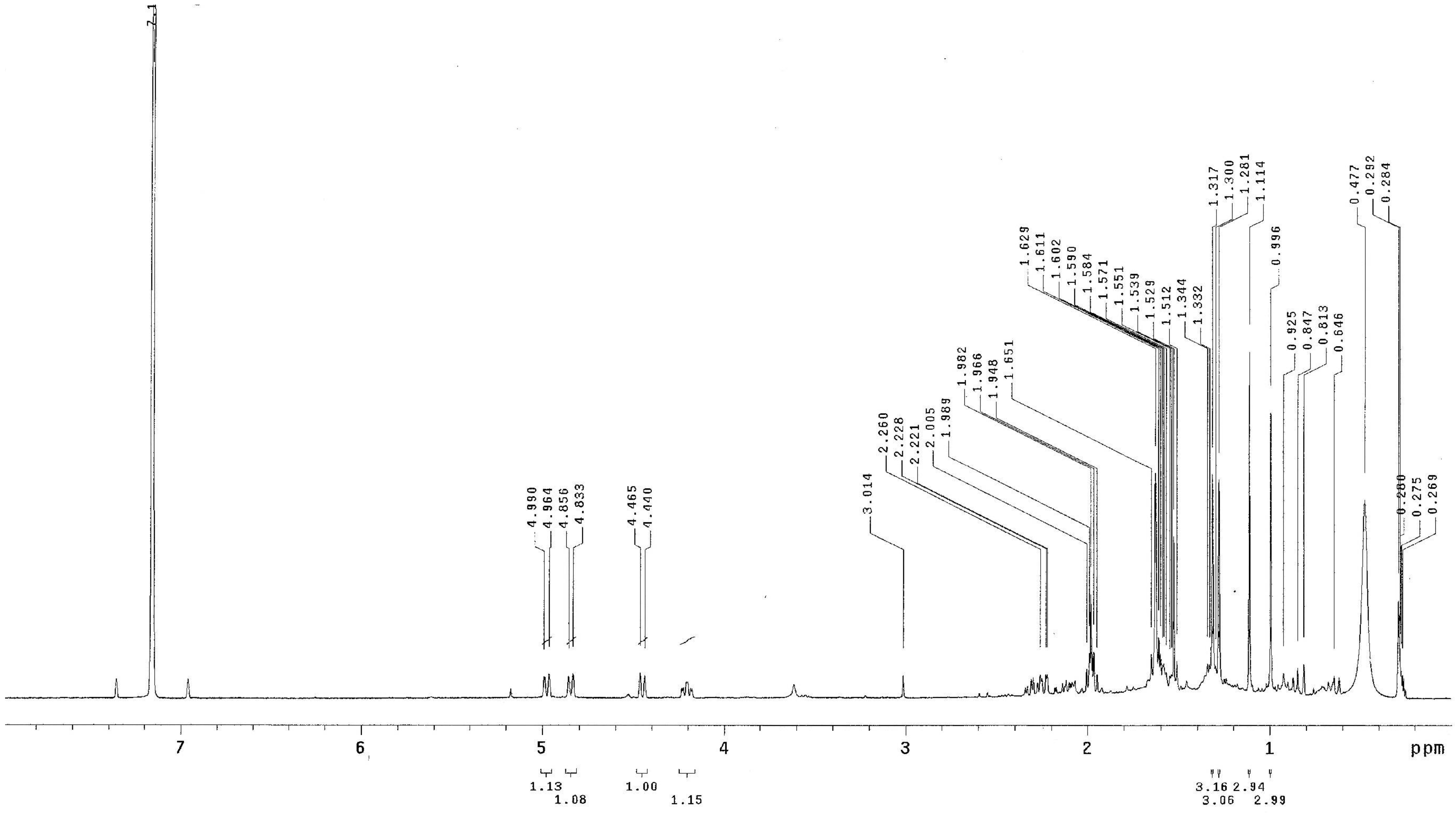

Figure S23. ^{1}H NMR spectrum of **4** in C_6D_6

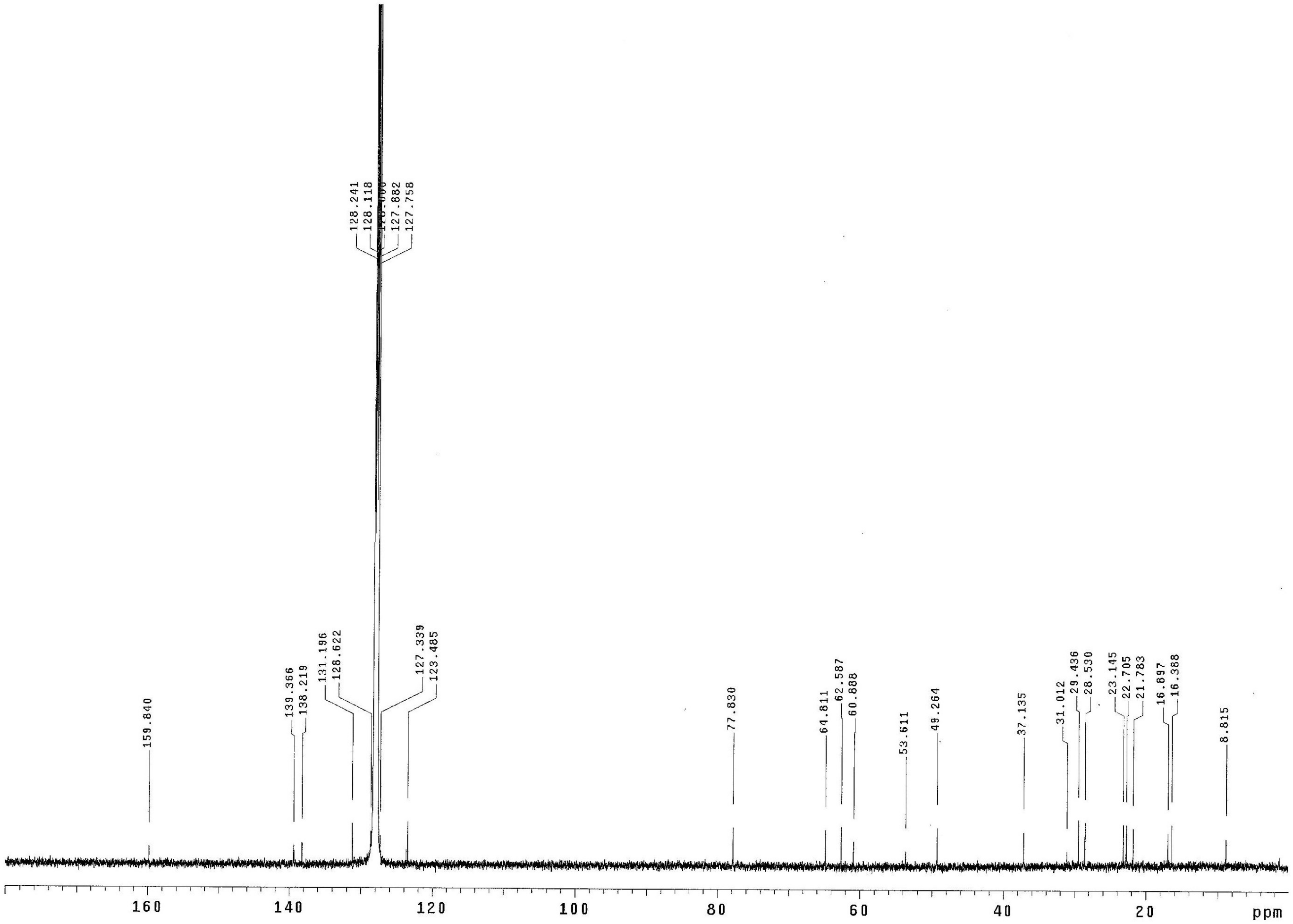

Figure S24. ^{13}C NMR spectrum of **4** in C_6D_6

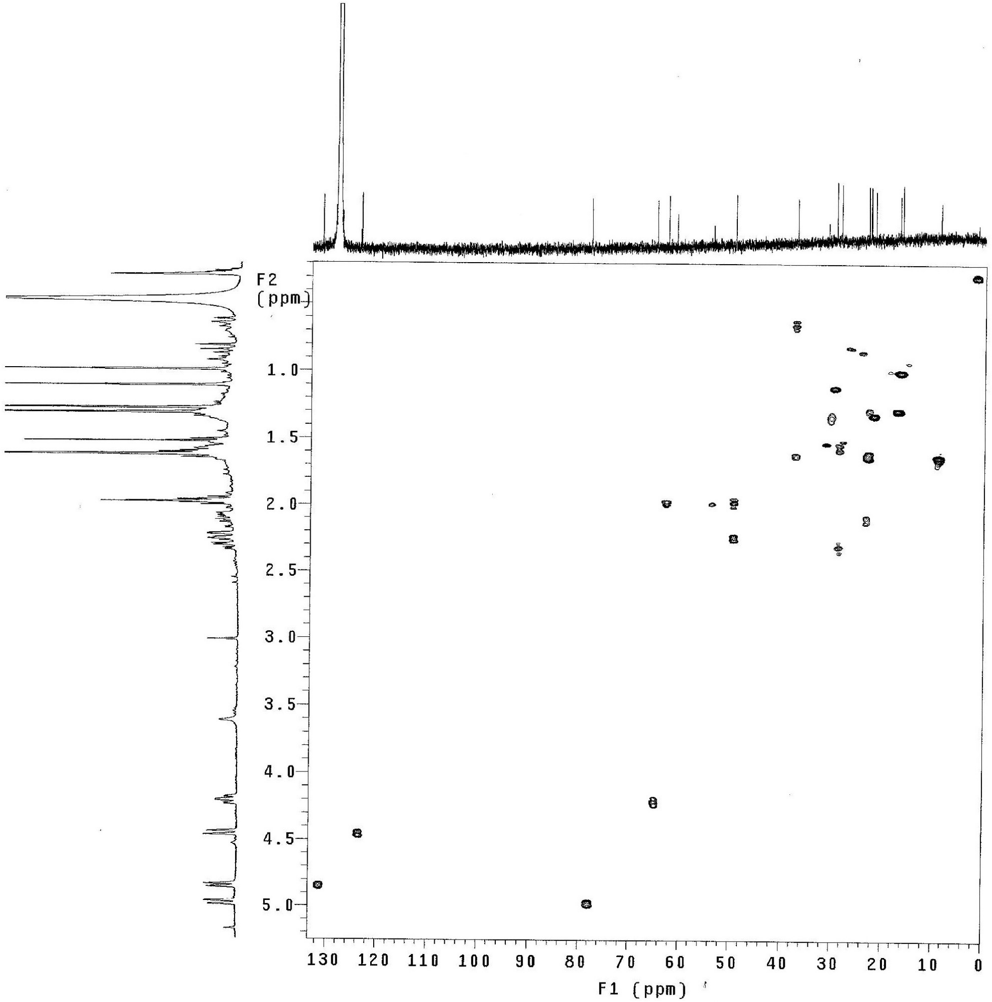

Figure S25. HSQC spectrum of **4** in C_6D_6

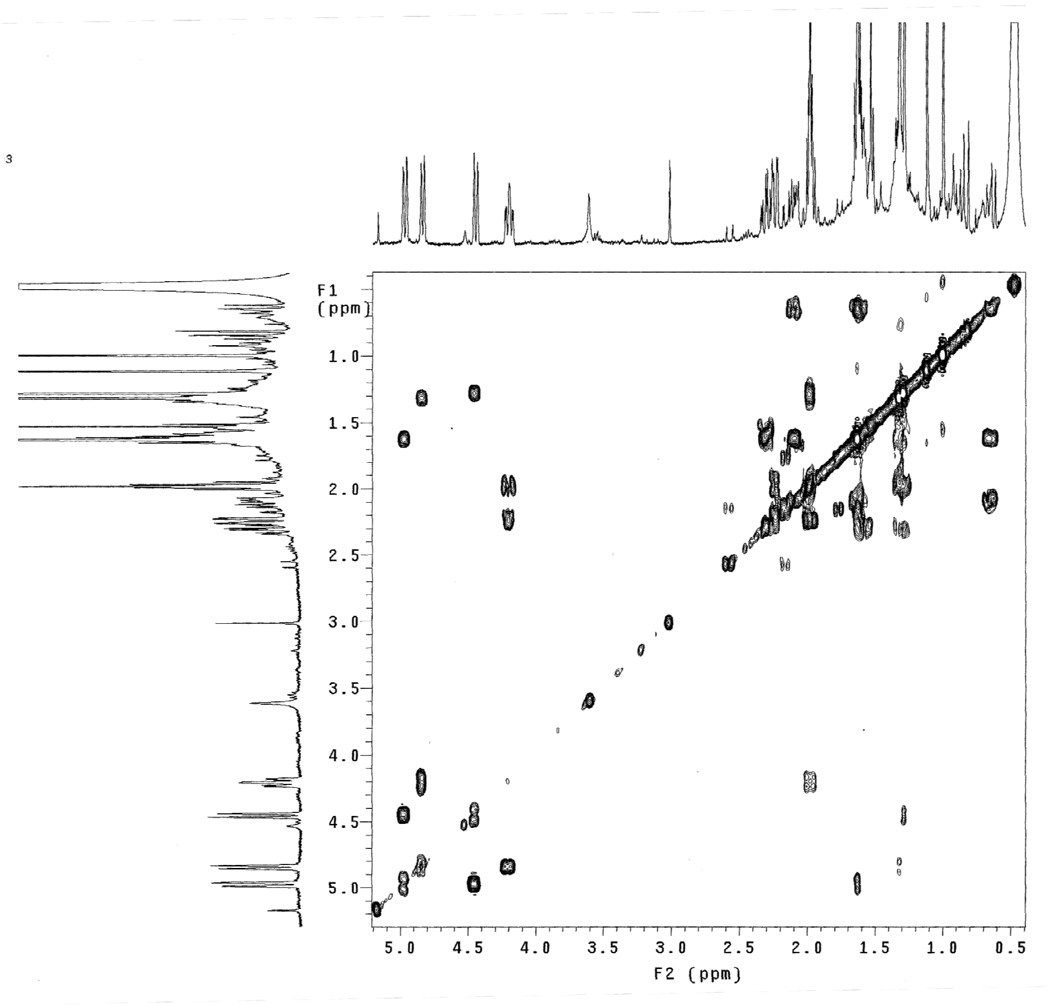

Figure S26. ^1H-^1H COSY spectrum of **4** in C_6D_6

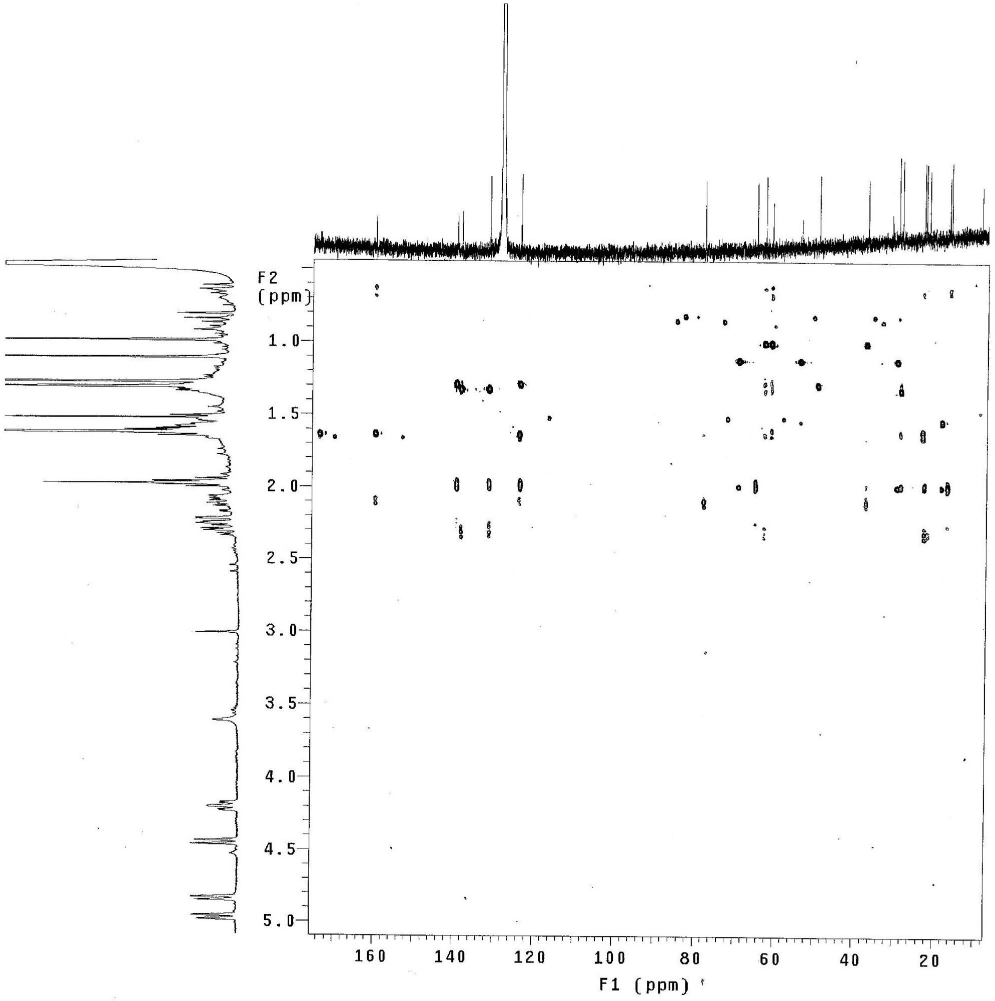

Figure S27. HMBC spectrum of **4** in C_6D_6

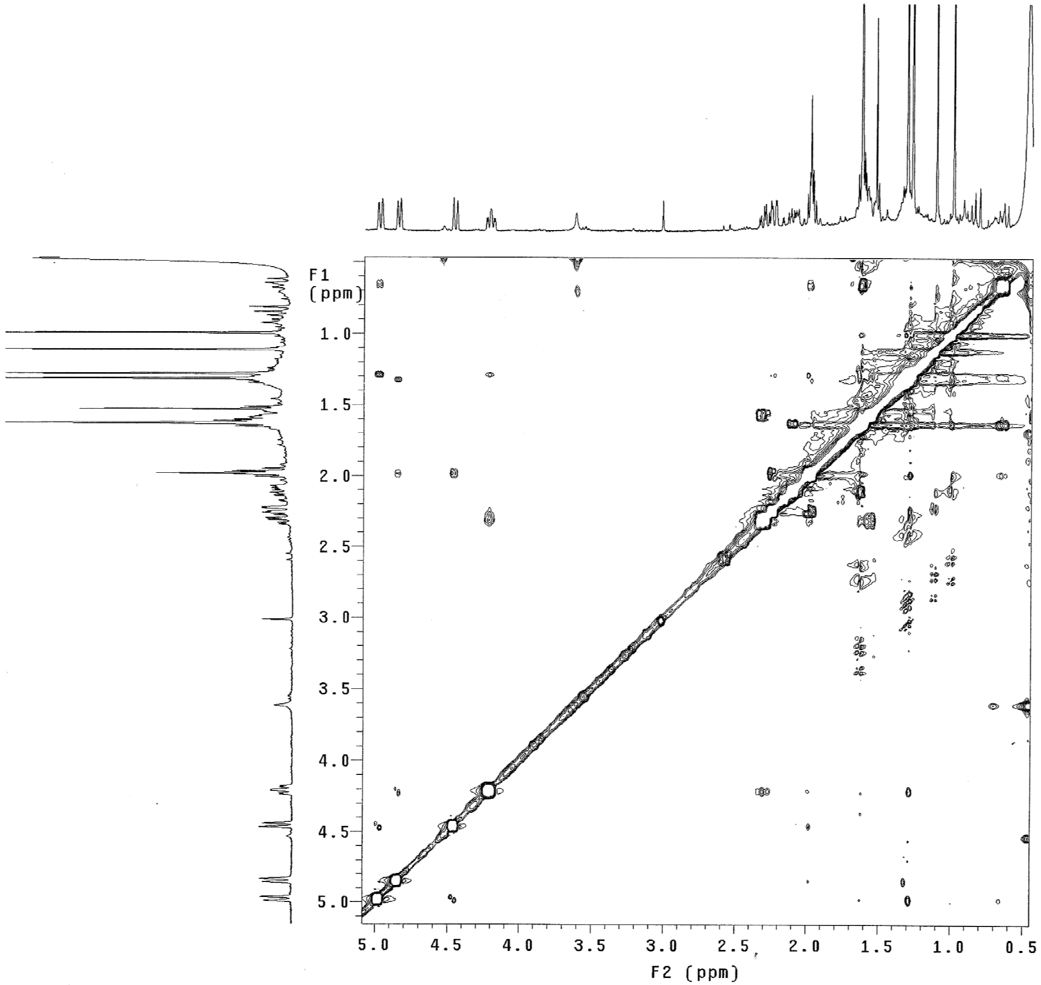

Figure S28. NOESY spectrum of **4** in C_6D_6

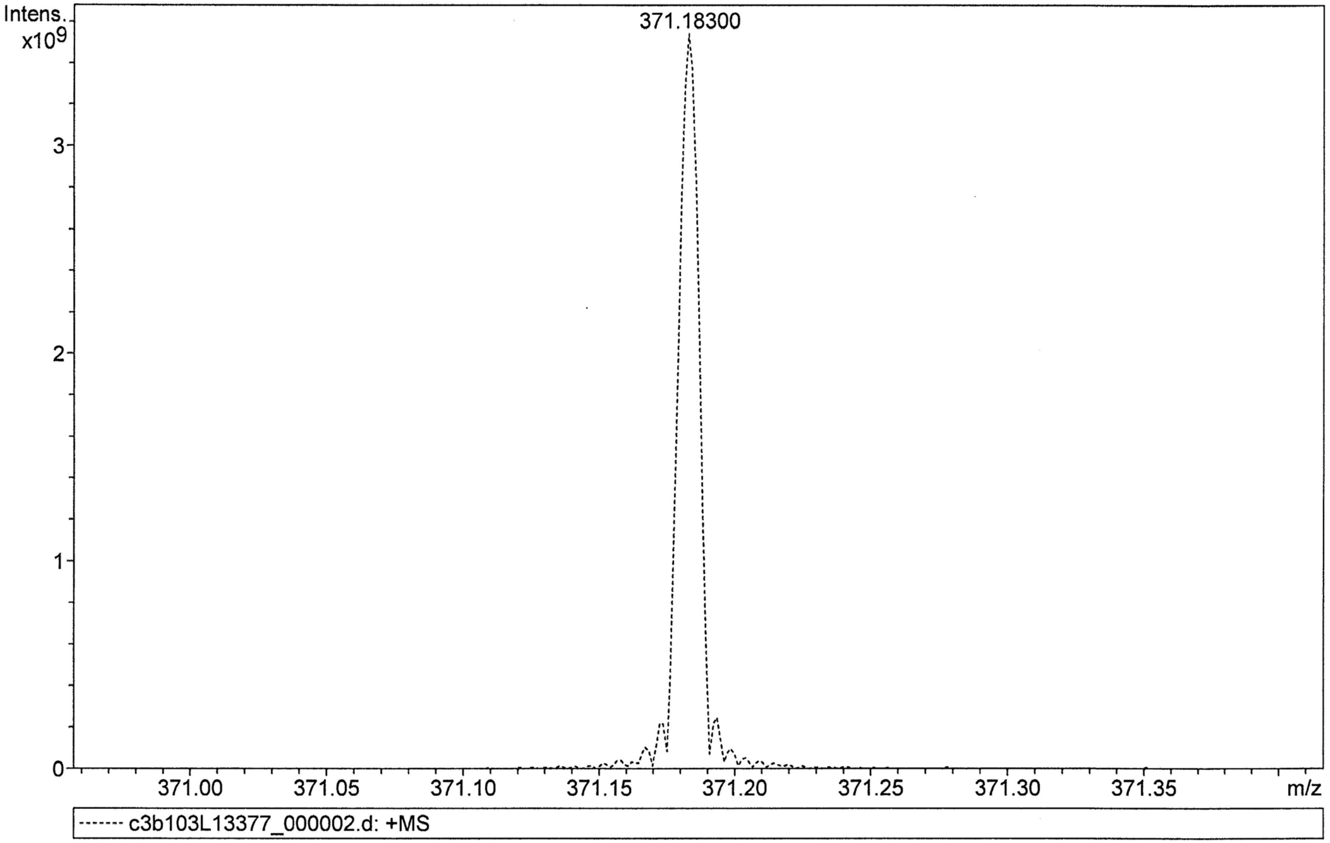

Meas. m/z	#	Formula	Score	m/z	err [mDa]	err [ppm]	mSigma	rdb	e⁻ Conf	N-Rule
371.18300	1	C 20 H 28 Na O 5	100.00	371.18290	-0.11	-0.29	6.6	6.5	even	ok

Figure S29. HREIMS spectrum of **5**

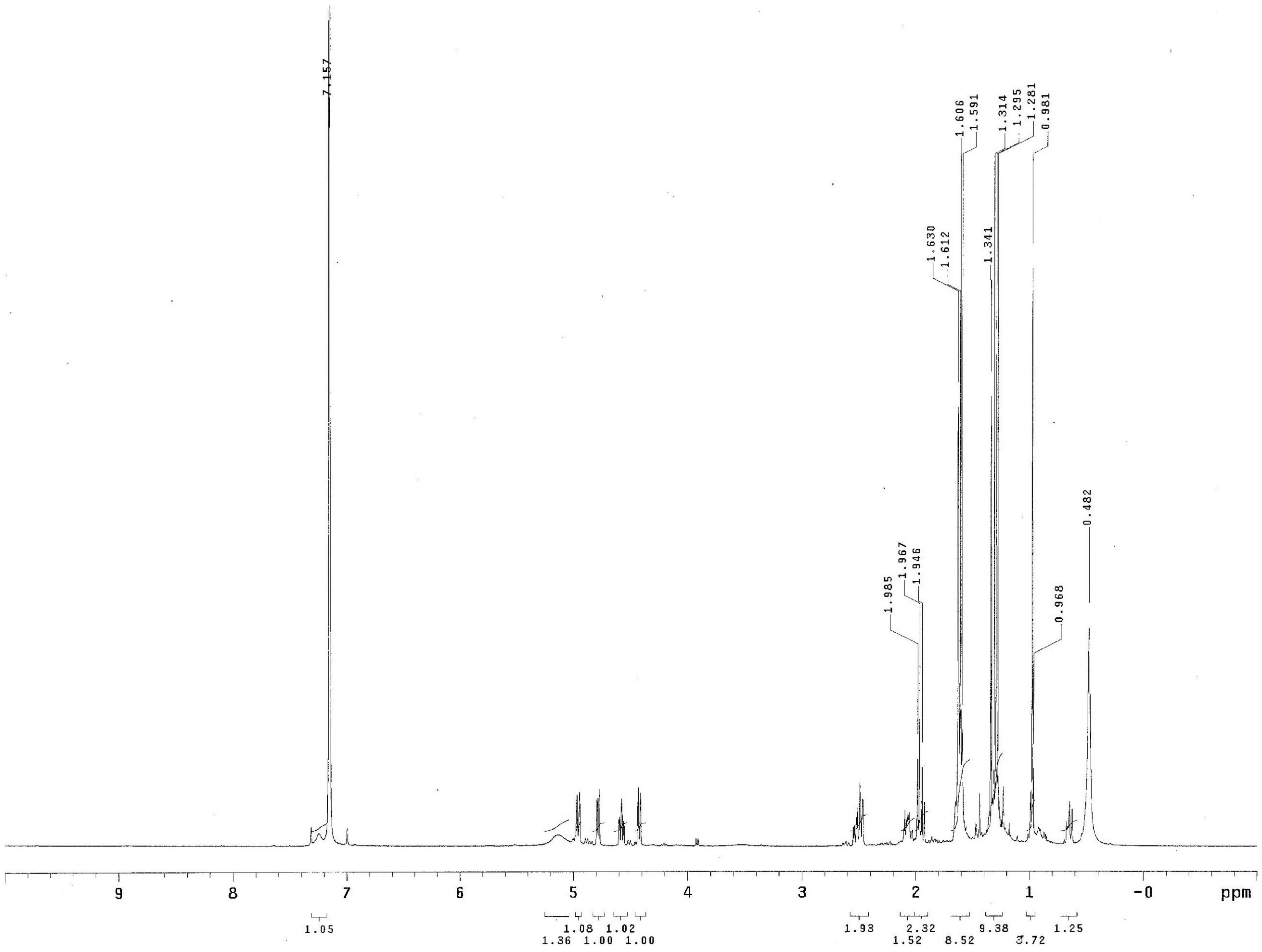

Figure S30. ^1H NMR spectrum of **5** in C_6D_6

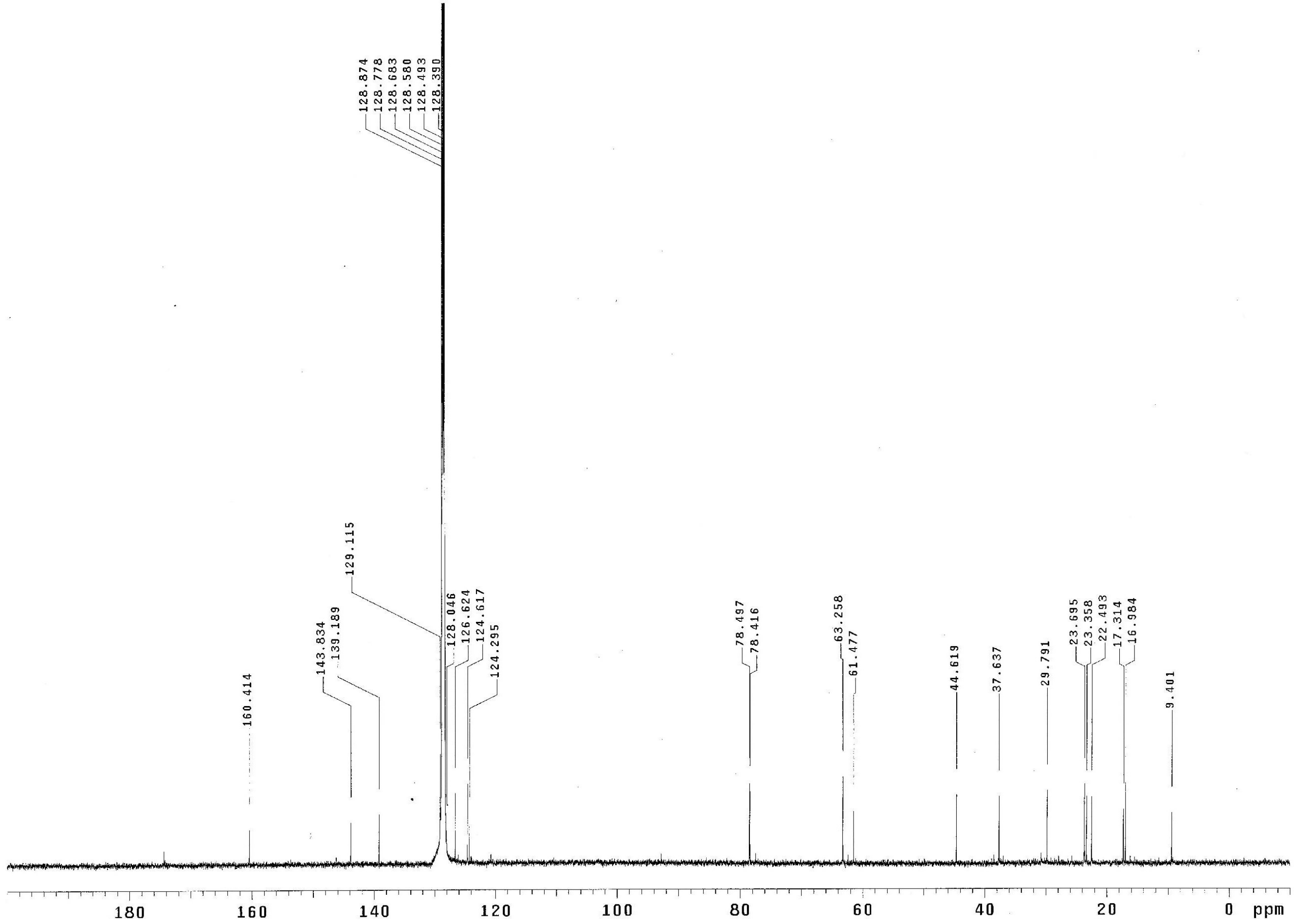

Figure S31. ^{13}C NMR spectrum of **5** in C_6D_6

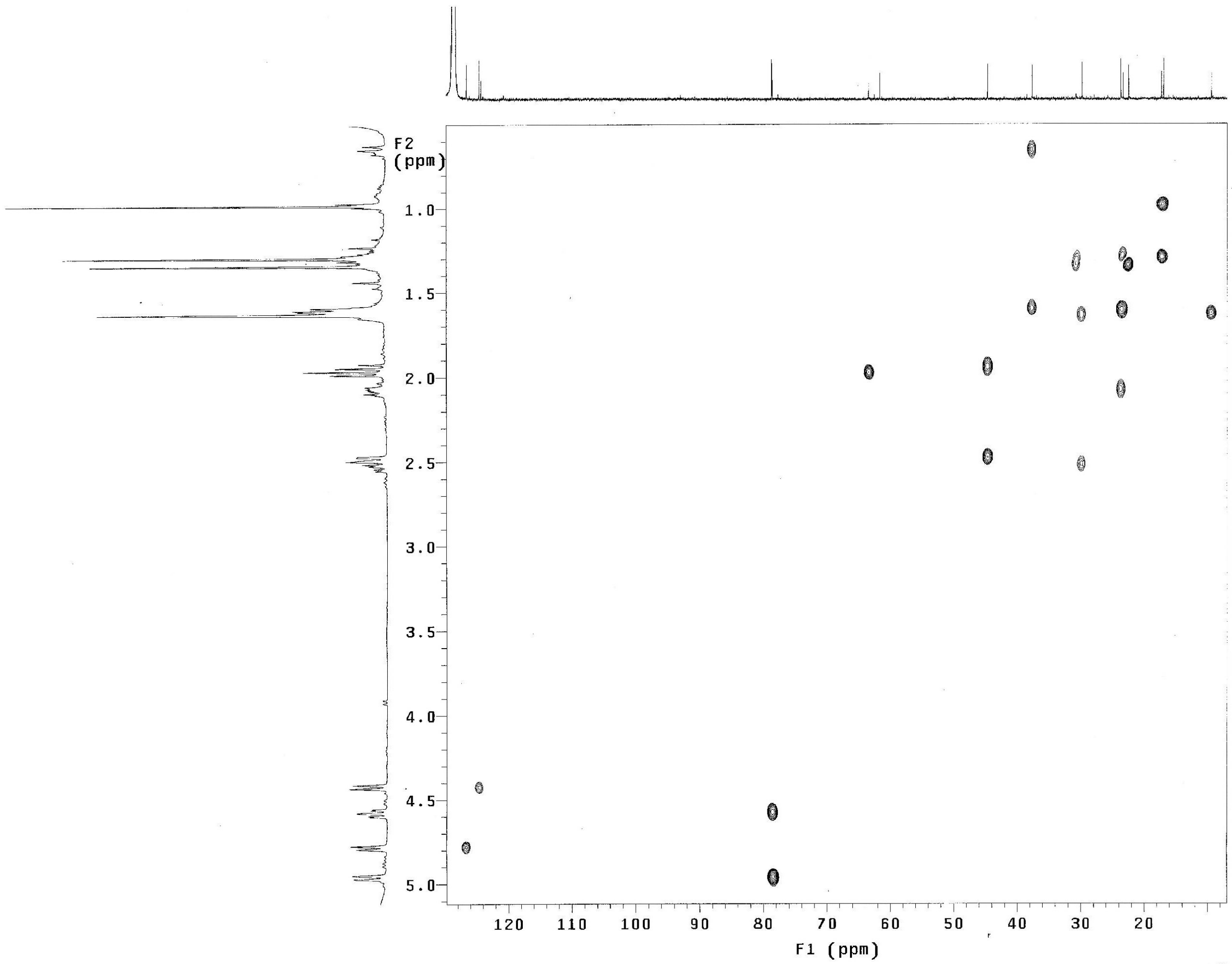

Figure S32. HSQC spectrum of **5** in C_6D_6

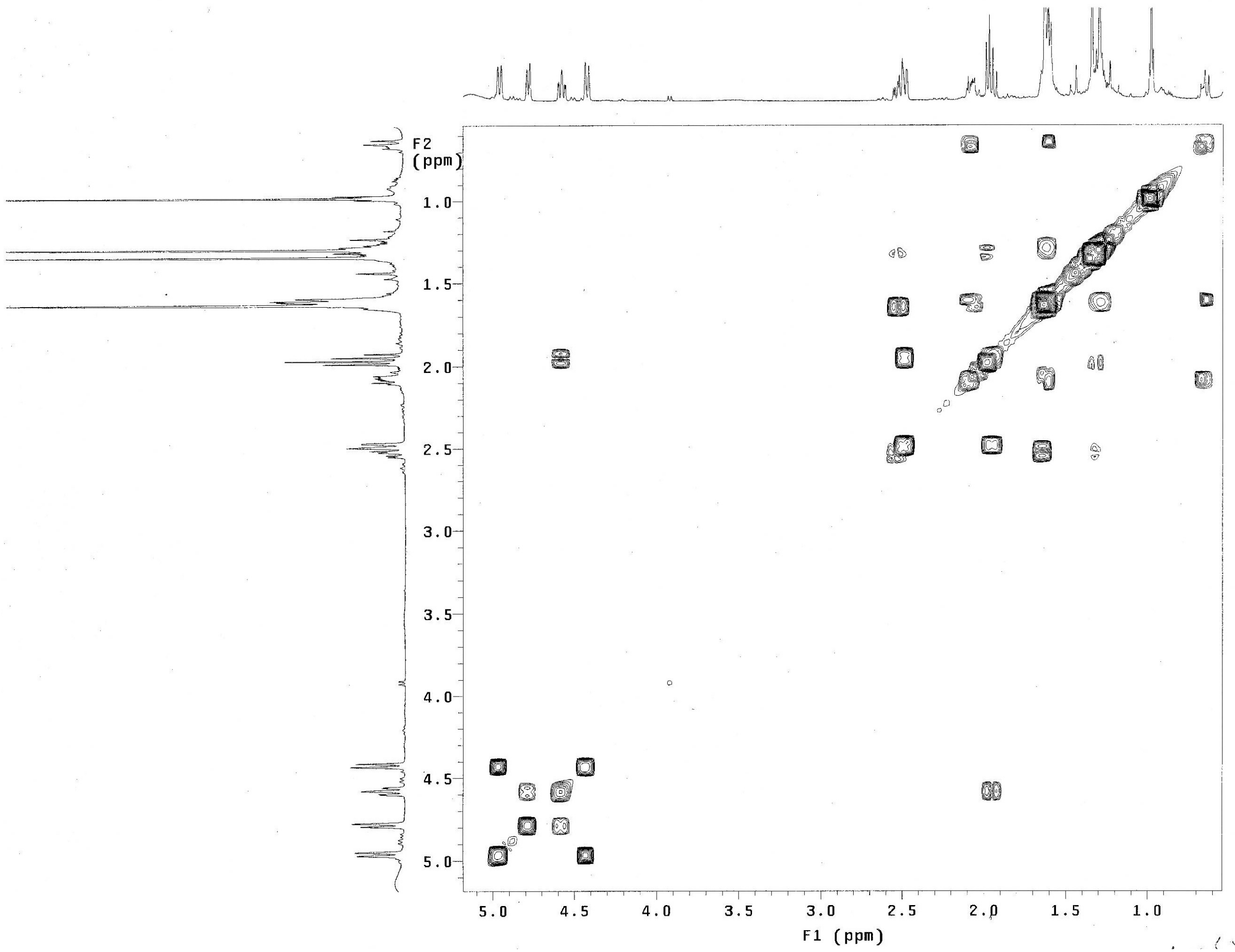

Figure S33. ^{1}H-^{1}H COSY spectrum of **5** in C_6D_6

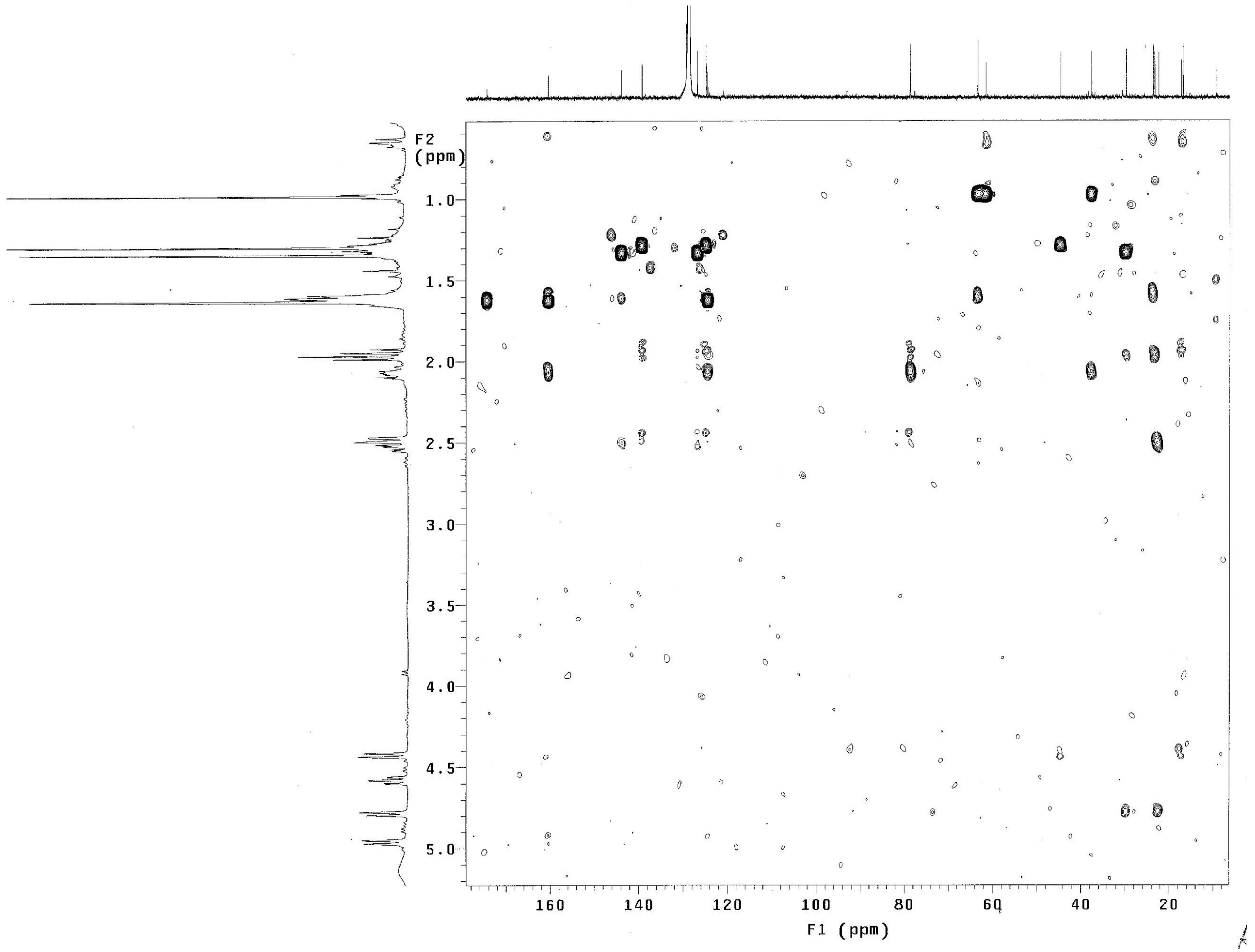

Figure S34. HMBC spectrum of **5** in C_6D_6

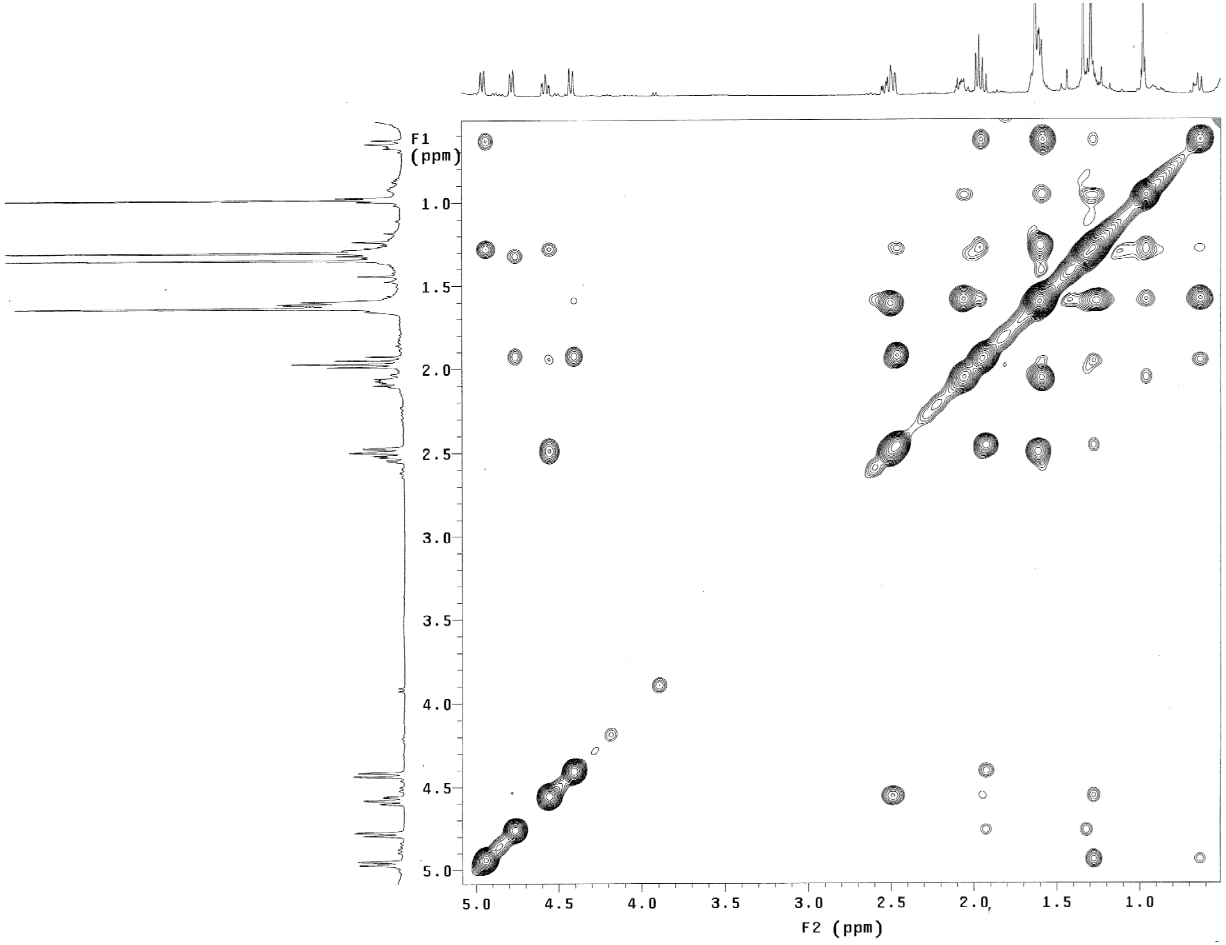

Figure S35. NOESY spectrum of **5** in C_6D_6

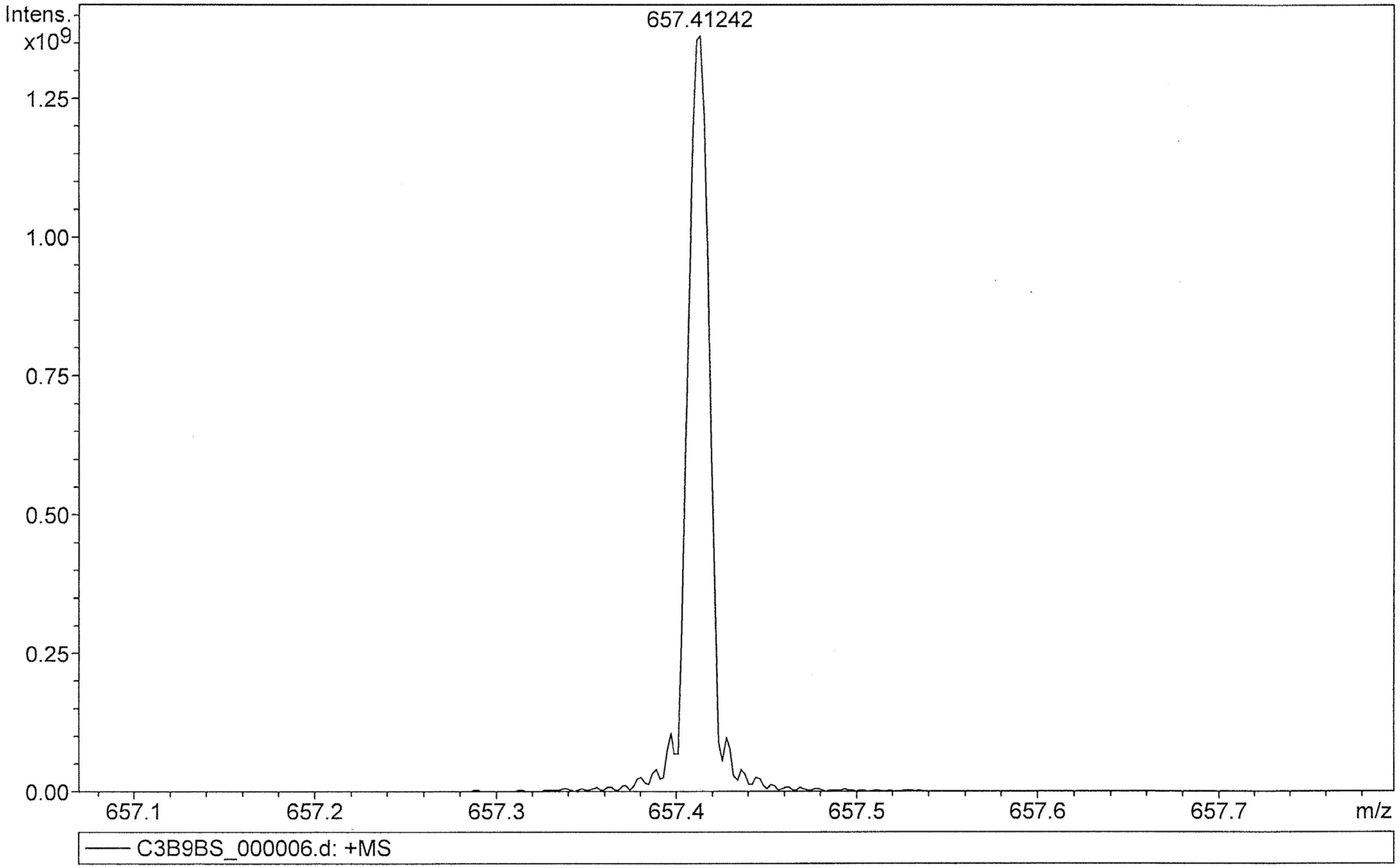

Meas. m/z	#	Formula	Score	m/z	err [mDa]	err [ppm]	mSigma	rdb	e⁻ Conf	N-Rule
657.41242	1	C 40 H 58 Na O 6	100.00	657.41256	0.14	0.22	16.2	11.5	even	ok

Figure S36. HRESIMS spectrum of **6**

FT-MS

Analysis Info

Analysis Name	D:\Data\a1\C3B9BS_000006.d	11/6/2015 10:45:00 AM
Method	broadband first signal	
Sample Name	C3b-9bs	Instrument: FT-MS solariX
Comment	ESI Positive	

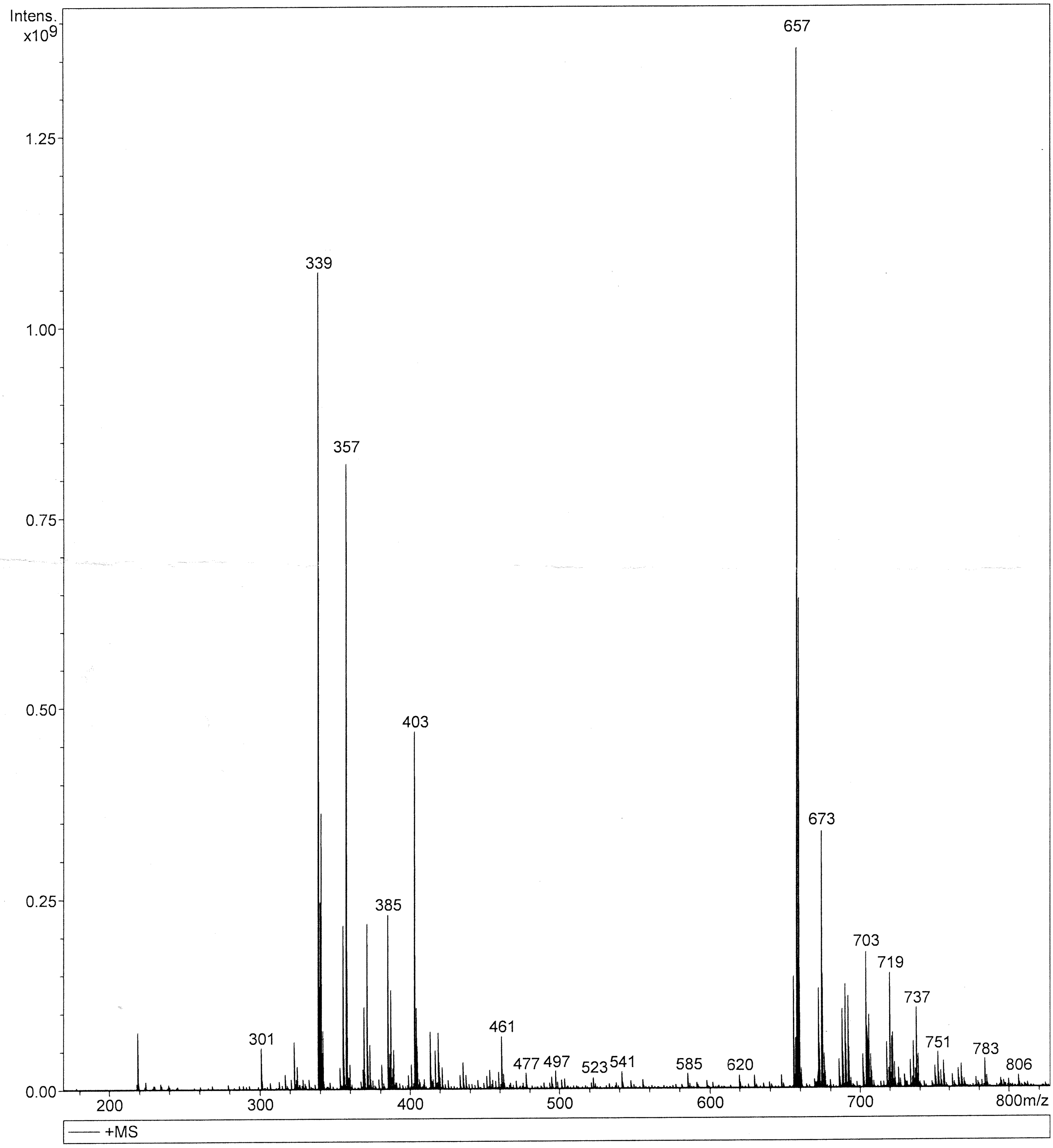

Bruker Compass DataAnalysis 4.0 printed: 11/6/2015 10:45:00 AM Page 1 of 1

Figure S37. ESIMS of **6**

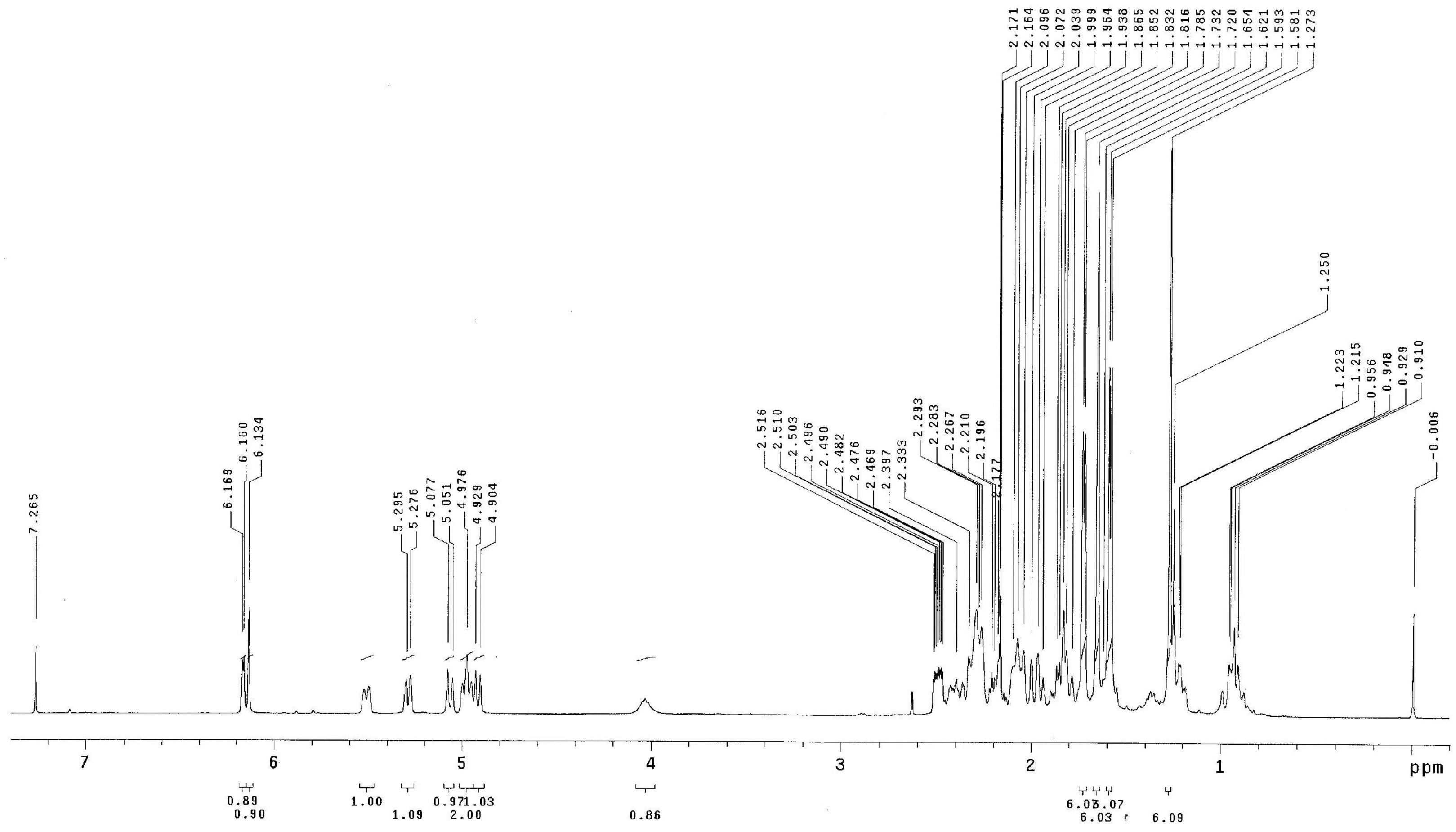

Figure S38. ^{1}H NMR spectrum of **6** in $CDCl_3$

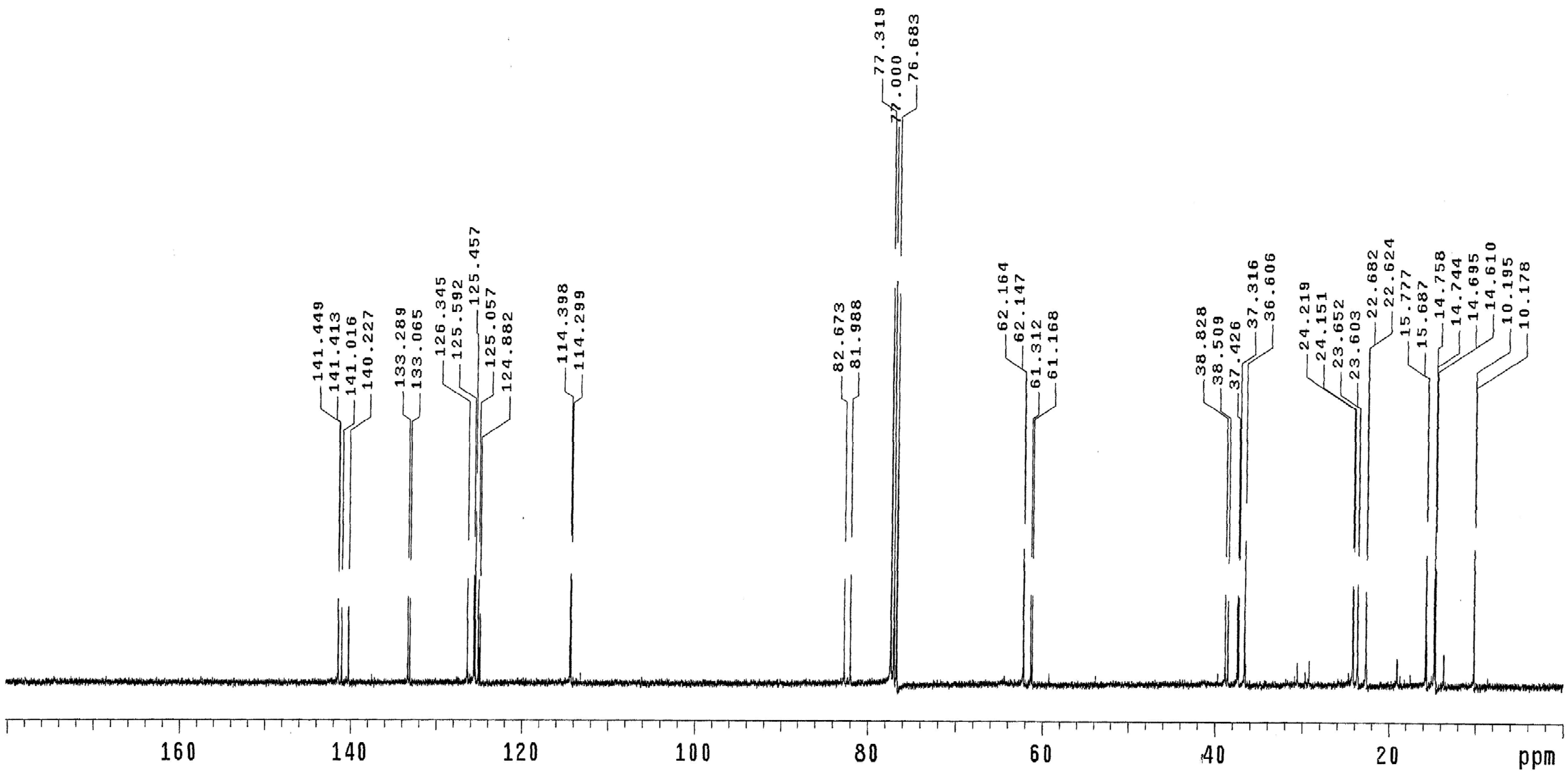

Figure S39. ^{13}C NMR spectrum of **6** in $CDCl_3$

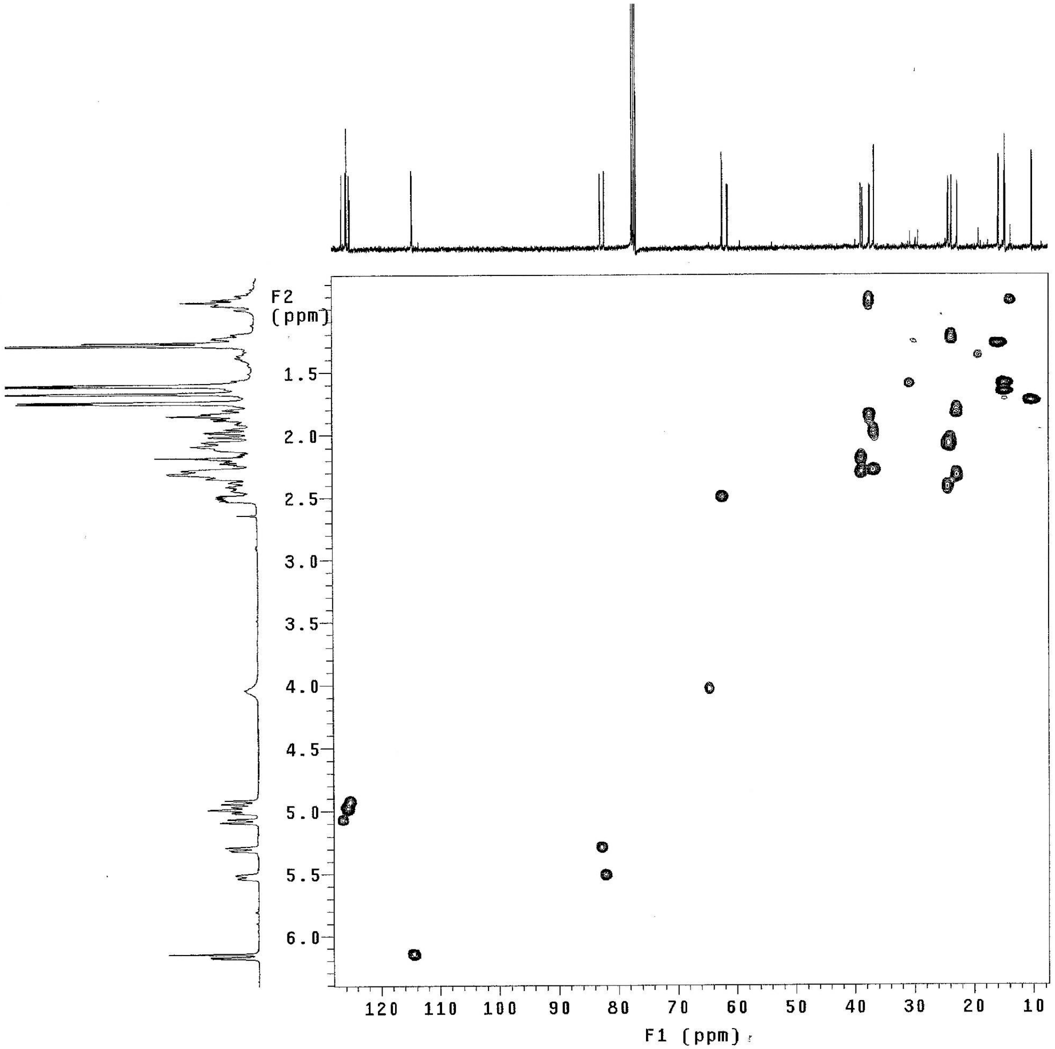

Figure S40. HSQC spectrum of **6** in $CDCl_3$

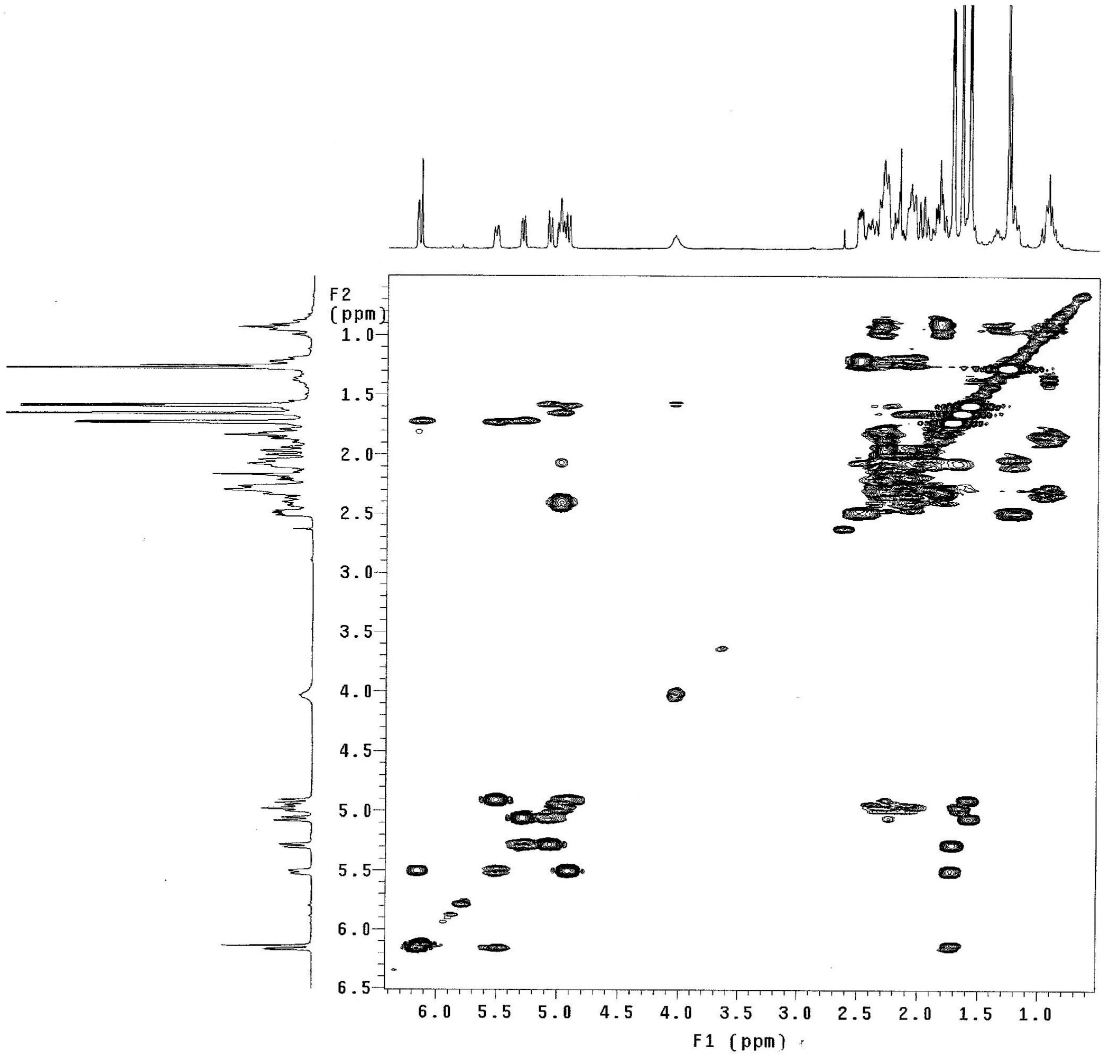

Figure S41. ^{1}H-^{1}H COSY spectrum of **6** in $CDCl_3$

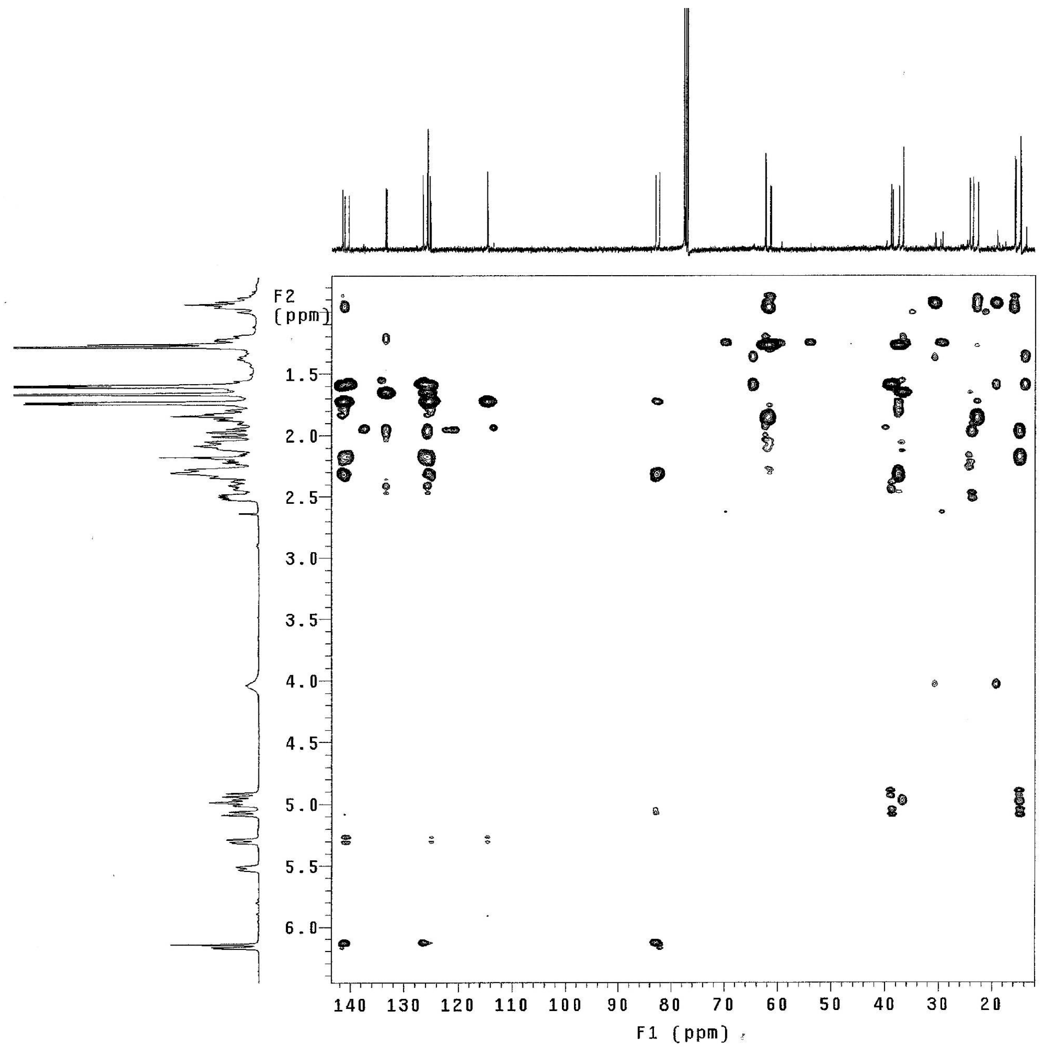

Figure S42 HMBC spectrum of **6** in $CDCl_3$

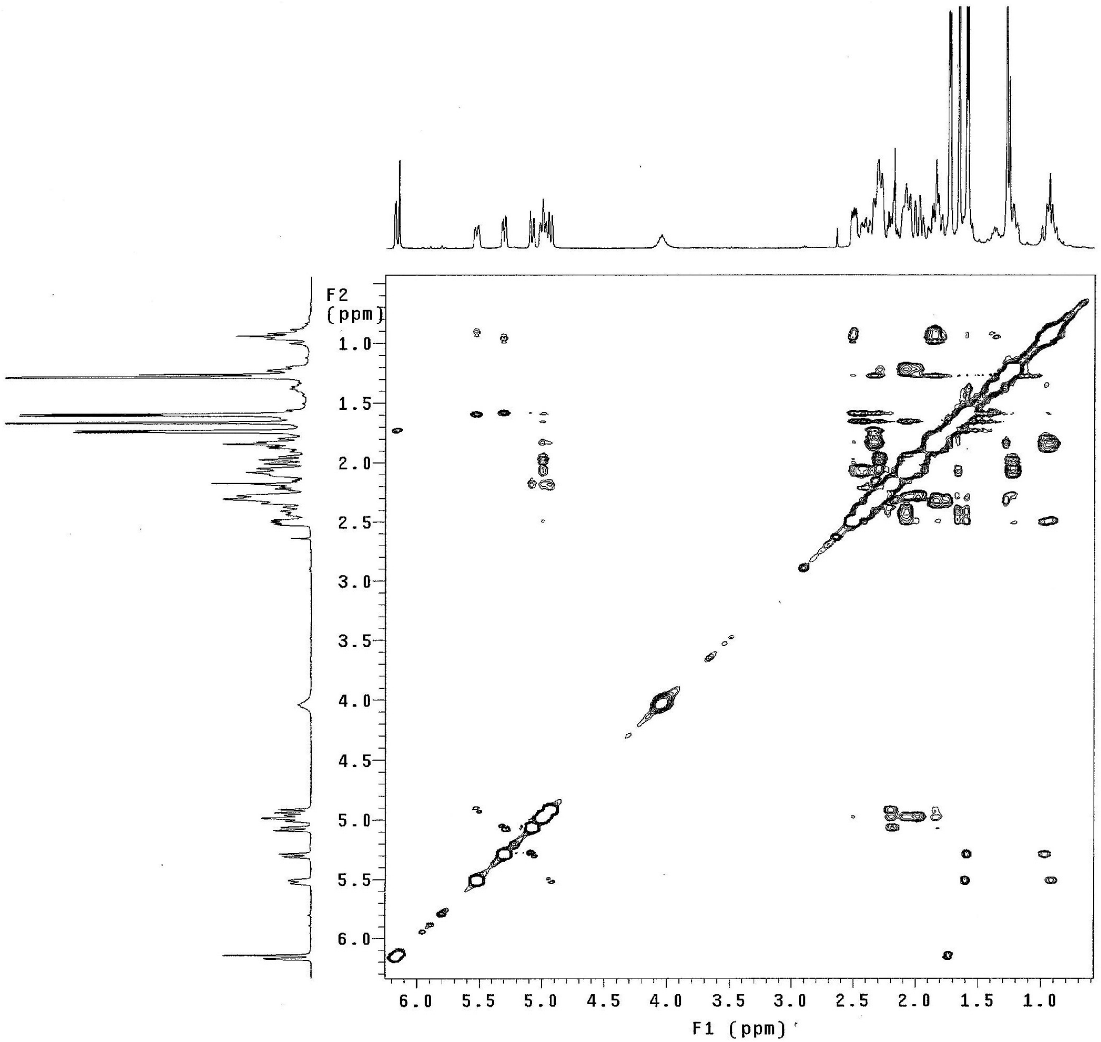

Figure S43. NOESY spectrum of **6** in $CDCl_3$

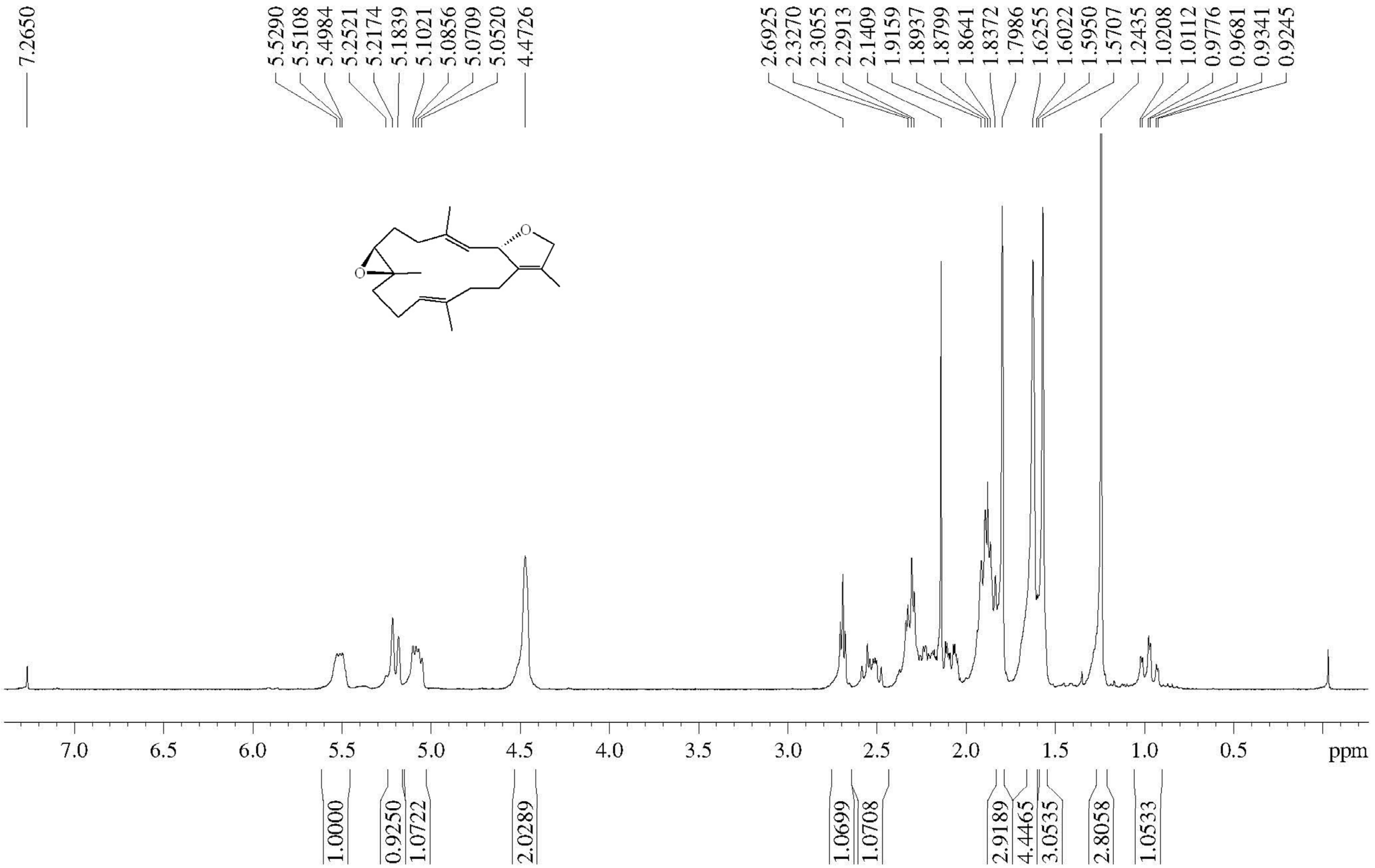

Figure S44. ^{1}H NMR spectrum of (+)-sarcophytoxide in $CDCl_3$ before treatment with acetone and silica gel under air

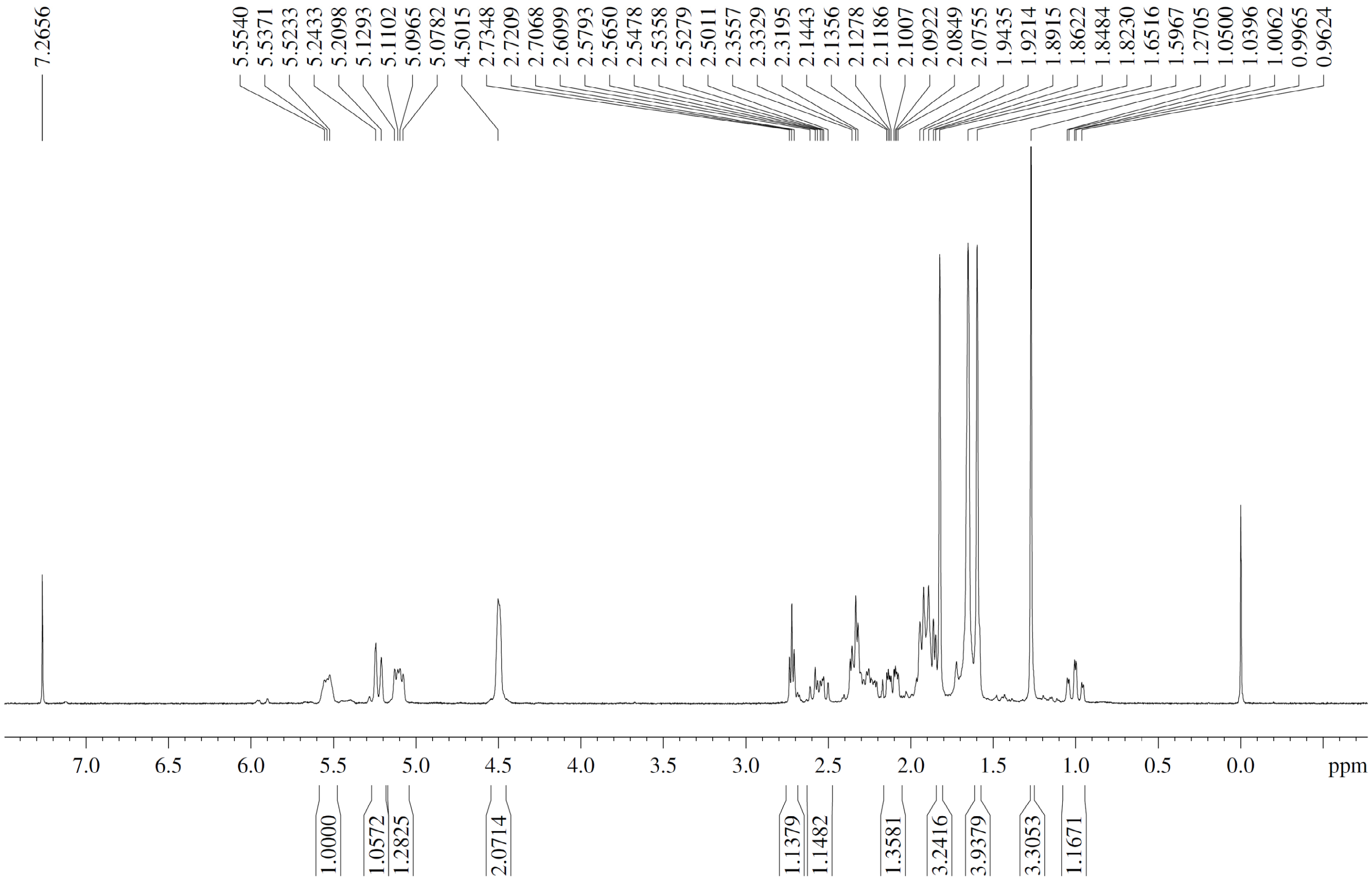

Figure S45. ^{1}H NMR spectrum of (+)-sarcophytoxide in $CDCl_3$ after treatment with acetone and silica gel under air

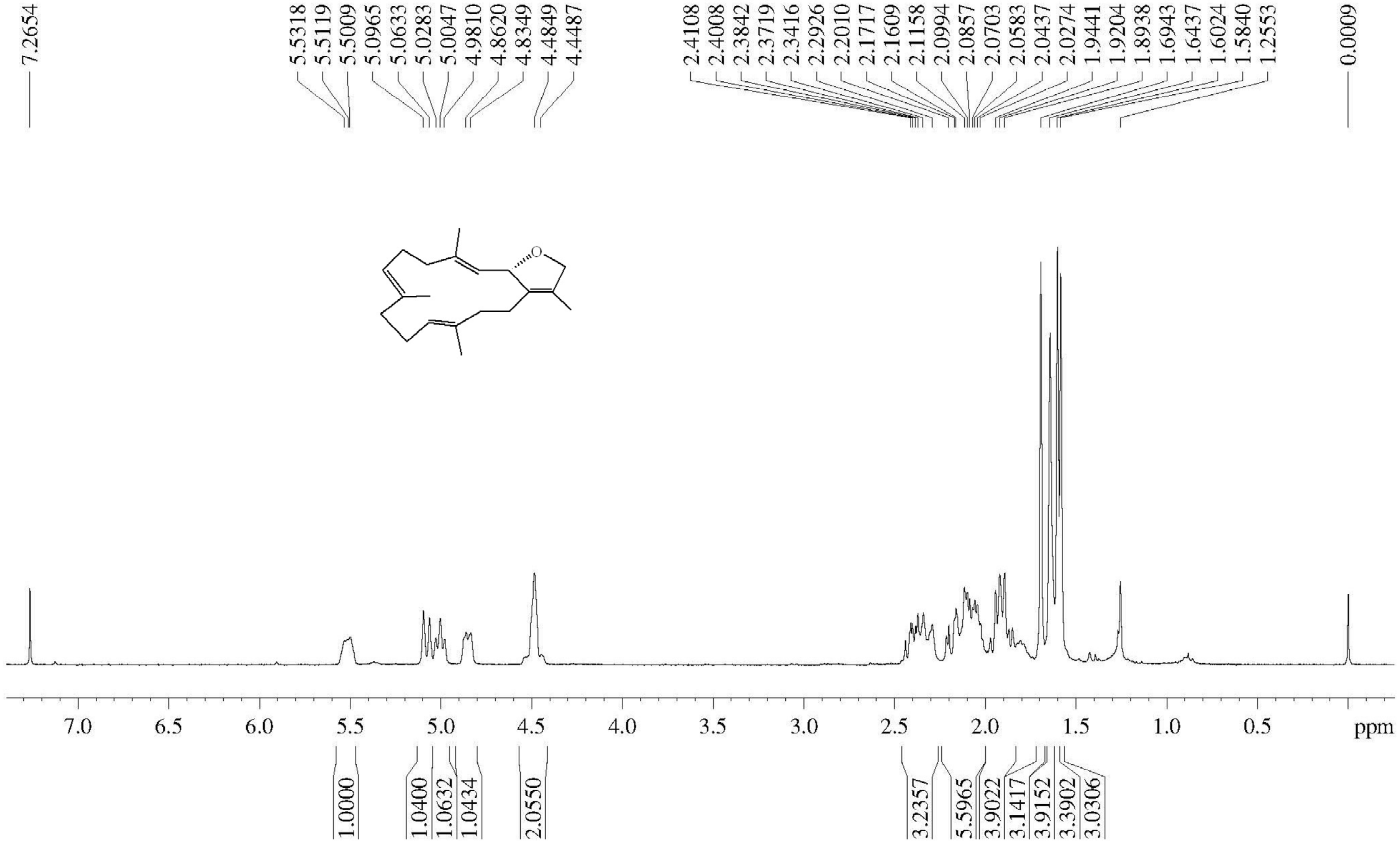

Figure S46. ^{1}H NMR spectrum of sarcophytonin A in $CDCl_3$ before treatment with acetone and silica gel under air

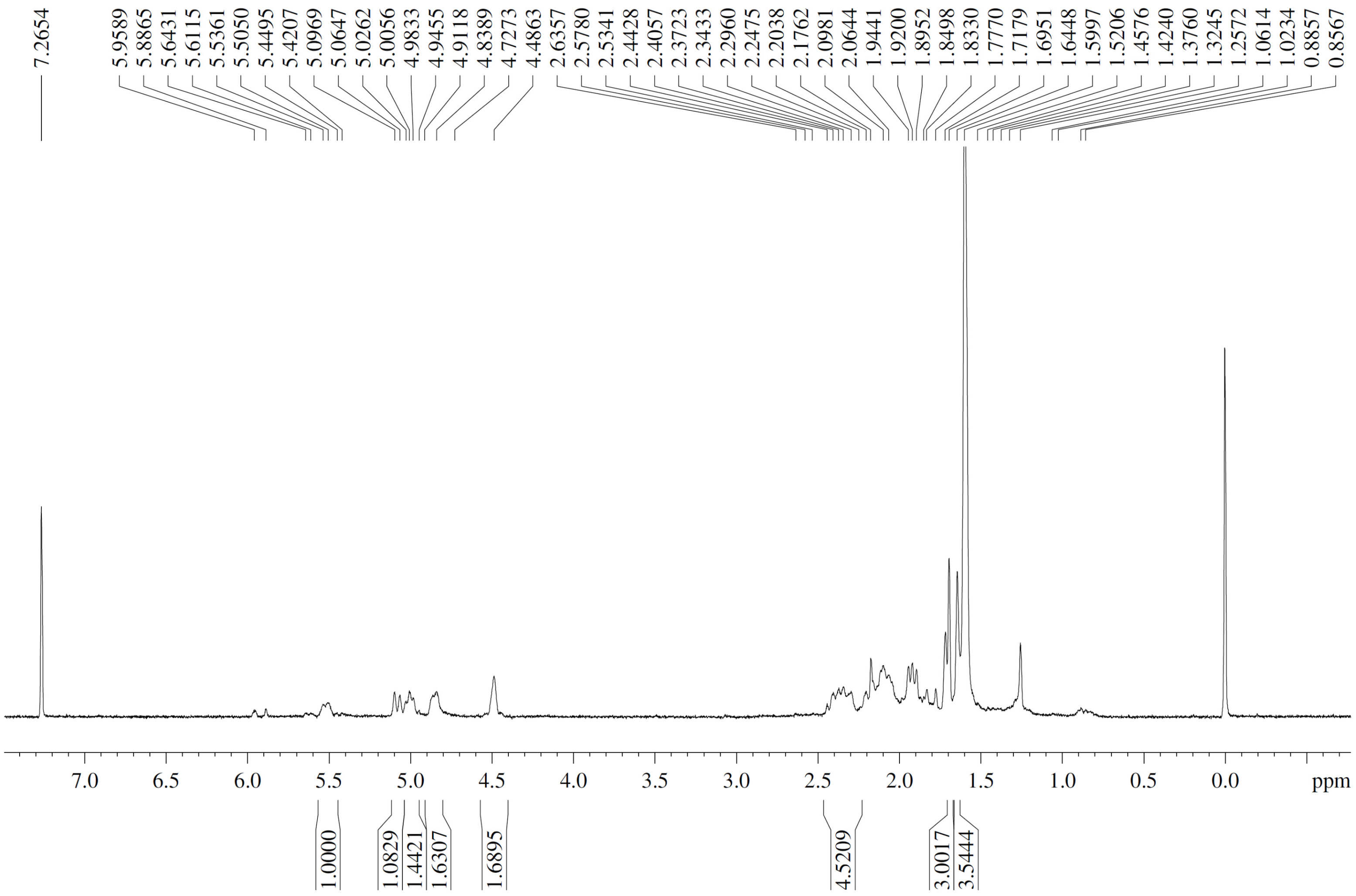

Figure S47. ^{1}H NMR spectrum of sarcophytonin A in $CDCl_3$ after treatment with acetone and silica gel under air
